# A Systematic Review of the Markers of Severity in Acute Respiratory Infections to Inform Primary Care Surveillance

**DOI:** 10.1111/irv.70172

**Published:** 2025-10-24

**Authors:** William H. Elson, Anna Forbes, Gavin Jamie, Rashmi Wimalaratna, Roger Morbey, F. D. Richard Hobbs, Simon de Lusignan, Jamie Lopez Bernal

**Affiliations:** ^1^ Nuffield Department of Primary Care Health Sciences University of Oxford Oxford UK; ^2^ Centre for Public Health, Faculty of Population Health Studies, Bristol Medical School University of Bristol UK; ^3^ Oxford Institute of Digital Health Oxford UK; ^4^ Immunisation and Vaccine Preventable Diseases Division UK Health Security Agency London UK

**Keywords:** electronic health records, influenza, human, primary health care, public health surveillance, respiratory syncytial viruses, respiratory tract infections, SARS‐CoV‐2, systematic review

## Abstract

**Background:**

Primary care computerised medical records (CMR) are used to report the incidence of acute respiratory infections (ARI) for public health surveillance. These systems could increase their utility by also reporting population‐level severity of ARI; however, this is rarely done.

**Objectives:**

To identify candidate markers of ARI severity suitable for use in primary care CMR‐based surveillance.

**Methods:**

We undertook a systematic review of bibliographic databases and grey literature. Eligible studies reported characteristics for > 500 patients with ARI, severe ARI, influenza‐like illness or suspected COVID‐19. Studies had to report at least one potential marker of severity. A panel of clinical primary care informaticians reviewed candidate severity markers and assessed each for severity, specificity, relevance to primary care and whether it was likely to be recorded in a CMR.

**Results:**

We included 126 studies from 84 countries. Seventy‐seven candidate severity markers were identified across 11 groups. These included four outcome groups (complications, hospital events, intensive care events and death) and seven predictor groups (symptoms, signs, scores, investigations, treatments, absenteeism and treatment‐seeking behaviour). Thirty markers were considered most suitable for primary care CMR‐based ARI surveillance: 7 outcomes (such as hospital admission, attendance and death) and 23 predictors (such as shortness of breath, oxygen levels, work absence and antibiotics). Predictors were generally considered more timely, as they are likely recorded during the consultation.

**Conclusions:**

This review provides a list of severity markers that could support the development of population‐level severity indicators for ARI surveillance in primary care. This could improve real‐time situational awareness during respiratory outbreaks.

## 1 Introduction

Primary care computerised medical records (CMRs) are increasingly used to support public health surveillance of acute respiratory infections (ARIs) [1, 2, 3]. By repurposing data collected during routine patient encounters, these surveillance systems generate incidence estimates of common respiratory clinical syndromes, including ARI and influenza‐like illness (ILI) [4].

Incorporating population‐level measures of clinical severity alongside these incidence outputs would increase the value of primary care ARI surveillance. This is because the burden exacted on society by a pathogen is determined by the disease severity as well as incidence. High incidence coupled with low severity may produce significant disruption in the community yet exert only minimal pressure on secondary care services, as was the case during the COVID‐19–Omicron BA.1/BA.2 wave in early 2022 [5]. Conversely, even moderate incidence with high severity can overwhelm hospitals and intensive care units (ICUs), such as occurred with the alpha variant in early 2021 [6].

Understanding severity early can help us predict the potential burden on healthcare services and intervene in a timely manner. For respiratory virus surveillance systems, reporting population‐level severity can inform public health interventions [7, 8]. For example, increases in surveillance severity indicators may predict an increase in hospital admissions or deaths. It may also help determine in which groups severe outcomes are occurring. Resources can therefore be directed to areas and populations of increased need, as occurred repeatedly during the COVID‐19 pandemic.

Hospitalisation, ICU admission and death are the standard markers of severe ARI in public health surveillance [9]. Capturing these outcomes promptly in primary care CMRs is challenging for two main reasons. Firstly, they arise late in the clinical course [10]. Secondly, in the UK, details of hospitalisation are typically entered into the primary care record only after the practice receives a hospital discharge summary, rather than at the time of admission, introducing additional delay [11].

The primary care CMR, however, is rich in clinical data that could serve to characterise ARI severity without relying on discharge summaries [12, 13]. For example, symptoms, signs and treatments recorded in CMRs can all help gauge how severe ARI infections are likely to be. For example, breathlessness may reflect respiratory compromise, which could be corroborated by abnormal clinical signs such as an increased respiratory rate or reduced peripheral oxygen saturation. Prescriptions, including antibiotics or steroids, may further indicate heightened clinical concern. Importantly, because clinicians usually enter these data contemporaneously, they are available more promptly than standard severe outcomes, such as hospitalisation.

This systematic review aims to identify a range of possible candidate severity markers that could be used to support the development of timely severity indicators for primary care CMR‐based ARI surveillance.

## 2 Methods

This systematic review was registered with PROSPERO (registration number CRD42023460281) and its protocol was made publicly available in August 2023 [14]. This is a descriptive, mapping‐style systematic review: it collates and summarises the severity markers reported in studies of ARI but does not evaluate intervention effects, compare groups or perform quantitative pooling. Consequently, no meta‐analysis or formal risk‐of‐bias appraisal was undertaken.

### 2.1 Background

This systematic review is part of a broader piece of work that aims to develop severity indicators for ARI surveillance at the Oxford Royal College of General Practitioners—Research and Surveillance Centre (RSC). The RSC is a CMR‐based infectious disease surveillance system that receives pseudonymised CMR data from over 2000 primary care practices in England, representing a third of the population [2, 15]. All UK primary care practices use Systematized Nomenclature of Medicine – Clinical Terms (SNOMED CT) to record clinical concepts in the primary care CMR. Primary care staff may also write free text in the record; however, the RSC does not receive free text data.

The RSC uses a phenotyping algorithm (case detection algorithm) to identify SNOMED CT encoded episodes of ARI from the CMR [2]. From these data, the RSC supplies the UK Health Security Agency (UKHSA) with weekly incidence reports for ARI and ILI [16]. This supports national respiratory surveillance efforts and is the main source of primary care respiratory surveillance data in England [17].

The systematic review initially aimed to identify all potential candidate markers from the literature. A subsequent review of the candidate markers by a team of primary care informaticians was then conducted in order to refine the candidate list of severity markers to those most suitable for use in primary care CMR‐based ARI surveillance. These markers will then be further tested in subsequent studies.

### 2.2 Eligibility Criteria

We included English‐language cross‐sectional or cohort studies published after 1 January 2009 that analysed surveillance or CMR data for more than 500 patients diagnosed with ILI, ARI, severe acute respiratory syndrome (SARI) or suspected COVID‐19. Because multiple definitions exist for these syndromes, we accepted each study's own case definition when determining eligibility.

While the focus of this study was on primary care, we included studies from any setting (community, primary care, hospital or ICU) for two main reasons. Firstly, outcomes measured in other settings could also be relevant in primary care. Secondly, candidate variables were subsequently reviewed by primary care informaticians specifically to assess their potential value for use in primary care CMR‐based ARI surveillance.

Inclusion criteria‐

- Population
  Populations with one of the following respiratory clinical syndromes:
    - Influenza‐like illness (ILI)
    - Acute respiratory infection (ARI)
    - Severe acute respiratory infection (SARI)
    - Suspected COVID‐19
  Sample size > 500 participants
  Any care setting (community, primary care, secondary care, ICU)
- Severity markers
  Study must report an outcome that could serve as a marker of clinical severity (e.g., symptoms, signs, complications, treatment, hospitalisation or death)
- Data source
  Analysis of routine surveillance data or CMR data
- Study design
  Cross‐sectional or cohort studies (prospective or retrospective)
- Time period
  Articles published on or after 1 January 2009
- Article type
  Peer‐reviewed manuscripts and surveillance reports from grey literature.
    Exclusion criteria‐
- Population
  Neonatal‐only or pregnancy‐only cohorts
  Populations restricted to specific comorbid groups (e.g., HIV‐positive)
- Study design
  Randomised controlled trials, case–control studies, reviews or systematic reviews, methodological papers without new data, case reports or case series
- Article type
  Conference proceedings, abstracts, preprints and letters to the editor were excluded to ensure inclusion of higher‐quality, peer‐reviewed full articles.

### 2.3 Information Sources

We searched three bibliographic databases via Ovid: MEDLINE, Embase and Global Health. In each database, we paired free text keywords with controlled vocabulary: Medical Subject Headings (MeSH) in MEDLINE, Emtree in Embase and Centre for Agriculture and Bioscience International (CABI) thesaurus terms in Global Health. Searches were conducted on the 5th of June 2023.

We searched the grey literature for 2018–2023 using the main search function on the websites from five public health bodies: the United States Centers for Disease Control and Prevention (CDC); the World Health Organization (WHO) Disease Outbreak News; UKHSA; European Centre for Disease Prevention and Control (ECDC); and the Sentiworld sentinel‐network repository, screening all retrieved titles and bulletins for relevant human‐respiratory surveillance content. Grey literature searches were conducted on the 15th March 2024.

Peer‐reviewed publications were included to ensure methodological quality, whereas grey literature sources were limited to official surveillance reports, which were considered important for contextualizing severity markers despite not being peer‐reviewed.

### 2.4 Search Strategy

Full search strategies can be seen in Appendices 1–4. The search strategy was built around three concepts:

Public health surveillance

This restricted retrieval to surveillance‐focused records by combining surveillance subject headings (e.g., Public Health Surveillance, Sentinel Surveillance) with free‐text terms that frequently signal syndromic surveillance—namely ILI, ARI and SARI.

Respiratory infectious disease

This combined controlled headings and free‐text synonyms for influenza and for SARS‐CoV‐2/COVID‐19. The SARS‐CoV‐2 block was date‐limited (≥ 2020) to exclude pre‐pandemic coronavirus literature. The union of the influenza lines and the restricted COVID‐19 lines constituted the target‐infection set. Previously peer‐reviewed search terms for Severe Acute Respiratory Syndrome Coronavirus 2 (SARS‐CoV‐2) were used.

Clinical severity

This combined subject‐heading terms such as Hospitalisation, Intensive Care Units, Mortality and Severity of Illness Index with free‐text expressions for severity markers. For example, ‘hospitali?ation rat*’, ‘fatality rat*’, ‘clinical* sever*’. Where * represents any number of characters and ? represents any one character.

Each concept combined search terms and free‐text using the *OR* operator. The three concepts were then combined using the *AND* operator. Further filters limited the studies to those published in English after 2009 and those involving only human participants.

### 2.5 Study Selection

Records were initially imported into Endnote, where the automatic deduplication tool was employed. Subsequently, records were uploaded to Rayyan (https://www.rayyan.ai/). Deduplication was also performed using Rayyan's inbuilt deduplication tool and followed up by deduplication by hand after title and abstract screening. Rayyan then served as the workspace for both the title and abstract screening and the subsequent full‐text review.

The titles and abstracts of articles identified during the search process were screened in duplicate by two independent authors (WE, AF), and conflicting judgments were resolved by discussion between themselves, and if necessary, a third author. Articles that passed the first screening went on to a full‐text review (WE, AF). Duplicate screening of the full‐text articles was also undertaken, with conflicts resolved by discussion, and if necessary, a third reviewer.

To reduce subjectivity when deciding what qualified as a candidate severity marker, reviewers considered the following:

On balance, might the presence (or absence) of this characteristic in an individual with ARI predict a severe outcome or represent a severe outcome?

Two reviewers answered this question independently for each study, recording their judgement. Discrepancies were reconciled through discussion, and with a third reviewer if needed. Reviewers 1 and 3 (WE, GJ) are practising primary care physicians. Reviewer 2 (AF) is a practising secondary care physician. This uniform process ensured that the classification of severe outcomes was as transparent as possible.

### 2.6 Data‐Collection Process

A bespoke electronic data entry form was built in JotForm to standardise extraction: dates were entered via a picker, categorical items chosen from predefined lists, free‐text boxes captured extra context and skip logic plus validity checks (e.g., publication date could not precede data collection period) reduced errors. The form was piloted on 10 studies by WE and AF and revised as needed.

Two reviewers (WE, AF) then extracted data independently in JotForm. Submissions were compiled into a cloud spreadsheet labelled by reviewer; discrepancies were resolved through discussion, with a third reviewer if required. The reconciled dataset was exported from JotForm as a CSV file. Study numbers are reported in a PRISMA flow diagram.

### 2.7 Data Items

We extracted three main groups of variables from each included study (Appendix 5).

**Study details:** captured the citation, study aim, publication type and date, study period and geographic scope.
**Case data:** recorded the respiratory syndrome under study (ARI, ILI, SARI, suspected COVID‐19 or other), the case definition applied, the number of cases and whether cases were nontreatment‐seeking, treatment‐seeking, hospitalised or in ICU. We grouped participants recruited at a medical facility but not admitted under the label ‘treatment‐seeking’, as, in many regions, the distinction between outpatient clinics, emergency departments and primary care services is not clear (Appendix 5). Nontreatment‐seeking patients were those who had not attended health care services; typically those identified in the community through surveys.
**Severity markers:** every variable proposed as a severity marker was recorded: clinical scores, symptoms, signs, laboratory results, complications, treatments, hospitalisation, death. Severity markers were classified as either (1) a severe outcome itself—such as death, a major complication or hospitalisation—or (2) a potential predictor of severity, for example, clinical signs or laboratory findings.

### 2.8 Data Synthesis

Extracted outcomes were first tabulated for every included study and grouped into broad groups (e.g., symptoms, vital signs, investigations, complications, treatment, hospitalisation, ICU admission, death). We then produced counts and percentages of studies reporting each domain, stratified by recruitment type (non treatment‐seeking, treatment‐seeking, hospital, ICU). These data are presented as frequency tables by recruitment type and a bar chart.

### 2.9 Primary Care Informatics Review

The resulting list of candidate severity markers was subsequently reviewed in two focused meetings by four primary care clinical informaticians (WE, GJ, RW, SdeL), all experienced in CMR‐based ARI surveillance. For each marker, the panel reached consensus after discussion, using four prespecified criteria:

**Severity:** the marker's ability to indicate ARI severity.
**Specificity:** the plausibility of linking the marker unambiguously to the ARI episode under surveillance.
**Relevance:** whether primary care clinicians would routinely use it to characterise an ARI episode.
**Recording:** the likelihood that the marker is captured in structured CMR data, via the clinical terminology SNOMED CT.

### 2.10 Ethics Statement

No ethics approval was required for this study as it was completed solely on publicly available data. This was confirmed by use of the Health Research Authority ethics decision tool [18].

## 3 Results

### 3.1 Study Selection

**FIGURE 1:**
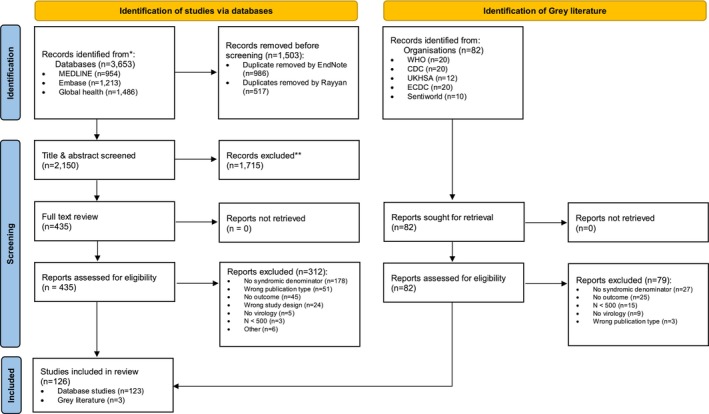
Modified from: Page MJ, McKenzie JE, Bossuyt PM, Boutron I, Hoffmann TC, Mulrow CD, et al. The PRISMA 2020 statement: an updated guideline for reporting systematic reviews. BMJ 2021;372:n71. doi: 10.1136/bmj.n71.

Following deduplication, 2150 studies remained from the database search and 82 from the grey literature search (Figure 1). Following title and abstract screening, 435 database articles were put forward for full‐text review, while all 82 grey literature items were advanced directly. After full‐text assessment, 123 database studies and 3 grey literature studies met the inclusion criteria, yielding a total of 126 studies in the systematic review. Studies were most commonly excluded due to a lack of patient recruitment for any of the specified respiratory clinical syndromes: ILI, ARI, SARI or suspected COVID‐19 (53%, 205/391).

### 3.2 Study Characteristics

The nature of the 126 studies was varied and covered a range of ARI‐related topics focusing on surveillance, epidemiology, clinical presentation, severity, treatment, outcomes and disease burden. The characteristics of each study can be seen in Table 1 with a full overview in Appendix 6.

**TABLE 1:** Summary characteristics of the 126 studies included in the systematic review.

Characteristic	Category/statistic	Value
Publication timeline	Median (range)	Jun 2018 (Oct 2009–Jul 2023)
Study start	Median (range)	Oct 2010 (Jan 1993–May 2023)
Study duration	Median (range, days)	763 (7–6208)
Document type	Peer‐reviewed articles	118 (94%)
	Surveillance reports	8 (6%)
Geographic scope	Multinational	10 (8%)
	National/subnational	116 (92%)
World region (255 appearances)	Europe	84 (33%)
	Asia	65 (25%)
	North America	37 (15%)
	Africa	37 (15%)
	South America	25 (10%)
	Oceania	7 (3%)
Income band^a^ (255 appearances)	High	117 (46%)
	Upper–middle	75 (29%)
	Lower–middle	48 (19%)
	Low	14 (5%)
Case types (139 instances) ^b^	SARI	47 (34%)
	ILI	42 (30%)
	ARI	23 (17%)
	Suspected COVID‐19	14 (10%)
	MAARI	1 (1%)
	Severe pneumonia	1 (1%)
	Combined	11 (8%)
Recruitment setting (139) ^b^	Hospitalised	72 (52%)
	Treatment‐seeking^c^	34 (24%)
	Mixed	15 (11%)
	Non treatment‐seeking^d^	8 (6%)
	ICU	4 (3%)
	Unknown	6 (4%)

*Note:* Percentages for world region total = 255 as multinational studies contribute > 1 country.

Abbreviations: ARI = Acute Respiratory Infection; ICU = Intensive Care Unit; ILI = Influenza‐like Illness; MAARI = Medically Attended ARI; SARI = Severe Acute Respiratory Infection.

^a^ Based on World Bank figures from 2024. One country Venezuela remained unclassified in 2024 World Bank data [19].

^b^ Thirteen studies reported outcomes in more than 1 case type hence 139 reporting instances in the 126 studies.

^c^ Treatment‐seeking populations included those attending primary care, emergency department and outpatients.

^d^ Non treatment‐seeking populations included those identified through community surveys.

Most records were peer‐reviewed journal articles (118/126, 94%), with peaks in publication around the 2009 H1N1 and 2020 SARS‐CoV‐2 pandemic years (Appendix 7). Of the included studies, 10 (8%) were multinational and the remaining 116 (92%) were national or subnational. Data came from 84 countries, most commonly in high‐income settings (117/255 appearances, 46%). Europe was the most commonly represented region (84/255, 33%); however, the top 3 most represented countries were Mexico (17 studies, 13%), the United States (13, 10%) and Brazil (11, 9%).

Case labels, such as ILI, ARI and SARI, were taken as used by the authors. We did not relabel cases whose definitions diverged from convention (e.g., ‘ILI’ in hospitalised patients). To maintain clarity, each study's recruitment setting is reported alongside its case type. Thirteen papers reported outcomes for two different syndromes (13/126, 10%), yielding 139 reporting instances overall; severe acute respiratory infection (SARI) (47/139, 34%) and influenza‐like illness (ILI) (42/139, 30%) were the most common. The recruitment setting was predominantly hospital‐based (72/139, 52%), followed by treatment‐seeking populations (34/139, 24%). In addition to the case types eligible for inclusion, one study reported outcomes in medically attended acute respiratory infection (MAARI) and another in severe pneumonia. The definitions of these were thought to meet the inclusion criteria as they represented cases very similar to ILI and SARI.

### 3.3 Severity Markers

The systematic review identified 11 groups of candidate severity markers: 4 groups of severe outcomes and 7 groups of potential predictors of severe outcomes. In total, there were 77 distinct candidate severity markers (Table 2). Twenty‐one were classified as severe outcomes: 7 complications (e.g., sepsis, organ failure), 7 hospital‐related events, 6 ICU‐related events and death. The remaining 56 were considered potential predictors of severe outcomes. These predictors comprised 20 presenting symptoms, 8 clinical signs, 14 severity scores (e.g., SOFA, NEWS, WHO scale), 8 investigations, 3 treatments, 2 absenteeism measures and 1 indicator of treatment‐seeking behaviour.

**TABLE 2:** Included severity markers identified in the systematic review. Categorised as severe outcomes and potential predictors of severe outcomes.

Outcome	Description	Rationale for inclusions and notes
Severe outcomes		
Complications		
Respiratory complications (18/139, 13%) [20, 21, 22, 23, 24, 25, 26, 27, 28, 29, 30, 31, 32, 33, 34]	Any acute complication of the respiratory system including respiratory failure and ARDS.	Severe outcome. ARI‐specific (esp. respiratory failure). Recorded in CMR via hospital discharge summaries, so not timely.
Sepsis (4/139, 3%) [33, 35, 36, 37]	Severe systemic infection including septic shock and systemic inflammatory response syndrome (SIRS).	Severe outcome. Infection‐related although not ARI‐specific. Recorded in CMR via hospital discharge summaries, so not timely.
Hospital		
Hospital admission (27/139, 19%) [22, 23, 30, 33, 37, 38, 39, 40, 41, 42, 43, 44, 45, 46, 47, 48, 49, 50, 51, 52, 53, 54, 55]	Emergency hospital admission for an ARI.	Standard epidemiological severe outcome. In primary care CMRs, both an admission and a diagnosis (e.g., pneumonia) may be recorded, but they are not directly linked, making it difficult to define an ARI‐specific admission. Admissions are captured via hospital discharge summaries, so reporting is not timely.
Hospital attendance (2/139, 1%) [51, 53]	Emergency hospital attendance for an ARI.	Standard epidemiological severe outcome. In primary care CMRs, both an attendance and a diagnosis (e.g., pneumonia) may be recorded, but they are not directly linked, so it is difficult to define an ARI‐specific attendance. Attendances are captured via emergency department discharge summaries, so reporting is not timely.
Hospital attendance advised (6/139, 4%) [23, 37, 48, 56, 57, 58]	Emergency hospital attendance for an ARI advised.	Indicates higher concern by the clinician, as emergency attendance is recommended. More easily attributable to an ARI since the advice is usually recorded at the same time as the ARI consultation in the primary care CMR, so more timely than outcomes captured via hospital discharge summaries. However, such advice is less consistently recorded compared with admissions or attendances.
Intensive care		
Intensive care unit Admission (ICU admission) (62/139, 45%) [20, 21, 23, 25, 26, 28, 29, 30, 31, 32, 33, 34, 37, 38, 39, 40, 42, 44, 45, 46, 48, 49, 50, 51, 52, 54, 55, 56, 57, 59, 60, 61, 62, 63, 64, 65, 66, 67, 68, 69, 70, 71, 72, 73, 74, 75, 76, 77, 78, 79, 80, 81, 82, 83, 84, 85, 86, 87, 88, 89]	Emergency admission to the intensive care unit (ICU) for ARI.	Standard epidemiological severe outcome. In primary care CMRs, admissions and diagnoses may both be recorded, but they are not easily linked, making ARI‐specific attribution difficult. Captured via hospital discharge summaries, so reporting is not timely.
Death		
Death (100/139, 72%) [20, 21, 22, 23, 24, 25, 26, 27, 28, 29, 30, 31, 32, 33, 35, 37, 38, 39, 40, 41, 43, 45, 46, 48, 49, 50, 51, 52, 54, 55, 56, 59, 60, 61, 62, 63, 64, 65, 66, 67, 68, 69, 70, 71, 72, 73, 74, 75, 76, 79, 80, 81, 82, 83, 84, 85, 86, 87, 88, 89, 90, 91, 92, 93, 94, 95, 96, 97, 98, 99, 100, 101, 102, 103, 104, 105, 106, 107, 108, 109, 110, 111, 112, 113, 114, 115, 116, 117, 118, 119, 120, 121, 122, 123, 124, 125, 126]	—	Standard epidemiological severe outcome. Recorded in primary care CMRs, but attribution to ARI is uncertain as cause of death is not systematically coded. Deaths may be recorded more promptly than hospitalisations, since automatic systems exist in the NHS to capture deaths across all healthcare settings.
Predictors of severe outcomes		
symptom		
Haemoptysis (7/139, 5%) [25, 27, 87, 96, 102]	Coughing up blood	May indicate more severe respiratory infection due to airway or lung tissue damage and associated inflammation. If recorded, it is usually done at the time of the ARI consultation, making it specific and timely.
Dyspnoea (42/139, 30%) [20, 21, 22, 23, 24, 25, 26, 27, 29, 31, 33, 42, 48, 53, 54, 56, 58, 59, 68, 77, 79, 86, 87, 93, 97, 99, 102, 107, 110, 112, 116, 119, 122, 124, 127, 128]	Shortness of breath	May indicate compromise of the respiratory system and hypoxia, making it a marker of more severe infection. If recorded, it is usually done at the time of the ARI consultation, making it specific and timely.
Fever (41/139, 29%) [20, 21, 22, 24, 26, 27, 29, 33, 35, 36, 41, 42, 46, 47, 51, 53, 54, 56, 59, 63, 68, 77, 79, 86, 93, 95, 96, 99, 100, 102, 110, 112, 115, 124, 127, 128, 129]	A subjective report of a high body temperature	May indicate a degree of systemic upset and a more widespread response to infection. If recorded, it is usually captured at the time of the ARI consultation, making it specific and timely. Less value as a marker in paediatric patients (where fever is very common) and in older adults (who may not mount a febrile response).
Malaise and loss of appetite (23/139, 17%) [20, 24, 26, 29, 33, 42, 51, 56, 68, 77, 93, 95, 99, 102, 110, 112, 115, 119, 122, 127, 130]	Feeling generally unwell	May indicate systemic upset and more severe illness. If recorded, it is usually captured at the time of the ARI consultation, making it timely and specific.
Confusion (3/139, 2%) [48, 56, 112]	Altered mental status or disorientation	May indicate systemic upset, especially in very young or older patients. Often linked with hypoxia, sepsis or shock. If recorded, it is usually captured at the time of the ARI consultation, making it timely and specific.
Treatment‐seeking behaviour		
Treatment‐seeking behaviour (3/139, 2%) [47, 129, 131]	A patient seeks a consultation from a healthcare professional.	Includes ambulance encounters or contact with NHS 111 (a free UK advice line). Seeking urgent or unscheduled care may indicate the patient or clinician perceived the illness as more severe. Likely recorded in primary care CMRs. Not ARI‐specific, but included on balance.
Absence		
Work absence (7/139, 5%) [47, 129, 130, 132, 133, 134]	Time taken off from work due to illness or medical reasons.	Very likely recorded in primary care via electronic fit notes (Med3). Longer absences (e.g., > 2 weeks) may reflect greater severity of illness. However, the reason for absence is not always identifiable, and this is only relevant for working‐age adults.
Clinical signs		
Body temperature (17/139, 12%) [23, 35, 36, 46, 47, 54, 57, 65, 68, 82, 87, 97, 107, 116, 119, 135]	An objective measure of core body temperature.	Fever or abnormal temperature is a recognised marker of systemic upset and can indicate systemic involvement. Likely to be recorded at the time of the ARI event in primary care, therefore timely.
Pulse rate (6/139, 4%) [38, 48, 57, 65, 102]	An objective measure of pulse rate.	Tachycardia or bradycardia is a recognised marker of physiological stress and systemic upset. Likely to be recorded at the time of the ARI event in primary care, therefore timely.
Respiratory rate (14/139, 10%) [23, 25, 35, 38, 42, 48, 53, 54, 58, 65, 82, 87, 110, 135]	An objective measure of respiratory rate.	An abnormal respiratory rate is a recognised marker of systemic upset and respiratory compromise. Likely to be recorded at the time of the ARI event in primary care, therefore timely.
Oxygen saturation (O_2_ sat) (15/139, 11%) [22, 35, 38, 48, 53, 54, 58, 82, 102, 118, 119, 122, 135]	An objective estimate of blood oxygenation.	Oxygen saturation is an indirect measure of hypoxia and a key marker of respiratory compromise and systemic upset. Likely to be recorded at the time of the ARI event in primary care, therefore timely.
Blood pressure (BP) (4/139, 3%) [27, 38, 48]	An objective measure of blood pressure.	Blood pressure can indicate circulatory compromise in severe ARI. Likely recorded at the time of the ARI event in primary care, therefore timely, but not commonly recorded in children.
Work of breathing (7/139, 5%) [51, 102, 119, 122, 135]	For example, use of accessory muscles or grunting. Often referred to as respiratory distress in a child.	Helps in the assessment of severity as increased effort (e.g., use of accessory muscles, grunting) indicates respiratory distress and possible hypoxia. Likely recorded at the time of the ARI event in primary care, therefore timely.
Chest examination findings (13/139, 9%) [23, 25, 31, 42, 48, 54, 57, 58, 59, 87, 100]	Presence of wheeze or crackles on clinical examination.	Findings such as wheeze or crackles may indicate lower respiratory tract involvement and possible complications like pneumonia, indicating greater severity. Likely recorded at time of the ARI consultation in primary care, and therefore timely.
Cyanosis (4/139, 3%) [48, 51, 99, 110]	Blue discoloration to skin or mucus membranes caused by hypoxia.	Indicates significant hypoxia and is therefore a marker of severe disease. Although relatively uncommon in primary care, if present it is likely to be recorded at the time of the ARI consultation, making it timely.
Clinical scores		
Modified early warning Score (MEWS) (1/139, 1%) [136]	Acute illness score: a tool used in hospitals to detect early deterioration in patients by monitoring vital signs. Includes a range of related scores MEWS, NEWS, NEWS2, PEWS.	Captures severity through changes in multiple vital signs and is widely used in hospitals. In the NHS, National Early Warning Score 2 (NEWS2) has replaced MEWS as the standard tool for detecting critical illness. Less commonly recorded in primary care, but included due to its relevance as a cross‐sector severity measure.
Glasgow coma scale (GCS) (1/139, 1%) [118]	Acute consciousness: A neurological scale used to assess consciousness level.	A neurological scale assessing level of consciousness. Severe reductions in GCS reflect significant systemic or neurological compromise and therefore serve as a strong indicator of severity. Commonly used across the NHS, though less frequently recorded in primary care. Included due to its clear role as a severity marker.
Investigation		
White blood cell count (11/139, 8%) [22, 23, 37, 48, 51, 53, 54, 56, 57, 58, 110]	Total circulating leukocytes (with differential).	Elevated or abnormal WBC can indicate systemic infection or more severe inflammatory response. If undertaken in primary care, results are recorded in CMRs, but often become available some time after the ARI consultation, reducing timeliness and specificity.
Inflammatory markers (5/139, 4%) [22, 23, 48, 53, 56]	Acute phase reactants such as C‐reactive protein (CRP) or erythrocyte sedimentation rate (ESR). Often elevated during infections, typically in bacterial infections.	Elevated inflammatory markers indicate systemic infection or more severe inflammatory response. If undertaken in primary care, results are recorded in CMRs, but often become available some time after the ARI consultation, reducing timeliness and specificity.
Chest X‐ray (9/139, 6%) [31, 44, 46, 51, 54, 57, 58, 87]	May identify consolidation or lung parenchymal changes associated with respiratory disease.	Findings such as consolidation or parenchymal changes can indicate more severe respiratory disease (e.g., pneumonia). If requested in primary care, results are recorded in CMRs, though they are usually available after the ARI consultation, limiting timeliness and specificity.
Treatments		
Antibiotics (19/139, 14%) [23, 27, 35, 36, 37, 45, 47, 48, 52, 53, 54, 57, 58, 65, 74, 85, 124, 137]	Used to treat suspected bacterial infections including: bacterial throat infections and pneumonia.	Prescription of antibiotics often reflects increased clinical concern and may indicate more severe infection. Very well recorded in primary care CMRs and available in a timely manner. Common respiratory antibiotics include amoxicillin, penicillin V, macrolides, doxycycline, co‐amoxiclav and cephalosporins.
Antivirals (21/139, 15%) [20, 23, 30, 37, 47, 54, 55, 63, 64, 67, 79, 95, 96, 99, 107, 110, 125, 130, 138]	Used to treat viral infections including influenza (e.g., oseltamivir) or SARS‐CoV‐2 infection (e.g., nirmatrelvir + ritonavir)	Prescription of antivirals may indicate increased severity, depending on the specific drug. Generally well recorded in primary care CMRs and available in a timely manner. SARS‐CoV‐2 antivirals were excluded as they are rarely prescribed in primary care.
Steroids (1/139, 1%) [82]	Used to treat a number of acute respiratory conditions. For example, exacerbations of chronic lung disease, croup and severe SARS‐CoV‐2 infection.	Oral steroids are commonly prescribed in primary care for certain ARIs (e.g., exacerbations of asthma or COPD and croup). Their use may indicate greater severity. They are highly likely to be recorded in CMRs in a timely manner.

**FIGURE 2:**
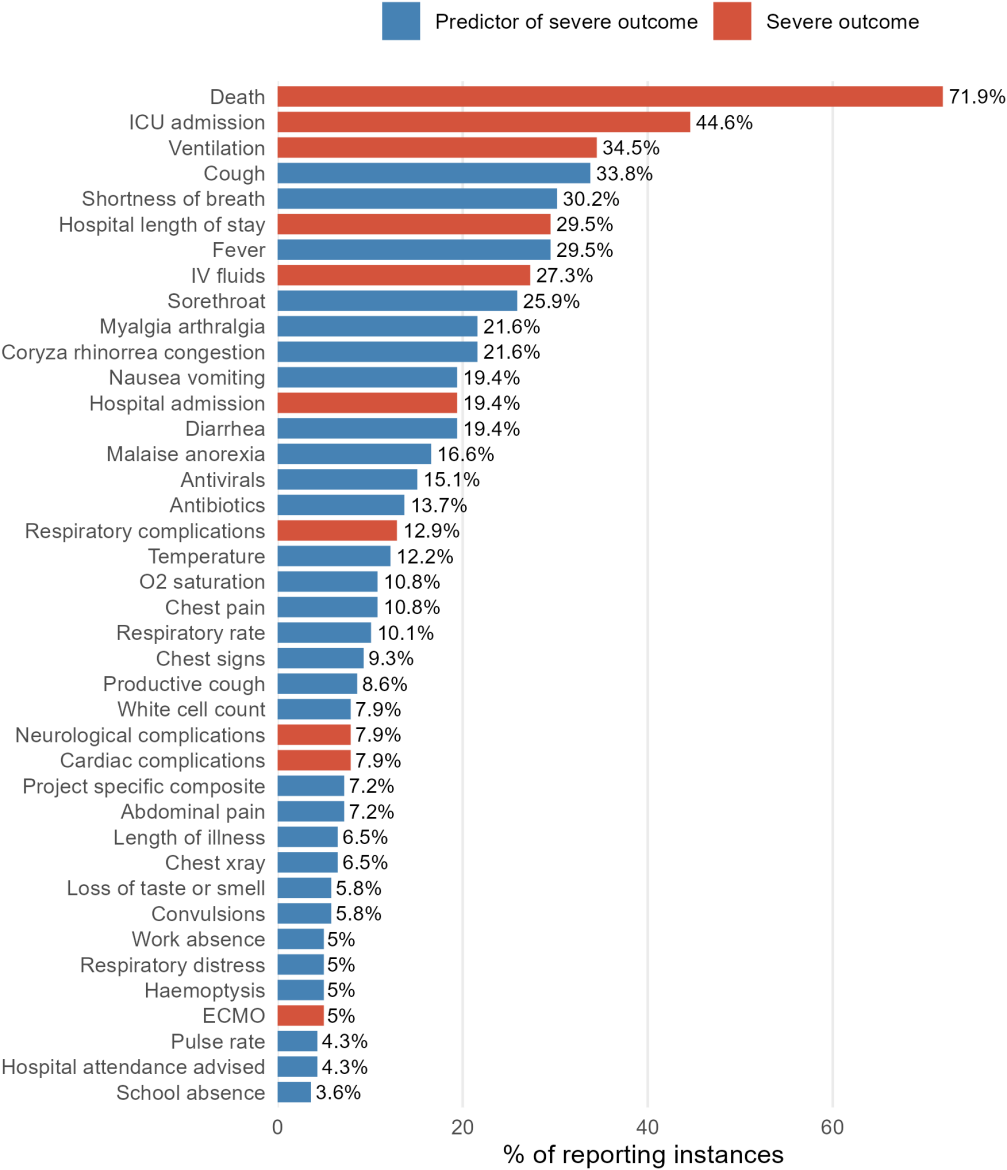
Frequency of reporting of severity markers. Horizontal bars show the top 40 most frequently reported severity markers from the systematic review, ordered by the percentage of studies reporting each marker. Bars are coloured by classification: Severe outcome (red) versus Predictor of severe outcome (blue). Values on bars are exact percentages (% of included studies that reported the marker). ICU: Intensive care unit, IV: Intravenous, ECMO: Extracorporeal membrane oxygenation. A breakdown in reporting frequency can be seen in Appendix 8.

The three most frequently reported outcome *groups* overall were death (100/139 reporting instances, 72%), hospital‐related outcomes (73/139, 53%) and ICU‐related outcomes (65/139, 47%). Outcome reporting frequencies varied by recruitment type (Appendix 8). The most commonly reported severity markers were death (72%), ICU admission (45%), ventilation (35%), cough (34%), shortness of breath (30%) and hospital length of stay (30%) (Figure 2).

### 3.4 Primary Care Informatics Review

Following the primary care informatics review, 47 of the original 77 candidate markers were deemed unlikely to add value for CMR‐based ARI surveillance in general practice. Table 2 presents the final 30 included severity markers by group. Appendix 9 sets out the fully referenced 77 candidate severity markers from the systematic review and the reasons for excluding these 47 and retaining the 30. Of the 30 markers, 7 (from hospital, complication, ICU or death groups) were regarded as severe outcomes, and the remaining 23 (from symptoms, treatment‐seeking behaviour, absenteeism, clinical signs, clinical scores, investigations and treatments groups) were regarded as potential predictors of severe outcomes.

## 4 Discussion

This systematic review identified a list of candidate severity markers for ARI. The review evaluated 126 studies from 84 countries across various healthcare settings. The primary care informatics review subsequently refined this list in order to select the most suitable severity markers in the context of primary care CMR‐based ARI surveillance in England. The review ultimately identified 30 severity markers that could be used to develop severity indicators at RSC.

The additional expert review was conducted by practising primary‐care clinicians with expertise in informatics and ARI surveillance. The aim was to identify severity markers specifically suited to CMR‐based primary care ARI surveillance. Others should draw on experts from their own field to tailor the list of severity markers to their specific context. For example, those developing indicators for hospital or ICU surveillance may find greater value in markers specific to those settings, such as the use of oxygen therapy or ECMO.

Use of severity pyramids can help individuals consider appropriate severity indicators for a given setting [132]. At the base of these pyramids are individuals infected in the community, with each level representing increasing severity, such as attending primary care with an ARI, visiting the emergency department, hospital admission, ICU admission and death at the top. In community‐based questionnaire surveillance, any outcome higher in the pyramid may be relevant. In contrast, for ICU patients with ARI, only outcomes within the ICU, such as ventilation duration or death, would be appropriate severity markers.

Of the 30 final severity markers, 7 were classed as severe outcomes in their own right, while the remaining 23 were treated as potential predictors of severity. These 7 outcomes correspond to the traditional adverse endpoints used in respiratory infection surveillance. Severe outcomes typically occur late in the clinical course of ARIs and are often entered into the primary care record only after a hospital discharge summary is received, delaying their appearance in the CMR. Consequently, these outcomes are of less value to prospective or near‐real‐time surveillance, but they remain useful for end‐of‐year reporting and for vaccine‐effectiveness studies, where timeliness is less critical. Predictors, on the other hand, are more likely to be recorded contemporaneously in the CMR. For example, shortness of breath or pulse rate is very commonly recorded in association with an episode of ARI at the time of the diagnosis. These could therefore support more timely surveillance reporting of ARI severity for use in prospective surveillance.

### 4.1 Strengths and Limitations

This systematic review examined a large volume of evidence, which included relevant grey literature and the output from reproducible bibliographic database searches. Full texts were reviewed independently by two reviewers. In addition, four practising primary care clinicians active in the field of informatics and CMR‐based surveillance performed the final stage of the review. The primary care informatics review was added to increase the contextual relevance of the severity markers.

Although we followed established systematic review methods, the search strategy and screening process may not have identified all relevant studies. In addition, there was inherent subjectivity during screening and full‐text review in deciding what counted as a candidate severity marker, which may have influenced which studies were ultimately included. To minimise this, we asked reviewers to apply the question: ‘On balance, might the presence (or absence) of this characteristic in an individual with ARI predict, or itself constitute, a severe outcome?’ Even so, some subjectivity inevitably remained. This limitation, however, does not undermine our findings because the purpose of the study was to compile a shortlist for further evaluation, not to deliver a fully validated set of severity markers.

A formal risk‐of‐bias appraisal was not undertaken because this review was designed as a descriptive ‘evidence‐mapping’ exercise: its goal was to catalogue candidate severity markers, not to quantify effect sizes or compare outcomes across studies. We therefore did not need to weight findings by study quality, pool estimates or draw causal inferences.

### 4.2 Public Health Implications

This review provides a first step towards extending the RSC surveillance platform so that it reports not only the incidence of ARI but also its severity. The identified severity markers could support the development of two complementary indicator types: case‐based measures, for example the case‐hospitalisation or case‐fatality ratio (CHR/CFR), which express the chance that a detected case becomes severe, and population‐based rates, which capture the incidence of severe infection in the wider community.

Because the selected markers can be extracted directly from primary care records, without the need for linkage to hospital or mortality datasets, the technical and governance barriers to timely severity reporting are substantially lower. Identifying potential predictors of severe outcomes opens the door to further development of even more timely severity markers that could help forecast pressure on health services. Examining severity by age, location and ethnicity, for example, could pinpoint groups at higher risk, thereby helping to address health inequalities.

Even so, additional research is required. Firstly, the data quality, including the completeness of recording, of each severity marker in primary care CMR data must be quantified. Secondly, the 23 candidate predictors need formal validation to test whether they are associated with or predict the severe outcomes identified. Work is already under way to (i) audit data quality for all 30 markers and (ii) evaluate the performance of the candidate indicators in a large RSC cohort. The findings from those studies will provide the bridge to implementing severity markers in practice. Once validated, the new measures can be incorporated into the RSC's weekly and annual surveillance reports, giving UKHSA more detailed information to guide public health action during both routine respiratory virus seasons and future pandemics.

Markers of severity are needed not only for surveillance but are also of value in observational studies and clinical trials. This work also provides individuals looking to identify cases of severe disease for these studies with a range of possible options.

## 5 Conclusions

Markers of ARI severity are essential for surveillance, as incidence alone cannot fully capture the potential impact of circulating respiratory viruses on society. There is an urgent need to develop systems that measure population‐level severity and do so in a timely way. This work provides a stepping stone towards the development and implementation of severity indicators within a large, CMR‐based primary care ARI surveillance system. In doing so, we aim to maximise the value of routinely collected primary care data, strengthen day‐to‐day respiratory surveillance, so the system is better equipped to support rapid risk assessment in any future pandemic.

## Author Contributions

**William H. Elson:** conceptualization, methodology, data curation, formal analysis, visualization, writing – review and editing, writing – original draft, investigation. **Anna Forbes:** conceptualization, methodology, writing – review and editing, investigation, writing – original draft, data curation. **Rashmi Wimalaratna:** investigation, writing – review and editing. **Roger Morbey:** investigation, writing – review and editing, formal analysis, validation, supervision. **F. D. Richard Hobbs:** conceptualization, writing – review and editing, validation, supervision. **Simon de Lusignan:** conceptualization, writing – review and editing, validation, supervision.

## Ethics Statement

Ethics approval was not required for this study as it was based solely on publicly available data. This was confirmed using the UK Health Research Authority ethics decision tool. Patient consent was not required as the study involved no direct contact with patients and used only publicly available data.

## Consent

This manuscript does not reproduce any material from other sources that requires permission. Artificial intelligence (AI) was used in a limited way only to assist with spelling and grammar checking. No text was generated de novo using AI tools. All content was drafted and developed by the authors. The final manuscript has been fully reviewed by all authors, who take full responsibility for its accuracy and integrity.

## Conflicts of Interest

Authors disclose the following conflicts of interest: S.L. has received institutional funding for vaccine‐related research from AstraZeneca, G.S.K., Moderna, Pfizer, Sanofi and Seqirus. He has participated in advisory boards (with funding to his university) for G.S.K., Sanofi and Seqirus, and has received funding for conference travel or speaking engagements from AstraZeneca and Moderna. A.F. has received funding for conference travel and fees from AstraZeneca in 2024. The remaining authors declare no conflicts of interest.

## Peer Review

The peer review history for this article is available at https://www.webofscience.com/api/gateway/wos/peer‐review/10.1111/irv.70172.

## Supporting information


**Data S1:** Supporting information.

**Data S2:** Supporting information.

**Data S3:** Supporting information.

## Data Availability

All data used in this review were extracted from published studies and publicly available sources. Extracted data from included studies is available in the Supporting Information S2: appendix.
