# Supplementary material for: A Systematic Review of the Markers of Severity in Acute Respiratory Infections to Inform Primary Care Surveillance
Source: Influenza Other Respir Viruses. 2025 Oct 24;19(10):e70172. doi: 10.1111/irv.70172 (PMC12550406; doi:10.1111/irv.70172)
Supplement: Supplementary file 1 — Data S1: Supporting information. [file IRV-19-e70172-s001.docx]

Appendix 6: R-script of raw data used in the systematic review. Please follow the instructions included in the script to output a csv file of raw data

###############################################################

# Data appendix for:

# "A Systematic Review of the Markers of Severity in Acute

# Respiratory Infections to Inform Primary Care Surveillance"

#

# Authors:

# William H. Elson1*, Anna Forbes1, Gavin Jamie1, Rashmi Wimalaratna1,

# Roger Morbey2, FD Richard Hobbs1,3, Simon de Lusignan1,

# Jamie Lopez Bernal4

#

# This file contains the R object `data` used in the manuscript,

# stored in plain text using `dput()`.

#

# To recreate the dataset in your R environment:

# 1. Save this file as "appendix_data.R" or copy the full text into an R file

# 2. Run the script to output csv file to working directory

#

# The object `data` will then be available for analysis.

###############################################################

data <- {structure(list(DETAILS_study_id = c("S001", "S001", "S002", "S003",

"S004", "S005", "S006", "S007", "S008", "S009", "S010", "S011",

"S012", "S013", "S014", "S015", "S016", "S017", "S018", "S019",

"S020", "S021", "S022", "S023", "S024", "S025", "S026", "S027",

"S028", "S029", "S029", "S030", "S031", "S031", "S032", "S033",

"S034", "S035", "S035", "S036", "S036", "S037", "S038", "S039",

"S040", "S041", "S042", "S043", "S044", "S044", "S045", "S046",

"S047", "S047", "S048", "S049", "S050", "S051", "S052", "S052",

"S053", "S054", "S055", "S056", "S057", "S058", "S059", "S060",

"S061", "S062", "S063", "S064", "S065", "S066", "S067", "S068",

"S069", "S070", "S071", "S072", "S073", "S074", "S075", "S076",

"S077", "S078", "S079", "S080", "S081", "S082", "S082", "S083",

"S083", "S084", "S085", "S086", "S087", "S088", "S089", "S090",

"S091", "S092", "S093", "S093", "S094", "S094", "S095", "S096",

"S097", "S098", "S099", "S100", "S101", "S102", "S103", "S104",

"S105", "S106", "S107", "S108", "S109", "S110", "S111", "S112",

"S113", "S114", "S115", "S116", "S116", "S117", "S118", "S119",

"S120", "S121", "S122", "S123", "S124", "S125", "S126"), DETAILS_study_title = c("A Comparison of the Epidemiology and Clinical Presentation of Seasonal Influenza A and 2009 Pandemic Influenza A (H1N1) in Guatemala",

"A Comparison of the Epidemiology and Clinical Presentation of Seasonal Influenza A and 2009 Pandemic Influenza A (H1N1) in Guatemala",

"A Comprehensive Descriptive Epidemiological and ClinicalAnalysis of SARS-CoV-2 in West-Mexico during COVID-19Pandemic 2020",

"A clinico-epidemiological profile, coinfections and outcome of patients with Influenza Like Illnesses (ILI) presenting to the emergency department during the COVID-19 pandemic\n",

"A cross-sectional analysis of symptom severity in adults with influenza and other acute respiratory illness in the outpatient setting.",

"A review of the dynamics and severity of the pandemic A(H1N1) influenza virus on R\x82union island, 2009",

"Analysis of Risk Factors for Severe Acute Respiratory Infection and Pneumonia and among Adult Patients with Acute Respiratory Illness during 2011-2014 Influenza Seasons in Korea",

"Antibiotic Use in Suspected and Confirmed COVID-19 Patients Admitted to Health Facilities in Sierra Leone in 2020?2021: Practice Does Not Follow Policy",

"Associations between chronic conditions and death in hospital among adults (aged 20+ years) during first acute care hospitalizations with a confirmed or suspected COVID-19 diagnosis in Canada",

"Building influenza surveillance pyramids in near real time, Australia\n",

"CDC-Respiratory Virus Surveillance Among Children with Acute Respiratory Illnesses ? New Vaccine Surveillance Network, United States, 2016?2021",

"COVID-19 and Severe Acute Respiratory Infections: Monitoring Trends in 421 German Hospitals During the First Four Pandemic Waves",

"Changes in Surveillance of Acute Respiratory Infections Including Influenza in Slovak Republic during 1993-2008",

"Characterisation of acute respiratory infections at a United Kingdom paediatric teaching hospital: observational study assessing the impact of influenza A (2009 pdmH1N1) on predominant viral pathogens\n",

"Characterizing the Epidemiology of the 2009 Influenza A/H1N1 Pandemic in Mexico",

"Chronic use of inhaled corticosteroids in patients admitted for respiratory virus infections: a 6-year prospective multicenter study\n",

"Clinical Profile of COVID-19 Illness in Children?Experience from a Tertiary Care Hospital",

"Clinical and epidemiological aspects of severe acute respiratory infection: before and during the first year of the COVID-19 pandemic in Brazil",

"Clinical and phylogenetic influenza dynamics for the 2019-20 season in the global influenza hospital surveillance network (GIHSN) - Pilot study",

"Clinical characteristics and factors associated with hospital admission or death in 43 103 adult outpatients with coronavirus disease 2019 managed with the Covidom telesurveillance solution: a prospective cohort study",

"Clinical characteristics and factors associated with severe acute respiratory infection and influenza among children in Jingzhou, China",

"Clinical characteristics and outcome of respiratory syncytial virus infection among adults hospitalized with influenza-like illness in France",

"Clinical characteristics and outcomes of influenza and other influenza-like illnesses in Mexico City",

"Clinical characteristics and outcomes of patients with severe acute respiratory infections (SARI): results from the Egyptian surveillance study 2010?2014",

"Clinical features and mortality in COVID-19 SARI versus non COVID-19 SARI cases from Western Rajasthan, India",

"Comparative epidemiology of five waves of COVID-19 in Mexico, March 2020?August 2022",

"Comparison of common acute respiratory infection case definitions for identification of hospitalized influenza cases at a population-based surveillance site in Egypt",

"Coronavirus Surveillance in a Pediatric Population in Jordan From 2010 to 2013: A Prospective Viral Surveillance Study",

"Deaths and hospitalizations related to 2009 pandemic influenza a (H1N1) - Greece, May 2009-February 2010",

"Description of Hospitalized Cases of Influenza A(H1N1)pdm09 Infection on theBasis of the National Hospitalized-Case Surveillance, 2009?2010, Japan",

"Description of Hospitalized Cases of Influenza A(H1N1)pdm09 Infection on theBasis of the National Hospitalized-Case Surveillance, 2009?2010, Japan",

"Descriptive Epidemiology of Novel Influenza A (H1N1), Andhra Pradesh 2009-2010",

"Detection of Influenza C Viruses Among Outpatients and Patients Hospitalized for Severe Acute Respiratory Infection, Minnesota, 2013?2016",

"Detection of Influenza C Viruses Among Outpatients and Patients Hospitalized for Severe Acute Respiratory Infection, Minnesota, 2013?2016",

"Detection of Viral and Bacterial Pathogens in Hospitalized Children With Acute Respiratory Illnesses, Chongqing, 2009?2013",

"Digitalizing and upgrading severe acute respiratory infections surveillance in Malta: system development\n",

"Disparities Between Black and White Children in Hospitalizations Associated With Acute Respiratory Illness and Laboratory-confirmed Influenza and Respiratory Syncytial Virus in 3 US Counties?2002?2009",

"Effects of seasonal and pandemic influenza on health-related quality of life, work and school absence in England: Results from the Flu Watch cohort study",

"Effects of seasonal and pandemic influenza on health-related quality of life, work and school absence in England: Results from the Flu Watch cohort study",

"Epidemiologic and spatiotemporal characterization of influenza and severe acute respiratory infection in Uganda, 2010-2015",

"Epidemiologic and spatiotemporal characterization of influenza and severe acute respiratory infection in Uganda, 2010-2015",

"Epidemiologic profile of severe acute respiratory infection in Brazil during the COVID-19 pandemic: an epidemiological study\n",

"Epidemiological Characteristics and Underlying Risk Factors for Mortality during the Autumn 2009 Pandemic Wave in Mexico",

"Epidemiological Characterization of a Fourth Wave of Pandemic A/H1N1 Influenza in Mexico, Winter 2011?2012: Age Shift and Severity",

"Epidemiological Trends of Coronavirus Disease 2019 in Sierra Leone From March 2020 to October 2021\n",

"Epidemiological and clinical profile of Influenza A(H1N1) pdm09 in Odisha, eastern India",

"Epidemiology and outcome of influenza-associated infections\namong hospitalized patients with acute respiratory infections,\nEgypt national surveillance system, 2016-2019",

"Epidemiology and virology of acute respiratory infections during the first year of life: a birth cohort study in Vietnam\n",

"Epidemiology of Influenza in Fars Province, Southern Iran; a Population-Based Study (2015-2019)\n",

"Epidemiology of Influenza in Fars Province, Southern Iran; a Population-Based Study (2015-2019)\n",

"Epidemiology of Severe Acute Respiratory Illness and Risk Factors for Influenza Infection and Clinical Severity among Adults in Malawi, 2011?2013",

"Epidemiology of hospital admissions with influenza during the 2013/2014 Northern Hemisphere influenza season: results from the Global Influenza Hospital Surveillance Network\n",

"Epidemiology of influenza B infection in the state of Rio Grande do Sul, Brazil, from 2003 to 2019",

"Epidemiology of influenza B infection in the state of Rio Grande do Sul, Brazil, from 2003 to 2019",

"Epidemiology of severe acute respiratory illness (SARI) among adults and children aged 5 years in a high HIV-prevalence setting, 2009-2012",

"Epidemiology, disease severity and outcome of Severe acute respiratory syndrome coronavirus 2 and influenza viruses coinfection seen at Egypt integrated acute respiratory infections surveillance, 2020-2022",

"Establishing a sentinel surveillance system for the novel COVID-19 in a resource-limited country: methods, system attributes and early findings",

"Establishing an ICD-10 code based SARI-surveillance in Germany - description of the system and first results from five recent influenza seasons",

"Establishing thresholds and parameters for pandemic influenza severity assessment, Australia\n",

"Establishing thresholds and parameters for pandemic influenza severity assessment, Australia\n",

"Estimating age-specific influenza-related hospitalization rates during the pandemic (H1N1) 2009 in Davidson Co, TN",

"Estimating severity of influenza epidemics from severe acute respiratory infections (SARI) in intensive care units\n",

"Estimating the burden of influenza-associated hospitalization and deaths in Oman (2012-2015)",

"Etiology, clinical characteristics, and risk factors associated with severe influenza-like illnesses in Mexican adults\n",

"Evaluation of a new clinical endpoint for moderate to severe influenza disease in children: A prospective cohort study\n",

"Facility-based surveillance for influenza and respiratory syncytial virus in rural Zambia",

"Factors associated with Severe Acute Respiratory Syndrome in a Brazilian central region",

"Factors associated with poor outcomes in patients with severe acute respiratory infections in Bahrain\n",

"First-year results of the Global Influenza Hospital Surveillance Network: 2012-2013 northern hemisphere influenza season\n",

"Five-year community surveillance study for acute respiratory infections using text messaging: findings from the MoSAIC study\n",

"Hospital-based surveillance of influenza A(H1N1)pdm09 virus in Saudi Arabia, 2010-2016\n",

"Hospitalization of influenza-like illness patients recommended by general practitioners in France between 1997 and 2010\n",

"Impact of 2009 pandemic influenza among Vietnamese children based on a population-based prospective surveillance from 2007 to 2011\n\n",

"Impact of influenza infection on children's hospital admissions during two seasons in Athens, Greece\n",

"Impact of influenza on outpatient visits, hospitalizations, and deaths by using a time series Poisson generalized additive model\n",

"Implementing the World Health Organization Pandemic Influenza Severity Assessment framework-Singapore's experience\n",

"Incidence, disease severity, and follow-up of influenza A/A, A/B, and B/B virus dual infections in children: a hospital-based digital surveillance program\n",

"Influenza B associated paediatric acute respiratory infection hospitalization in central Vietnam\n",

"Influenza hospitalization epidemiology from a severe acute respiratory infection surveillance system in Jordan, January 2008-February 2014",

"Lessons from the epidemiological surveillance program, during the influenza a (H1N1) virus epidemic, in a reference university hospital of southeastern Brazil\n",

"Lethality and characteristics of deaths due to COVID-19 in Rondonia: an observational study\n",

"Morbidity, mortality, and seasonality of influenza hospitalizations in Egypt, November 2007-November 2014\n",

"Mortality amongst patients with influenza-associated severe acute respiratory illness, South Africa, 2009-2013",

"Mortality, Severe Acute Respiratory Infection, and Influenza-Like Illness Associated with Influenza A(H1N1)pdm09 in Argentina, 2009\n",

"National retrospective cohort study to identify age-specific fatality risks of comorbidities among hospitalised patients with influenza-like illness in Taiwan\n",

"Non-influenza respiratory viruses in adult patients admitted with influenza-like illness: a 3-year prospective multicenter study\n",

"Novel influenza A(H1N1) in a pediatric health care facility in New York city during the first wave of the 2009 pandemic\n",

"Occurrence of AH1N1 viral infection and clinical features in symptomatic patients who received medical care during the 2009 influenza pandemic in Central Mexico\n",

"Outbreak of 2009 pandemic influenza A(H1N1), Los Lagos, Chile, April-June 2009\n\n",

"Outcomes of influenza A(H1N1)pdm09 virus infection: results from two international cohort studies\n",

"Outcomes of influenza A(H1N1)pdm09 virus infection: results from two international cohort studies\n",

"Outcomes of patients with Severe Acute Respiratory Infections (SARI) admitted to the intensive care unit: results from the Egyptian Surveillance Study 2010-2014",

"Outcomes of patients with Severe Acute Respiratory Infections (SARI) admitted to the intensive care unit: results from the Egyptian Surveillance Study 2010-2014",

"Overview of the winter wave of 2009 pandemic influenza A(H1N1)v in Vojvodina, Serbia\n",

"Pandemic H1N1 influenza in Brazil: Analysis of the first 34,506 notified cases of influenza-like illness with severe acute respiratory infection (SARI)",

"Patient characteristics associated with COVID-19 positivity and fatality in Nigeria: retrospective cohort study\n",

"Persistent Functional Decline Following Hospitalization with Influenza or Acute Respiratory Illness\n",

"Prevalence and risk factors for long COVID after mild disease: A cohort study with a symptomatic control group\n",

"Prevalence, incidence, and severity associated with viral respiratory tract infections in Colombian adults before the COVID-19 pandemic\n",

"Recrudescent wave of pandemic A/H1N1 influenza in Mexico, winter 2011-2012: Age shift and severity\n",

"Respiratory syncytial virus and other respiratory viral infections in older adults with moderate to severe influenza-like illness\n",

"Respiratory virus-associated severe acute respiratory illness and viral clustering in Malawian children in a setting with a high prevalence of HIV infection, malaria, and malnutrition\n",

"Risk Factors for Severe Coronavirus Disease 2019 Among Human Immunodeficiency Virus-Infected and -Uninfected Individuals in South Africa, April 2020-March 2022: Data From Sentinel Surveillance\n",

"Risk Factors for Severe Coronavirus Disease 2019 Among Human Immunodeficiency Virus-Infected and -Uninfected Individuals in South Africa, April 2020-March 2022: Data From Sentinel Surveillance\n",

"Risk factors for influenza-associated severe acute respiratory illness hospitalization in South Africa, 2012-2015",

"Risk factors for influenza-associated severe acute respiratory illness hospitalization in South Africa, 2012-2015",

"Risk factors of prolonged hospital stay in children with viral severe acute respiratory infections\n",

"SARS-CoV-2 and influenza virus coinfection among patients with severe acute respiratory infection during the first wave of COVID-19 pandemic in Bangladesh: a hospital-based descriptive study\n",

"Sentiworld-2022 ACUTE RESPIRATORY ILLNESS SURVEILLANCE REPORT",

"Sentiworld-Weekly Report on Severe Acute Respiratory Infection (SARI), Week 20 2023 (week ending 21/05/2023)",

"Severe Acute Respiratory Infection (SARI) sentinel surveillance in the country of Georgia, 2015-2017",

"Severe Acute Respiratory Infections With Influenza and Noninfluenza Respiratory Viruses: Yemen, 2011-2016",

"Severe Illnesses Associated With Outbreaks of Respiratory Syncytial Virus and Influenza in Adults",

"Severe acute respiratory illness deaths in sub-Saharan Africa and the role of influenza: a case series from 8 countries\n",

"Severe acute respiratory illness surveillance for influenza in Kenya: Patient characteristics and lessons learnt\n",

"Severe acute respiratory infection in children in a densely populated urban slum in Kenya, 2007-2011",

"Severe influenza-associated respiratory infection in high HIV prevalence setting, South Africa, 2009-2011\n",

"Severity and mortality of COVID-19 infection in HIV-infected individuals: Preliminary findings from Iran\n",

"Single- and multiple viral respiratory infections in children: disease and management cannot be related to a specific pathogen",

"Status of novel coronavirus disease and analysis of mortality in Mexico, until June 30th, 2020: An ecological study\n",

"Substantial Morbidity and Mortality Associated with Pandemic A/H1N1 Influenza in Mexico, Winter 2013-2014: Gradual Age Shift and Severity\n",

"Surveillance data for eight consecutive influenza seasons in Sicily, Italy\n",

"Surveillance for hospitalized acute respiratory infection in Guatemala\n",

"Surveillance for severe acute respiratory infections (SARI) in hospitals in the WHO European region - an exploratory analysis of risk factors for a severe outcome in influenza-positive SARI cases\n",

"Surveillance of hospitalised patients with influenza-like illness during pandemic influenza A(H1N1) season in Sicily, April 2009-december 2010\n",

"Surveillance of influenza in Iceland during the 2009 pandemic\n",

"The Burden of Influenza-Associated Hospitalizations in Oman, January 2008-June 2013",

"The Epidemiology and Burden of Influenza B/Victoria and B/Yamagata Lineages in Kenya, 2012-2016\n",

"The Epidemiology and Burden of Influenza B/Victoria and B/Yamagata Lineages in Kenya, 2012-2016\n",

"The Intensive Care Global Study on Severe Acute Respiratory Infection (IC-GLOSSARI): a multicenter, multinational, 14-day inception cohort study\n",

"The emergence of novel SARS-CoV-2 variant P.1 in Amazonas (Brazil) was temporally associated with a change in the age and sex profile of COVID-19 mortality: A population based ecological study",

"The epidemiology and severity of respiratory viral infections in a tropical country: Ecuador, 2009-2016\n",

"The impact of altitude on hospitalization and hospital mortality from pandemic 2009 influenza A (H1N1) virus pneumonia in Mexico\n",

"The impact of pandemic influenza H1N1 on health-related quality of life: A prospective population-based study\n",

"The substantial hospitalization burden of influenza in central China: surveillance for severe, acute respiratory infection, and influenza viruses, 2010-2012\n",

"U-shaped-aggressiveness of SARS-CoV-2: Period between Initial Symptoms and Clinical Progression to COVID-19 Suspicion. A Population-Based Cohort Study\n",

"Using research to prepare for outbreaks of severe acute respiratory infection",

"Using routine emergency department data for syndromic surveillance of acute respiratory illness, Germany, week 10 2017 until week 10 2021",

"Viral etiology, seasonality and severity of hospitalized patients with severe acute respiratory infections in the Eastern Mediterranean Region, 2007-2014\n"

), DETAILS_study_aim = c("We compare the epidemiology and clinical presentation of seasonal influenza A (H1N1 and H3N2) and 2009 pandemic influenza A (H1N1) (pH1N1) using a prospective surveillance system for acute respiratory disease in Guatemala.",

"We compare the epidemiology and clinical presentation of seasonal influenza A (H1N1 and H3N2) and 2009 pandemic influenza A (H1N1) (pH1N1) using a prospective surveillance system for acute respiratory disease in Guatemala.",

"This study aimed to summarize the epidemiological and clinical characteristics of COVID-19 from Western Mexico people during 2020.",

"This is a prospective study comparing the demographics, clinical profile, co-infection with other viruses, and predictors of mortality in COVID-19 patients presenting with typical symptoms of an ILI to those with atypical symptoms and would help our primary care physicians in managing such cases with limited resources.",

"We examined the association between a subjective symptom severity score, demographic and clinical characteristics, and presence of laboratory-confirmed influenza among central Wisconsin adults who sought care for ARI during four influenza seasons.",

"This report summarizes the results of this surveillance and\ndescribes the dynamics and impact of the influenza pan-\ndemic on Re\xefunion Island and the characteristics of labora-\ntory-confirmed cases, including hospitalized, severe and fatal\ncases.",

"In this study, therefore, we aimed to identify risk factors for SARI and pneumonia among adult patients with ARI during the 2011-2014 influenza seasons using HIMM surveillance data in Korea.",

"In this study, we report on the prevalence of antibiotic use and its associated factors among suspected and confirmed COVID-19 patients admitted to 35 health facilities in Sierra Leone from March 2020?March 2021.",

"We aimed to quantify life course-specific associations between death in hospital and 30 chronic conditions, and comorbidity among them, in adults (aged 20+ years) during their first acute care hospitalization with a confirmed or suspected COVID-19 diagnosis in Canada.",

"Flutracking data for 2011 and 2012 were used to investigate whether a near real time severity measure for circulating influenza strains could be determined.",

"This report describes demographic characteristics of enrolled children who received care in these settings, and yearly circulation of influenza, RSV, HMPV, HPIV1?3, adenovirus, human rhinovirus and enterovirus (RV/EV),* and SARS-CoV-2 during December 2016?August 2021.",

"In addition to statutory quality assurance, more than 500 hospitals in Germany and Switzerland are voluntarily organised in the ?Initiative of Quality Medicine? (IQM, Berlin, Germany).7 Utilizing a large-scale administrative dataset derived from the IQM, we conducted an extended analysis of SARI and COVID-19 cases covering four pandemic waves to report on patient characteristics and respective outcomes and compare pre-pandemic with pandemic time period",

"The authors evaluated surveillance of acute respiratory infections (ARI), influenza and influenza-like illnesses (ILI) in the Slovak Republic (SR).\nThey analyze morbidity, age-specific morbidity, complications, mortality, number of influenza viruses isolations and vaccination coverage rates in\nthe SR in the years 1993?2008.",

"there is limited published data on which respiratory viral pathogens cause ARI in children in the UK. Our study has attempted to address this knowledge gap by describing viral pathogen prevalence, occurrence of co-infection, diagnostic yield of sampling methods and presence of co-morbidity in patients with ARI caused by 2009 pdmH1N1 and other respiratory viruses, in a large paediatric teaching hospital in the North West of England over a year between April 2010 and March 2011.",

"Here, we analyze the age- and state-specific incidence of influenza morbidity and mortality in 32 Mexican States, on the basis of reports to the Mexican Institute for Social Security (IMSS), a private medical system that covers 40% of the Mexican population. We also quantify the association between local influenza transmission rates, school cycles, and demographic factors.",

"We aimed to compare the characteristics and outcome of respiratory virus infections in adults hospitalized for influenza-like illness (ILI) with, or without, chronic use of ICS.",

"The present study aims to analyze the clinical features and outcome in children infected with SARS-CoV-2 in tertiary care pediatric teaching hospital in Northern India..",

"Our objective was to describe cases and deaths from severe acute respiratory infection (SARI) in Brazil over the past 8 y as well as changes in the distribution and risk of illness and death from SARI before and in the first year of the coronavirus disease 2019 (COVID-19) pandemic (FYP).",

"We first aimed to report the epidemiological dynamics of the 2019?20 season at the GIHSN scale for the influenza-associated hospitalisation of patients aged over 5 years. The second objective was to describe the phylogenetic characterization of the viruses responsible for these hospitalisations for the first time within our network, with a specific focus on the representativeness of these cases compared to other medically attended influenza surveillance networks.",

"Studies on coronavirus disease 2019 (COVID-19) have mainly focused on hospitalized patients or those with severe disease. We aim to assess the clinical characteristics, outcomes and factors associated with hospital admission or death in adult outpatients with COVID-19.",

"We described the clinical and epidemiological characteristics of children with influenza and analyzed the association between potential risk factors and SARI patients with influenza.",

"The aim of this study was to analyse characteristics and outcome of respiratory syncytial virus (RSV) infection in adults hospitalized with influenza-like illness (ILI).",

"Recognizing the need to investigate both influenza and non-influenza ILIs in the Mexican population, La Red implemented a study with the objective of describing the etiology, symptoms, and outcomes of subjects presenting with ILI in Mexico City.",

"This study describes the clinical features and outcomes of patients with severe acute respiratory infections (SARI) in hospitalized patients in Egypt.",

"The objective of the present study was to examine the clinical and laboratory as well as comorbidities and outcomes of the SARI patients admitted in the COVID-19 suspect ICU.",

"In this study, we investigated the epidemiological patterns of COVID-19 infections, hospitalizations, deaths, and factors associated with severe disease outcomes during the five epidemic waves in Mexico. To that end, we used data reported by the Mexican Institute of Social Security (IMSS) surveillance system, the most extensive Latin-American social security system, and Mexico?s leading health institution [6].",

"Multiple case definitions are used to identify hospitalized patients with community-acquired acute respiratory infections (ARI). We evaluated several commonly used hospitalized ARI case definitions to identify influenza cases.",

"In our prior viral surveillance studies in Amman Jordan, over 80% of young hospitalized children with fever and/or ARI had at least one respiratory virus identified; however, testing for the 4 common HCoVs was not performed.17 Furthermore, viral surveillance studies in the Middle East and North Africa region assessing the incidence, seasonality, and clinical characteristics of individual HCoV species are limited. Therefore, this study aimed to assess the clinical characteristics and distribution of HCoV infections in hospitalized children less than 2 years over 3 respiratory seasons.",

"Editorial Note\n\nThis is the first report to summarize the epidemiology of 2009 H1N1 in Greece. During July--August 2009, Greece experienced a moderate wave of transmission, followed by a stronger wave beginning in October and peaking during November 23--29.",

"The aim of the present study was toclarify the characteristics of hospitalized cases ofA(H1N1)pdm09 infection on the basis of analyses of thesurveillance data collected during the 2009/2010 season.",

"The aim of the present study was toclarify the characteristics of hospitalized cases ofA(H1N1)pdm09 infection on the basis of analyses of thesurveillance data collected during the 2009/2010 season.",

"In this paper, we describe the descriptive epidemiology of A (H1N1) cases reported in Andhra Pradesh during May 2009 to December 2010",

"Thus, beginning in May 2013, the Minnesota Department of Health (MDH) with the support of the Centers for Disease Control and Prevention (CDC) incorporated molecular testing for influenza C into existing sentinel surveillance systems for outpatient and inpatient ARI, allowing us to study the epidemiology of influenza C virus infection.",

"Thus, beginning in May 2013, the Minnesota Department of Health (MDH) with the support of the Centers for Disease Control and Prevention (CDC) incorporated molecular testing for influenza C into existing sentinel surveillance systems for outpatient and inpatient ARI, allowing us to study the epidemiology of influenza C virus infection.",

"In the present study, we aimed to describe the detection of viruses and bacteria in hospitalized children with ARI in a subtropical city of mainland China, investigate the simultaneous detection pattern of multiple viruses and bacteria, and assess their association with clinical outcomes and severity.",

"We sought to describe the process of digitizing and upgrading SARI surveillance in Malta, an island country with a centralized health system, during the COVID-19 pandemic from February to November 2021. We described the characteristics of people included in the surveillance system and compared different SARI case definitions, including their advantages and disadvantages. This study also discusses the process, output, and future for SARI and other public health surveillance opportunities.",

"The primary goal of this prospective, population-based study was to determine whether racial disparities existed between black and white children under 5 years of age for hospitalizations associated with community-acquired ARI and laboratory-confirmed seasonal influenza and RSV illness in 3 large US counties. Secondarily, we assessed racial differences in access to care, influenza vaccination, and other characteristics that may affect racial disparities in hospitalization rates.",

"\nObjectives\n\nTo measure quality-adjusted life days and years (QALDs and QALYs) lost and work/school absences among community cases of acute respiratory infections (ARI), ILI and influenza A and B and to estimate community burden of QALY loss and absences from influenza.\n",

"\nObjectives\n\nTo measure quality-adjusted life days and years (QALDs and QALYs) lost and work/school absences among community cases of acute respiratory infections (ARI), ILI and influenza A and B and to estimate community burden of QALY loss and absences from influenza.\n",

"To characterize the epidemiology and transmission\ndynamics of influenza and risk factors for influenza-associated severe\nrespiratory infection in Uganda.",

"To characterize the epidemiology and transmission\ndynamics of influenza and risk factors for influenza-associated severe\nrespiratory infection in Uganda.",

"we evaluated the features (demographic data,hospitalization information, and outcomes) of hospitalizedpatients with SARI in Brazil, during the COVID-19 pandemic,according to the following groups: SARI due to Influenza virusinfection, SARI due to other respiratory viruses? infection,SARIdue to other known etiologic agents (OEAs), SARI due toSARS-CoV-2 infection (patients with COVID-19), and SARI dueto an undefined etiological agent",

"In this article we fill this gap and carry out an analysis of clinical features at presentation, hospital admission delays, medical conditions, and receipt of seasonal vaccine on the risk of A/H1N1-related death among hospitalized patients. We use individual-level data from a prospective surveillance system implemented by the largest Mexican Social Security medical system spanning August-December, 2009.",

"Here we report on the epidemiology of a recrudescent (fourth) wave of pandemic A/H1N1 influenza activity in Mexico from December 1, 2011?March 20, 2012 and update our preliminary findings based on data up to February 10, 2012 (10).",

"However, characterization of the epidemiological features of COVID-19 is crucial for the development and implementation of effective control strategies to reduce the socioeconomic effects of the COVID-19 pandemic. Here, we report the results of a descriptive, exploratory analysis of all of the cases diagnosed between March 2020 to October 2021 in Sierra Leone to better understand the epidemic's progression and to formulate targeted strategies to contain current and future viral outbreaks.",

"We carried out a retrospective analysis of the available information, in order to study the clinico-epidemiological features and establish the magnitude and severity of recent Influenza A(H1N1)pdm09 epidemics in hospitalized patients from the state of Odisha.",

"This report presents the results of the national laboratory?based surveillance for hospitalized patients with ARI in 284 hospitals all over Egypt, 2016?2019. The study aims at describing the epidemiology and exploring severity and mortality of influenza?associated infections among hospitalized ARI patients to identify target groups for influenza prevention and control strategies.",

"Our study aimed to describe the epidemiology and viral etiology of ARI in the first year of life within an ongoing prospective infant cohort in southern Vietnam.",

"To investigate the clinical and epidemiological features of influenza virus A/H1N1, A/H3N2, and B infection in Fars province, southern Iran, in 2015-2019.",

"To investigate the clinical and epidemiological features of influenza virus A/H1N1, A/H3N2, and B infection in Fars province, southern Iran, in 2015-2019.",

"In this high HIV prevalence and malaria-endemic setting, we aimed to describe the epidemiology and viral etiology and factors associated with clinical severity and influenza positivity among individuals aged ? 15 years with severe acute respiratory illness (SARI) during 2011?2013",

"Here, we describe the epidemiology of hospital admissions with influenza during the 2013/2014 influenza season in the GIHSN Northern hemisphere participating sites. We also determine the impact of underlying patient characteristics on the risk of hospital admission and complications due to influenza overall and due to influenza A(H1N1)pdm09, A(H3N2), and B/Yamagata lineage.",

"The aim of this study was to analyze cases of IBV infection amongcases of ARI and SARI notified between 2003 and 2018 in RioGrande do Sul, Southern Brazil, and analyze demographic and clinicaldata, as well as IBV circulation over the years.",

"The aim of this study was to analyze cases of IBV infection amongcases of ARI and SARI notified between 2003 and 2018 in RioGrande do Sul, Southern Brazil, and analyze demographic and clinicaldata, as well as IBV circulation over the years.",

"There are few published studies describing severe acute respiratory illness (SARI) epidemiology amongst older children and adults from high HIV-prevalence settings. We aimed to describe SARI epidemiology amongst individuals aged ?5 years in South Africa.",

"This study aims to better describe the epidemiology, disease severity, and outcome of SARS-CoV-2/Flu coinfection to guide the development of effective preventive and control measures including case management and vaccination policy",

"To establish a hospital-based platform to explore the epidemiological and clinical characteristics of patients screened for COVID-19.",

"We described the establishment of an ICD-10-based inpatient syndromic sentinel system and its application to the analysis of five influenza seasons. We compared the impact of different case definitions on the ability to capture SARI cases, to allow a timely trend analysis of the seasonal epidemic and to reflect the burden caused by influenza when compared to routine outpatient surveillance.",

"To implement the World Health Organization?s pandemic influenza severity assessment tool in Australia, using multiple sources of data to establish thresholds and measure influenza severity indicators.",

"To implement the World Health Organization?s pandemic influenza severity assessment tool in Australia, using multiple sources of data to establish thresholds and measure influenza severity indicators.",

"Objectives: To estimate age-specific hospitalization rates associated with laboratory-confirmed A(H1N1)pdm09 virus in Davidson County, TN, from May 2009 to March 2010.",

"The most important complication of influenza virus infection is pneumonia (primary viral or secondary bacterial pneumonia) [9]. While costs can be high, the precise burden remains a blind spot [10?15]. Such burden information is crucial for prevention and response considering that vaccination, the main control measure against influenza infection, is aimed at preventing complications. A severe influenza season may also lead to hospital capacity problems, especially in ICUs. In this study we analyze comprehensive retrospective ICU data to fill the current knowledge gap.",

"To estimate the incidence of influenza- associated hospitalizations and in- hospital death in Oman",

"Objective\n\nThe aim of this study was to determine the risk factors associated with severe influenza-like illness (ILI) in Mexican adults that could be useful to clinicians when assessing patients with ILI.",

"Therefore, the objectives of this study were to evaluate if the proposed definition of moderate to severe influenza in children predicts hospitalization and other clinically relevant healthcare endpoints including recurrent ED visits, use of antimicrobials (including antivirals), school/daycare or parental work absenteeism, and increased healthcare costs during 2 recent influenza seasons.",

"In December 2018, facility-based surveillance for influenza and RSV was established in rural Zambia to evaluate their role in causing respiratory illness and begin to situate rural Zambia in the landscape of regional and global virus transmission. The objective of this analysis was to describe the burden of influenza and RSV disease during the first year of surveillance and explore predictors of severe disease.",

"This study aimed to analyze the epidemiological profile and the factors associated with hospitalization and deaths from SARI in a central Brazilian region from 2013 to 2018.",

"Therefore, this study was conducted with the main objective of identifying the risk factors associated with poor outcomes, including mortality, ICU admission, and mechanical ventilation among patients admitted with SARI in Bahrain.",

"In this report, we evaluated the characteristics of hospitalizations related to influenza and the temporal and geographic distribution of the different influenza viruses in these cases during the 2012?2013 Northern hemisphere influenza season, the program?s first year.",

"The objectives of this 5-year community surveillance study were to describe ARI incidence, etiology, and factors associated with infection and care-seeking, as well as to evaluate use of text messaging for longitudinal surveillance in a low-income population.",

"Describe the data generated by the influenza A(H1N1) pdm09 surveillance in Saudi Arabia from 2010 to 2016.",

"In the present study, we compare the PRH of the 2009?\n2010 A(H1N1) pandemic with the twelve preceding sea-\nsonal epidemics, stratifying by age, sex, and viral subtype.\nWe also investigate the reasons why GPs recommended\nhospitalization and the presence of risk factors for pan-\ndemic A(H1N1) complications. We finally evaluate the use-\nfulness of this surveillance for public health information by\nmonitoring the precision of the PRH estimate throughout\nthe pandemic.",

"To investigate the impact of A(H1N1)pdm09 on pediatric ARI in Vietnam.",

"The aim of the present study was to prospectively evaluate the\nburden of influenza infection upon pediatric hospitalizations in our\narea, and to obtain data on the most common clinical manifesta-\ntions and possible complications.",

"In this study, the time series Poisson generalized additive model (GAM) was used to quantitatively assess the disease burden of influenza and ILI, by using the influenza surveillance data in Zhuhai City from 2007 to 2009 combined with outpatient, inpatient, and respiratory disease mortality data from the same period.",

"In this paper, we document Singapore's experience in developing and evaluating the PISA indicators and parameters, and this would provide other countries with suggestions that they can use in developing their own indicators.",

"Within the framework of this inception cohort, we leveraged uniform case classi-fication of ILI and respiratory infection, standardized longitudinal patient assessments,and a comparable disease severity measure in addition to comprehensive IV PCR testingto investigate\n?The incidence of IV dual infections in children based on a hospital-based digitalsurveillance program with a known denominator of all ILI cases and\n?Disease severity, symptoms, and detailed course of illness in children with IVdual infections",

"In this study, we investigated the incidence and clinical?epidemiological characteristics of paediatric hospitalized influenza B ARI cases in Vietnam.",

"We aim to describe the epidemiology and seasonality of influenza hospitalizations in Jordan over a time period of 6\xffyears (2008?2014).",

"to evaluate the impact of two definitions used as epidemiological tools, in adults and children, during the influenza A H1N1 epidemic.",

"Objective: To describe the characteristics of deaths due to COVID-19 in the state of Rond\x93nia.",

"The aims of this study were to (1) assess the proportion of SARI cases having influenza infection in Egypt; (2) examine the types and subtypes of detected influenza viruses in Egypt; (3) compare demographic and clinical characteristics of influenza-positive SARI cases to those of influenza-negative SARI cases in Egypt; (4) quantify influenza deaths and assess influenza mortality risk factors in Egypt; and (5) establish a defined period of influenza seasonality in Egypt.",

"We aimed to estimate the incidence of influenza-associated severe acute respiratory illness (SARI) deaths and describe the risk-factors associated with death using data from prospective, hospital-based sentinel surveillance in South Africa.",

"In order to explore whether Argentina's influenza A(H1N1)pdm09 burden was higher or similar to the burden documented elsewhere, we use active facility-based influenza surveillance and health utilization surveys from three cities in Argentina to estimate rates of influenza A(H1N1)pdm09-associated mortality, hospitalization, and influenza-like illnesses.",

"Objectives This study aimed to examine comprehensively\nthe prognostic impact of underlying comorbidities among\nhospitalised patients with influenza-like illness (ILI) in\ndifferent age groups and provide recommendations\ntargeting the vulnerable patients",

"To describe the burden, and characteristics, of influenza-like illness (ILI) associated with non-influenza respiratory viruses (NIRV).",

"Objective To describe the burden of care experienced by our pediatric health care facility in New York, New York, from May 3, 2009, to July 31, 2009, during the novel influenza A(H1N1) pandemic that began in spring 2009.",

"This study estimated the AH1N1 infection, hospitalization and mortality rates in SLP during the 2009 pandemic, and aimed at identifying clinical features associated with AH1N1 infection in individuals with flu-like illness who sought medical care.",

"The aim of this study, as outlined in the introduction, was to investigate an outbreak of the 2009 pandemic influenza A(H1N1) in the Los Lagos region of Chile. Key objectives of the study included:",

"In this report, we describe outcomes of outpatients and hospitalized patients with influenza A(H1N1)pdm09 virus infection and examine risk factors for progression of their illness.",

"In this report, we describe outcomes of outpatients and hospitalized patients with influenza A(H1N1)pdm09 virus infection and examine risk factors for progression of their illness.",

"We aimed to investigate the role of different respiratory viruses in causing critical illness requiring ICU admission, which pathogens were related to severe outcomes, and to address the impacts of SARI on the clinical outcomes of patients admitted to the ICU, in terms of morbidity and mortality.",

"We aimed to investigate the role of different respiratory viruses in causing critical illness requiring ICU admission, which pathogens were related to severe outcomes, and to address the impacts of SARI on the clinical outcomes of patients admitted to the ICU, in terms of morbidity and mortality.",

"To analyze the epidemiological data for pandemic influenza A(H1N1)v in the Autonomous Province of Vojvodina, Serbia, during the season of 2009/2010 and to assess whether including severe acute respiratory illness (SARI) hospitalization data to the surveillance system gives a more complete picture of the impact of influenza during the pandemic.",

"The present paper describes the epidemiological profile of\ninfluenza-like illness (ILI) with severe acute respiratory infection\n(SARI), occurred during EW16 to 33 in Brazil. Case-fatality by sex\nand presence of comorbidity is also presented.",

"Despite the increasing disease burden, there is a dearth of context-specific evidence on the risk factors for COVID-19 positivity and subsequent death in Nigeria. Thus, the study objective was to identify context-specific factors associated with testing positive for COVID-19 and fatality in Nigeria.",

"We aimed to investigate persistent functional change in older adults admitted to hospital with influenza and other acute respiratory illness (ARI).",

"There is limited data on the prevalence and risk factors for long COVID and few prospective studies with appropriate control groups and adequate sample sizes. We performed a prospective study to determine the prevalence and risk factors for long COVID.",

"We hypothesize that the most frequent clinical diagnosis of patients admitted due to ARI is CAP. The severity classification used by the surveillance program might identify patients at higher risk of worse clinical outcomes. Using the ARI-based report strategy in Bogot\xa0 (Colombia), we will attempt to bridge this gap in the literature by identifying the clinical burden of respiratory viral infections, the incidence per year, disease severity, and clinical outcomes of ARI in adults.",

"Here we describe changes in the epidemiological patterns of the ongoing 4th pandemic wave in 2011-12, relative to the earlier waves in 2009. The analysis is intended to guide public health intervention strategies in near real time.",

"We used multiplex reverse transcriptase PCR (RT-PCR) to identify viral respiratory pathogens in nasal and throat swabs from episodes of moderate-to-severe influenza-like illness (ILI) in influenza-vaccinated elderly individuals.",

"In the context of a low-income population with multiple drivers of immune compromise (eg, human immunodeficiency virus [HIV] infection, malnutrition, and malaria) [11], we conducted active surveillance at a large urban teaching hospital in Malawi to estimate the incidence of childhood SARI and explore the association of SARI clinical severity with HIV infection and clustering of respiratory viral coinfection.\n",

"Using a well established syndromic surveillance program for influenza-like-illness (ILI) [11] and severe respiratory illness (SRI) [12?14], we aimed to describe clinical and epidemiological characteristics of persons with laboratory-confirmed COVID-19 and identify factors associated with COVID-19 hospitalization or mortality.",

"Using a well established syndromic surveillance program for influenza-like-illness (ILI) [11] and severe respiratory illness (SRI) [12?14], we aimed to describe clinical and epidemiological characteristics of persons with laboratory-confirmed COVID-19 and identify factors associated with COVID-19 hospitalization or mortality.",

"Risk factors for influenza-associated severe acute respiratory illness hospitalization in South Africa, 2012-2015\n",

"Risk factors for influenza-associated severe acute respiratory illness hospitalization in South Africa, 2012-2015\n",

"The present study focused on detection of risk factors for prolonged hospital stay among children with viral SARI, including demographic and clinical characteristics of patients, and the type and seasonality of different respiratory viral pathogens causing acute lower respiratory infection (ALRI).",

"To estimate the proportion of SARS-CoV-2 and influenza virus coinfection among severe acute respiratory infection (SARI) cases-patients during the first wave of COVID-19 pandemic in Bangladesh.",

"This report provides an overview of priority viral respiratory illnesses in New Zealand in 2022,\nincluding those causing influenza, respiratory syncytial virus (RSV) illness and COVID-19. Please\nnote, most viral respiratory illnesses are not legally notifiable diseases in New Zealand.",

"This report includes data on SARI hospitalised cases, aged 15 years and older who were admitted to\nSt. Vincent?s University Hospital (SVUH), Dublin up to week 20 2023.",

"This study aimed to characterize the seasonality and epidemiology of SARI in the country of Georgia over two influenza seasons (2015?2016 and 2016?2017), to describe the etiological and clinical patterns observed, and to assess seasonal influenza vaccine effectiveness using a case-test negative design.",

"This study aimed to determine the proportions of influenza and noninfluenza virus among SARI patients, and assess the severity of SARI and its associated factors in Yemen.",

"Our purpose of the present study was to perform an ecological analysis of the relationship between outbreaks of RSV and flu and advanced medical outcomes of adults in a defined geographic region over 12 consecutive years (2001 to 2013).",

"Hospital-based influenza surveillance has increased dramatically in Africa in the last decade [9], and we assessed whether the current systems could provide insights into seasonal influenza?associated mortality among persons hospitalized for respiratory disease in Africa, as well as data on other etiologies of respiratory disease?associated mortality in sub-Saharan Africa.",

"This paper aims to describe the epidemiology and clinical features of patients hospitalized with influenza in Kenya and highlight the importance of year?round surveillance for severe acute respiratory infections (SARI), especially in tropical countries.",

"To broaden the knowledge-base and compare etiology and epidemiology, we analyzed data from our population-based infectious disease surveillance (PBIDS) site in Kibera, an urban slum in Nairobi. The rural and urban PBIDS operate with the same study protocol.",

"We investigated the incidence of hospitalization for influenza-associated acute lower respiratory tract infection (LRTI) and the clinical course of illness in persons with and without HIV infection in South Africa.",

"This study aims to investigate the course of COVID-19 infection in HIV-infected individuals by characterizing COVID-19 incidence, clinical presentation, severity, and mortality in HIV-infected patients as compared to HIV-negative COVID cases in Iran. Since the study has been performed in the early stage of the COVID-19 pandemic, few similar evidences have been published in this regard, especially in the study area indicating the novelty of the study.",

"The aim of the current study was to determine if RT-PCR test results are related to clinical data in children with respiratory symptoms. We investigated clinical symptoms, management and outcome in these children and correlated these findings to the specific virus determined by RT-PCR. We additionally investigated clinical differences between single-, multiple-, and RT-PCR negative ARI.",

"The aim was to analyze the Cause-Specific Mortality Rate (CSMR) for COVID-19, for each Mexican State and the effect of comorbidities on deaths by COVID-19.",

"Here we report preliminary findings on the epidemiology of the on-going A/H1N1 outbreak in Mexico from October 2013 to January 2014. Because past influenza pandemics have had substantial morbidity and mortality burden for several seasons after the initial pandemic waves 6 7 8 9 10 , continued vigilance is prudent. We compare the demographic and clinical characteristics of laboratory-confirmed A/H1N1 hospitalizations and deaths in winter 2013-14 with those reported for the preceding 2011-12 A/H1N1 epidemic. Our data highlight a change in the age distribution of A/H1N1 patients and a slightly higher reproduction number compared to the 2011-12 A/H1N1 outbreak.",

"This retrospective study aimed to explore the epidemiology of influenza disease and the heterogeneity of circulating strains in Sicily, at the primary care and hospital level, over eight influenza seasons after the 2009 pandemic.",

"The International Emerging Infections Program of the U.S. Centers for Disease Control and Prevention (CDC), in collaboration with the Guatemala Ministry of Public Health and Welfare and the Universidad del Valle de Guatemala (UVG) conducts surveillance for hospitalized ARI in two sites in Guatemala. The surveillance is aimed at measuring the burden of hospitalized ARI in the catchment area and characterizing ARI etiology. We present the findings of surveillance for hospitalized ARI from November 2007 through December 2011.",

"This paper describes the characteristics of SARI patients and investigates risk factors for a severe outcome (ICU/fatal) in influenza-positive SARI patients in countries in Central and Eastern Europe.",

"The aim of the present study was to report the influenza surveillance data describing the epidemiological characteristics of patients with ILI symptoms, laboratory-confirmed infections with pandemic influenza A(H1N1)2009, and fatal cases that occurred among hospitalised patients in Sicily from April 2009 through December 2010.",

"In this article we report the changes made in the surveillance of influenza in Iceland and describe the data collected during the 2009 H1N1pandemic.",

"This report describes the establishment of a SARI surveillance system in Oman and presents the epidemiology and seasonality of influenza during the period of January 2008 to June 2013.",

"Here, we describe the epidemiology and clinical presentation associated with influenza B virus lineages (B/Victoria and B/Yamagata) among medically attended cases of acute respiratory illness (ARI) in Kenya.",

"Here, we describe the epidemiology and clinical presentation associated with influenza B virus lineages (B/Victoria and B/Yamagata) among medically attended cases of acute respiratory illness (ARI) in Kenya.",

"In this prospective, multicenter, 14-day inception cohort study, we investigated the epidemiology, patterns of infections, and outcome in patients admitted to the intensive care unit (ICU) as a result of severe acute respiratory infections (SARIs).",

"With the objective to describe and to identify possible changes in the mortality profile associated temporally to the emergence of the P1 strain in the state of Amazonas, we used public data of COVID-19 cases registered at the national epidemiological surveillance system. Two distinct epidemiological periods were considered in our analysis: the peak of the first wave, between April and May 2020, and January 2021 (the second wave), the month in which the new variant came to predominate.",

"Respiratory viral infections (RVI) are a leading cause of mortality worldwide. We compared the epidemiology and severity of RVI in Ecuador during 2009?2016.",

"In this study, our objective was to examine the association between altitude of residence and rates of hospitaliza-tion and mortality, in cases of Influenza?like illness (ILI) and severe acute respiratory illness (SARI), during the first months of the 2009 pandemic influenza A H1N1 virus, to examine if residents of high altitude had more frequently these adverse outcomes.",

"The health-related quality of life detriment from a population-based sample of confirmed H1N1v patients was prospectively measured and compared to controls who were investigated because they had influenza like illness (ILI), but were not laboratory confirmed as H1N1v. The aims were: 1) to quantify the burden of H1N1v for individual patients and investigate factors, such as age and treatment with antivirals, that may affect this; 2) compare the severity of the 2009 strain to other infections that cause ILI and previous estimates of the severity of influenza from a systematic literature review; and 3) to estimate the overall burden attributed to H1N1v in the population. The findings can then be used to inform effectiveness and cost-effectiveness analyses on policy decisions related to the control of future waves of this or related viruses.",

"The study aimed to conduct surveillance of Severe Acute Respiratory Infection (SARI) in central China and estimate the rates of hospitalization due to SARI that could be attributed to influenza virus infections. This involved analyzing the prevalence and impact of different influenza virus types/subtypes in hospitalized SARI patients.",

"To determine the aggressiveness of SARS-CoV-2 by analyzing symptom progression in COVID-19 patients.",

"We present combined results from the first two seasons of data collection for this programme during 2016?2017, where the primary circu-lating respiratory viruses were influenza A (H3N2) and A(H1N1)pdm09.",

"In this work, we aimed to describe emergency-department attendances for acute respiratory illness in Germany over time, for the purpose of developing and implementing syndromic surveillance.",

"Describe viral etiology of ARI in Eastern Med 2007 -14"), DETAILS_publication_type = c("Peer-reviewed article",

"Peer-reviewed article", "Peer-reviewed article", "Peer-reviewed article",

"Peer-reviewed article", "Peer-reviewed article", "Peer-reviewed article",

"Peer-reviewed article", "Peer-reviewed article", "Peer-reviewed article",

"Surveillance report", "Peer-reviewed article", "Peer-reviewed article",

"Peer-reviewed article", "Peer-reviewed article", "Peer-reviewed article",

"Peer-reviewed article", "Peer-reviewed article", "Peer-reviewed article",

"Peer-reviewed article", "Peer-reviewed article", "Peer-reviewed article",

"Peer-reviewed article", "Peer-reviewed article", "Peer-reviewed article",

"Peer-reviewed article", "Peer-reviewed article", "Peer-reviewed article",

"Surveillance report", "Peer-reviewed article", "Peer-reviewed article",

"Peer-reviewed article", "Peer-reviewed article", "Peer-reviewed article",

"Peer-reviewed article", "Peer-reviewed article", "Peer-reviewed article",

"Peer-reviewed article", "Peer-reviewed article", "Peer-reviewed article",

"Peer-reviewed article", "Peer-reviewed article", "Peer-reviewed article",

"Peer-reviewed article", "Peer-reviewed article", "Peer-reviewed article",

"Peer-reviewed article", "Peer-reviewed article", "Peer-reviewed article",

"Peer-reviewed article", "Peer-reviewed article", "Peer-reviewed article",

"Peer-reviewed article", "Peer-reviewed article", "Peer-reviewed article",

"Peer-reviewed article", "Peer-reviewed article", "Peer-reviewed article",

"Peer-reviewed article", "Peer-reviewed article", "Peer-reviewed article",

"Peer-reviewed article", "Peer-reviewed article", "Peer-reviewed article",

"Peer-reviewed article", "Peer-reviewed article", "Peer-reviewed article",

"Peer-reviewed article", "Peer-reviewed article", "Peer-reviewed article",

"Peer-reviewed article", "Peer-reviewed article", "Peer-reviewed article",

"Peer-reviewed article", "Peer-reviewed article", "Peer-reviewed article",

"Peer-reviewed article", "Peer-reviewed article", "Peer-reviewed article",

"Peer-reviewed article", "Peer-reviewed article", "Peer-reviewed article",

"Peer-reviewed article", "Peer-reviewed article", "Peer-reviewed article",

"Peer-reviewed article", "Peer-reviewed article", "Peer-reviewed article",

"Surveillance report", "Peer-reviewed article", "Peer-reviewed article",

"Peer-reviewed article", "Peer-reviewed article", "Peer-reviewed article",

"Surveillance report", "Peer-reviewed article", "Peer-reviewed article",

"Peer-reviewed article", "Peer-reviewed article", "Peer-reviewed article",

"Peer-reviewed article", "Peer-reviewed article", "Peer-reviewed article",

"Peer-reviewed article", "Peer-reviewed article", "Peer-reviewed article",

"Peer-reviewed article", "Peer-reviewed article", "Surveillance report",

"Surveillance report", "Peer-reviewed article", "Peer-reviewed article",

"Peer-reviewed article", "Peer-reviewed article", "Peer-reviewed article",

"Peer-reviewed article", "Peer-reviewed article", "Peer-reviewed article",

"Peer-reviewed article", "Peer-reviewed article", "Peer-reviewed article",

"Peer-reviewed article", "Peer-reviewed article", "Peer-reviewed article",

"Surveillance report", "Surveillance report", "Peer-reviewed article",

"Peer-reviewed article", "Peer-reviewed article", "Peer-reviewed article",

"Peer-reviewed article", "Peer-reviewed article", "Peer-reviewed article",

"Peer-reviewed article", "Peer-reviewed article", "Peer-reviewed article",

"Peer-reviewed article", "Peer-reviewed article", "Peer-reviewed article"

), DETAILS_publication_date = c("30/12/2010", "30/12/2010", "11/10/2021",

"17/04/2023", "01/05/2014", "28/01/2010", "22/11/2016", "28/03/2022",

"01/04/2023", "11/11/2013", "07/10/2022", "08/05/2023", "01/03/2011",

"19/06/2014", "24/05/2011", "10/03/2022", "27/07/2021", "05/08/2022",

"14/05/2022", "27/04/2021", "20/09/2016", "27/11/2016", "14/02/2013",

"01/04/2019", "30/09/2021", "31/10/2022", "25/03/2021", "01/01/2021",

"11/06/2010", "24/12/2014", "24/12/2014", "01/07/2013", "23/10/2017",

"23/10/2017", "24/04/2015", "05/12/2022", "22/02/2013", "09/10/2017",

"09/10/2017", "01/12/2016", "01/12/2016", "01/07/2022", "16/07/2012",

"16/10/2012", "29/06/2022", "15/10/2019", "07/05/2021", "24/03/2015",

"01/03/2021", "01/03/2021", "23/07/2018", "19/05/2016", "26/01/2021",

"26/01/2021", "23/02/2015", "17/11/2022", "02/12/2021", "30/06/2017",

"25/06/2018", "25/06/2018", "23/02/2012", "19/12/2018", "05/12/2017",

"01/02/2023", "14/11/2019", "21/09/2021", "12/08/2020", "26/04/2023",

"05/06/2014", "17/01/2022", "06/02/2020", "22/03/2012", "07/03/2014",

"18/12/2010", "19/02/2016", "17/10/2019", "14/03/2022", "28/02/2019",

"29/01/2016", "22/07/2011", "01/12/2022", "08/09/2016", "18/03/2015",

"31/10/2012", "24/06/2019", "13/02/2020", "04/01/2010", "20/12/2012",

"07/01/2010", "08/07/2014", "08/07/2014", "09/06/2020", "09/06/2020",

"01/04/2011", "22/10/2009", "17/12/2020", "08/12/2020", "12/05/2023",

"01/12/2022", "26/03/2012", "29/01/2014", "13/09/2016", "02/11/2022",

"02/11/2022", "10/02/2017", "10/02/2017", "15/10/2014", "29/11/2021",

"24/07/2023", "21/05/2023", "30/07/2018", "23/04/2019", "04/04/2019",

"01/10/2019", "14/03/2022", "25/02/2015", "01/11/2013", "10/03/2021",

"11/01/2017", "30/12/2020", "26/03/2014", "30/09/2019", "31/12/2013",

"08/01/2015", "01/09/2011", "09/12/2010", "07/12/2015", "30/09/2019",

"30/09/2019", "15/02/2016", "18/07/2021", "17/12/2018", "01/01/2013",

"02/03/2011", "10/11/2013", "03/12/2020", "13/02/2019", "07/07/2022",

"13/07/2017"), DETAILS_study_period_start_date = c("01/01/2008",

"01/01/2008", "01/04/2020", "01/04/2020", "01/10/2007", "05/07/2009",

"01/10/2011", "01/03/2021", "01/09/2020", "01/01/2011", "01/12/2016",

"01/01/2019", "01/01/1993", "01/04/2010", "01/04/2009", "01/12/2012",

"01/04/2020", "01/01/2013", "02/09/2019", "09/03/2020", "05/04/2010",

"01/11/2012", "11/04/2010", "01/02/2010", "07/03/2020", "29/03/2020",

"01/06/2008", "01/03/2010", "18/05/2009", "24/07/2009", "24/07/2009",

"01/05/2009", "01/05/2013", "01/05/2013", "01/06/2009", "01/02/2021",

"01/10/2002", "01/10/2006", "01/10/2006", "01/10/2010", "01/10/2010",

"29/12/2019", "01/08/2009", "01/12/2011", "01/03/2020", "01/01/2009",

"01/01/2016", "01/07/2009", "22/12/2015", "22/12/2015", "01/01/2011",

"01/12/2013", "01/01/2003", "01/01/2003", "01/02/2009", "01/01/2020",

"10/06/2020", "01/01/2012", "01/01/2012", "01/01/2012", "01/05/2009",

"01/01/2007", "01/01/2012", "11/04/2010", "01/01/2017", "10/12/2018",

"01/01/2013", "01/01/2018", "01/01/2013", "01/10/2013", "01/01/2010",

"01/10/1997", "01/02/2007", "01/12/2002", "01/01/2007", "01/01/2007",

"01/12/2009", "01/02/2007", "01/01/2008", "28/04/2009", "01/01/2020",

"01/11/2007", "01/02/2009", "01/04/2009", "01/01/2005", "01/12/2012",

"03/05/2009", "15/03/2009", "01/04/2008", "01/10/2009", "01/10/2009",

"01/02/2012", "01/02/2012", "01/06/2009", "19/04/2009", "27/02/2020",

"01/10/2011", "01/09/2020", "01/02/2013", "01/12/2011", "15/11/2008",

"01/01/2011", "01/04/2020", "01/04/2020", "01/05/2012", "01/05/2012",

"01/02/2010", "01/03/2020", "01/01/2022", "15/05/2023", "29/09/2015",

"01/01/2011", "01/07/2001", "01/01/2009", "01/01/2014", "01/03/2007",

"01/02/2009", "19/02/2020", "01/11/2007", "13/01/2020", "01/10/2013",

"01/10/2010", "01/11/2007", "01/01/2009", "01/04/2009", "29/06/2009",

"01/01/2008", "01/01/2012", "01/01/2012", "03/11/2013", "01/04/2020",

"01/05/2009", "01/06/2009", "05/07/2009", "05/04/2010", "01/01/2020",

"01/01/2016", "06/03/2017", "01/02/2007"), DETAILS_study_period_end_date = c("31/12/2009",

"31/12/2009", "31/12/2020", "31/08/2020", "31/05/2011", "04/10/2009",

"07/05/2014", "31/03/2022", "31/03/2021", "01/01/2012", "31/08/2021",

"31/12/2021", "01/01/2008", "31/03/2011", "31/12/2009", "31/03/2018",

"31/10/2020", "31/12/2020", "23/05/2020", "11/08/2020", "08/04/2012",

"31/03/2015", "10/04/2011", "01/02/2014", "20/07/2020", "27/08/2022",

"31/12/2012", "31/03/2013", "28/02/2010", "05/09/2010", "05/09/2010",

"31/12/2010", "31/12/2016", "31/12/2016", "31/10/2013", "30/11/2021",

"31/05/2009", "31/12/2011", "31/12/2011", "01/06/2015", "01/06/2015",

"31/12/2020", "31/12/2009", "10/02/2012", "31/10/2021", "31/12/2017",

"31/12/2019", "31/12/2013", "22/09/2019", "22/09/2019", "31/12/2013",

"30/06/2014", "31/12/2019", "31/12/2019", "31/12/2012", "30/04/2022",

"31/08/2020", "15/05/2016", "31/12/2017", "31/12/2017", "31/03/2010",

"31/12/2016", "31/12/2015", "10/04/2014", "30/04/2018", "09/12/2019",

"01/01/2018", "31/12/2021", "31/05/2013", "30/09/2017", "31/12/2016",

"31/03/2010", "30/03/2011", "31/01/2005", "31/12/2011", "31/12/2017",

"30/04/2015", "30/06/2013", "01/02/2014", "31/12/2009", "20/08/2020",

"01/11/2014", "31/12/2013", "31/12/2009", "31/12/2010", "31/03/2015",

"31/07/2009", "30/10/2009", "31/05/2009", "31/12/2009", "31/12/2009",

"01/02/2014", "01/02/2014", "30/09/2010", "21/08/2009", "08/06/2020",

"31/03/2012", "30/04/2021", "31/08/2019", "10/02/2012", "30/04/2010",

"31/12/2014", "31/03/2022", "31/03/2022", "30/04/2015", "30/04/2015",

"31/05/2011", "31/12/2020", "31/12/2023", "21/05/2023", "15/03/2017",

"31/12/2016", "29/06/2013", "31/12/2012", "31/12/2018", "28/02/2011",

"31/12/2011", "08/04/2020", "31/03/2009", "27/06/2020", "31/01/2014",

"30/04/2018", "31/12/2011", "31/12/2012", "31/12/2010", "27/12/2009",

"30/06/2013", "31/12/2016", "31/12/2016", "26/01/2014", "31/01/2021",

"31/12/2016", "31/10/2009", "18/07/2009", "08/04/2012", "30/04/2020",

"31/08/2017", "13/03/2021", "28/02/2014"), DETAILS_geographical_scope = c("Regional",

"Regional", "Regional", "Regional", "Regional", "National", "National",

"National", "National", "National", "National", "National", "National",

"Regional", "National", "National", "Regional", "National", "Multinational",

"Regional", "Regional", "National", "Regional", "Regional", "Regional",

"National", "Regional", "Regional", "National", "National", "National",

"Regional", "Regional", "Regional", "Regional", "National", "Regional",

"National", "National", "National", "National", "National", "National",

"National", "National", "Regional", "National", "Regional", "Regional",

"Regional", "Regional", "Multinational", "Regional", "Regional",

"National", "National", "National", "National", "National", "National",

"Regional", "National", "National", "Regional", "Regional", "Regional",

"Regional", "National", "Multinational", "Regional", "National",

"National", "Regional", "Regional", "Regional", "National", "Regional",

"Regional", "National", "Regional", "Regional", "National", "National",

"National", "National", "National", "Regional", "Regional", "Regional",

"Multinational", "Multinational", "National", "National", "Regional",

"National", "National", "National", "Regional", "Regional", "National",

"Multinational", "Regional", "Regional", "Regional", "Regional",

"Regional", "Regional", "National", "National", "Regional", "National",

"National", "Regional", "Multinational", "National", "Regional",

"National", "National", "Regional", "National", "National", "Regional",

"Regional", "Multinational", "Regional", "National", "National",

"National", "National", "Multinational", "Regional", "National",

"National", "National", "Regional", "National", "Multinational",

"National", "Multinational"), DETAILS_which_country = c("Guatemala",

"Guatemala", "Mexico", "India", "USA", "La Reunion island", "Korea",

"Sierra Leone", "Canada", "Australia", "USA", "Germany", "Slovak Republic",

"UK", "Mexico", "France", "India", "Brazil", "Brazil, Canada, China, France, India, Ivory Coast, Kenya, Lebanon, Mexico, Nepal, Peru, Romania, Russia, Serbia, Spain, Turkey, Ukraine",

"France", "China", "France", "Mexico", "Egypt", "India", "Mexico",

"Egypt", "Jordan", "Greece", "Japan", "Japan", "India", "USA",

"USA", "China", "Malta", "USA", "England", "England", "Uganda",

"Uganda", "Brazil", "Mexico", "Mexico", "Sierra Leone", "India",

"Egypt", "Vietnam", "Iran", "Iran", "Malawi", "Russia, Turkey, China, Spain",

"Brazil", "Brazil", "South Africa", "Egypt", "Bangladesh", "Germany",

"Australia", "Australia", "USA", "Netherlands", "Oman", "Mexico",

"USA", "Zambia", "Brazil", "Bahrain", "Russia, Turkey, France, Spain",

"USA", "Saudi Arabia", "France", "Vietnam", "Greece", "China",

"Singapore", "Germany", "Vietnam", "Jordan", "Brazil", "Brazil",

"Egypt", "South Africa", "Argentina", "Taiwan", "France", "USA",

"Mexico", "Chile", "Argentina, Australia, Austria, Belgium, Chile, China, Denmark, Estonia, Germany, Greece, Norway, Peru, Poland, Spain, Thailand, United Kingdom, United States",

"Argentina, Australia, Austria, Belgium, Chile, China, Denmark, Estonia, Germany, Greece, Norway, Peru, Poland, Spain, Thailand, United Kingdom, United States",

"Egypt", "Egypt", "Serbia", "Brazil", "Nigeria", "Canada", "Brazil",

"Colombia", "Mexico", "Belgium, Canada, Czech Rep., Estonia, France, Germany, Mexico, Norway, Poland, Romania, Russia, Taiwan, Netherlands, UK, USA",

"Malawi", "South Africa", "South Africa", "South Africa", "South Africa",

"Egypt", "Bangladesh", "New Zealand", "Ireland", "Georgia", "Yemen",

"USA", "Dem. Rep. Congo, Kenya, Madagascar, Malawi, Rwanda, South Africa, Tanzania, Uganda",

"Kenya", "Kenya", "South Africa", "Iran", "Netherlands", "Mexico",

"Mexico", "Italy", "Guatemala", "Albania, Armenia, Belarus, Georgia, Kazakhstan, Kyrgyzstan, Romania, Russia, Ukraine",

"Italy", "Iceland", "Oman", "Kenya", "Kenya", "Austria, Belgium, Czech Republic, Croatia, Denmark, Finland, France, Georgia, Germany, Greece, Ireland, Italy, Lithuania, Macedonia, Netherlands, Poland, Portugal, Romania, Slovenia, Spain, United Kingdom, Iran, Qatar, Saudi Arabia, Turkey, United Arab Emirates, United States, Argentina, Brazil, Chile, Colombia, Ecuador, Mexico, Peru, Venezuela, Australia, China, Philippines, Vietnam, India, Pakistan, Sri Lanka",

"Brazil", "Ecuador", "Mexico", "UK", "China", "Mexico", "Canada, USA, Mexico, UK, France, Finland, Saudi Arabia, Malawi, Kenya, India, China, Korea, Vietnam, Cambodia, Australia, NZ, Madagascar, Ireland",

"Germany", "Egypt, Jordan, Oman, Qatar, Yemen"), DETAILS_number_of_countries = c(NA,

NA, NA, NA, NA, NA, NA, NA, NA, NA, NA, NA, NA, NA, NA, NA, NA,

NA, 17L, NA, NA, NA, NA, NA, NA, NA, NA, NA, NA, NA, NA, NA,

NA, NA, NA, NA, NA, NA, NA, NA, NA, NA, NA, NA, NA, NA, NA, NA,

NA, NA, NA, 4L, NA, NA, NA, NA, NA, NA, NA, NA, NA, NA, NA, NA,

NA, NA, NA, NA, 4L, NA, NA, NA, NA, NA, NA, NA, NA, NA, NA, NA,

NA, NA, NA, NA, NA, NA, NA, NA, NA, 17L, 17L, NA, NA, NA, NA,

NA, NA, NA, NA, NA, 15L, NA, NA, NA, NA, NA, NA, NA, NA, NA,

NA, NA, NA, 8L, NA, NA, NA, NA, NA, NA, NA, NA, NA, 11L, NA,

NA, NA, NA, NA, 42L, NA, NA, NA, NA, NA, NA, 20L, NA, 5L), POPULATION_case_type = c("ili",

"severe_pneumonia", "sus_covid", "sus_covid", "ari", "ari", "ari",

"sus_covid", "sus_covid", "ili", "ari", "sari_sus_covid", "ari_ili",

"ari", "ili", "ili", "sus_covid", "sari", "ili", "sus_covid",

"sari", "ili", "ili", "sari", "sari", "sus_covid", "ari", "ari",

"ili", "ili", "ili", "ili", "ari_ili", "sari", "ari", "sari",

"ari", "ari", "ili", "ili", "sari", "sari", "ari", "sari", "sus_covid",

"ili", "sari", "sari", "ili", "sari", "sari", "ili", "ari", "sari",

"sari", "ili_sari", "sus_covid", "sari", "ili", "ili", "ari",

"sari", "sari", "ili", "ili", "ili", "sari", "sari", "ili", "ari",

"ili", "ili", "ari", "ari", "ili", "ili", "ili", "ari", "sari",

"ari_ili", "sus_covid", "sari", "sari", "ili", "ili", "ili",

"ili", "ili", "sari", "ili", "ili", "sari", "sari", "sari", "ili",

"sus_covid", "ari", "sus_covid", "ari", "ari", "ili", "sari",

"ili_sus_covid", "sari_sus_covid", "sari", "ili", "sari", "sari",

"sari", "sari", "sari", "sari", "maari", "sari", "sari", "sari",

"sari", "sari_sus_covid", "ari", "sus_covid", "sari", "ili",

"ari", "sari", "ili", "ili", "sari", "ili", "sari", "sari", "sari_sus_covid",

"ili_sari", "ili_sari", "ili", "sari", "sus_covid", "sari", "ari",

"sari"), SETTING_type = c("treatment_seeking", "hospitalised",

"treatment_seeking", "treatment_seeking", "treatment_seeking_hospitalised",

"treatment_seeking_hospitalised", "treatment_seeking", "hospitalised",

"hospitalised", "non_treatment_seeking", "treatment_seeking_hospitalised",

"hospitalised", "treatment_seeking", "hospitalised", "treatment_seeking_hospitalised",

"hospitalised", "non_treatment_seeking", "hospitalised", "hospitalised",

"treatment_seeking", "hospitalised", "hospitalised", "treatment_seeking",

"hospitalised", "hospitalised", "treatment_seeking_hospitalised",

"hospitalised", "hospitalised", "treatment_seeking", "treatment_seeking",

"hospitalised", "treatment_seeking_hospitalised", "treatment_seeking",

"hospitalised", "hospitalised", "hospitalised", "hospitalised",

"non_treatment_seeking", "non_treatment_seeking", "treatment_seeking",

"hospitalised", "hospitalised", "hospitalised", "hospitalised",

"unknown", "hospitalised", "hospitalised", "hospitalised", "treatment_seeking",

"hospitalised", "treatment_seeking", "hospitalised", "treatment_seeking",

"treatment_seeking", "hospitalised", "treatment_seeking_hospitalised",

"treatment_seeking_hospitalised", "hospitalised", "treatment_seeking",

"non_treatment_seeking", "hospitalised", "icu", "hospitalised",

"treatment_seeking", "treatment_seeking", "treatment_seeking",

"treatment_seeking", "hospitalised", "hospitalised", "non_treatment_seeking",

"hospitalised", "treatment_seeking", "hospitalised", "hospitalised",

"treatment_seeking", "treatment_seeking", "treatment_seeking",

"hospitalised", "hospitalised", "treatment_seeking", "unknown",

"hospitalised", "hospitalised", "non_treatment_seeking", "unknown",

"hospitalised", "hospitalised", "unknown", "treatment_seeking",

"treatment_seeking", "hospitalised", "hospitalised", "icu", "hospitalised",

"hospitalised", "unknown", "hospitalised", "treatment_seeking_hospitalised",

"treatment_seeking_hospitalised", "hospitalised", "non_treatment_seeking",

"treatment_seeking", "treatment_seeking", "hospitalised", "hospitalised",

"treatment_seeking", "hospitalised", "hospitalised", "hospitalised",

"hospitalised", "hospitalised", "hospitalised", "hospitalised",

"hospitalised", "hospitalised", "treatment_seeking", "hospitalised",

"hospitalised", "treatment_seeking", "unknown", "hospitalised",

"hospitalised", "hospitalised", "hospitalised", "hospitalised",

"treatment_seeking_hospitalised", "hospitalised", "treatment_seeking",

"hospitalised", "icu", "hospitalised", "treatment_seeking_hospitalised",

"treatment_seeking_hospitalised", "treatment_seeking_hospitalised",

"hospitalised", "treatment_seeking_hospitalised", "icu", "treatment_seeking",

"hospitalised"), OUTCOMES_L1_symptoms = c(TRUE, TRUE, TRUE, TRUE,

FALSE, TRUE, FALSE, FALSE, FALSE, FALSE, FALSE, FALSE, FALSE,

FALSE, FALSE, TRUE, TRUE, FALSE, FALSE, TRUE, TRUE, TRUE, TRUE,

TRUE, TRUE, FALSE, FALSE, TRUE, FALSE, FALSE, FALSE, TRUE, TRUE,

TRUE, TRUE, TRUE, FALSE, TRUE, TRUE, TRUE, TRUE, FALSE, TRUE,

FALSE, FALSE, TRUE, FALSE, FALSE, TRUE, TRUE, FALSE, FALSE, TRUE,

TRUE, FALSE, FALSE, TRUE, FALSE, FALSE, FALSE, TRUE, FALSE, FALSE,

TRUE, TRUE, TRUE, FALSE, FALSE, FALSE, TRUE, FALSE, FALSE, FALSE,

TRUE, FALSE, FALSE, FALSE, TRUE, FALSE, TRUE, FALSE, FALSE, FALSE,

FALSE, FALSE, TRUE, FALSE, TRUE, TRUE, FALSE, FALSE, TRUE, TRUE,

TRUE, FALSE, TRUE, FALSE, TRUE, FALSE, FALSE, TRUE, TRUE, FALSE,

TRUE, FALSE, FALSE, TRUE, TRUE, FALSE, TRUE, TRUE, FALSE, FALSE,

FALSE, FALSE, TRUE, FALSE, FALSE, TRUE, FALSE, FALSE, FALSE,

TRUE, FALSE, FALSE, FALSE, FALSE, TRUE, TRUE, TRUE, TRUE, FALSE,

FALSE, TRUE, TRUE, FALSE, FALSE, FALSE, FALSE), OUTCOMES_L1_hsb = c(FALSE,

FALSE, FALSE, FALSE, FALSE, FALSE, FALSE, FALSE, FALSE, FALSE,

FALSE, FALSE, FALSE, FALSE, FALSE, FALSE, FALSE, FALSE, FALSE,

FALSE, FALSE, FALSE, FALSE, FALSE, FALSE, FALSE, FALSE, FALSE,

FALSE, FALSE, FALSE, FALSE, FALSE, FALSE, FALSE, FALSE, FALSE,

FALSE, FALSE, FALSE, FALSE, FALSE, FALSE, FALSE, FALSE, FALSE,

FALSE, FALSE, FALSE, FALSE, FALSE, FALSE, FALSE, FALSE, FALSE,

FALSE, FALSE, FALSE, FALSE, FALSE, FALSE, FALSE, FALSE, FALSE,

TRUE, FALSE, FALSE, FALSE, FALSE, TRUE, FALSE, FALSE, FALSE,

FALSE, FALSE, FALSE, FALSE, FALSE, FALSE, FALSE, FALSE, FALSE,

FALSE, TRUE, FALSE, FALSE, FALSE, FALSE, FALSE, FALSE, FALSE,

FALSE, FALSE, FALSE, FALSE, FALSE, FALSE, FALSE, FALSE, FALSE,

FALSE, FALSE, FALSE, FALSE, FALSE, FALSE, FALSE, FALSE, FALSE,

FALSE, FALSE, FALSE, FALSE, FALSE, FALSE, FALSE, FALSE, FALSE,

FALSE, FALSE, FALSE, FALSE, FALSE, FALSE, FALSE, FALSE, FALSE,

FALSE, FALSE, FALSE, FALSE, FALSE, FALSE, FALSE, FALSE, FALSE,

FALSE, FALSE, FALSE), OUTCOMES_L1_absence = c(FALSE, FALSE, FALSE,

FALSE, FALSE, FALSE, FALSE, FALSE, FALSE, TRUE, FALSE, FALSE,

FALSE, FALSE, FALSE, FALSE, FALSE, FALSE, FALSE, FALSE, FALSE,

FALSE, FALSE, FALSE, FALSE, FALSE, FALSE, FALSE, FALSE, FALSE,

FALSE, FALSE, FALSE, FALSE, FALSE, FALSE, FALSE, TRUE, TRUE,

FALSE, FALSE, FALSE, FALSE, FALSE, FALSE, FALSE, FALSE, FALSE,

FALSE, FALSE, FALSE, FALSE, FALSE, FALSE, FALSE, FALSE, FALSE,

FALSE, FALSE, TRUE, FALSE, FALSE, FALSE, FALSE, TRUE, FALSE,

FALSE, FALSE, FALSE, TRUE, FALSE, FALSE, FALSE, FALSE, FALSE,

FALSE, FALSE, FALSE, FALSE, FALSE, FALSE, FALSE, FALSE, FALSE,

FALSE, FALSE, FALSE, FALSE, FALSE, FALSE, FALSE, FALSE, FALSE,

FALSE, FALSE, FALSE, FALSE, FALSE, FALSE, FALSE, FALSE, FALSE,

FALSE, FALSE, FALSE, FALSE, FALSE, FALSE, FALSE, FALSE, FALSE,

FALSE, FALSE, FALSE, FALSE, FALSE, FALSE, FALSE, FALSE, FALSE,

FALSE, FALSE, FALSE, FALSE, FALSE, FALSE, FALSE, FALSE, FALSE,

FALSE, FALSE, FALSE, FALSE, TRUE, FALSE, FALSE, FALSE, FALSE,

FALSE), OUTCOMES_L1_signs = c(TRUE, TRUE, FALSE, TRUE, FALSE,

FALSE, FALSE, FALSE, FALSE, FALSE, FALSE, FALSE, FALSE, FALSE,

FALSE, FALSE, TRUE, FALSE, FALSE, FALSE, TRUE, FALSE, FALSE,

TRUE, FALSE, FALSE, FALSE, TRUE, FALSE, FALSE, FALSE, FALSE,

FALSE, FALSE, FALSE, FALSE, FALSE, FALSE, FALSE, TRUE, TRUE,

FALSE, TRUE, FALSE, FALSE, TRUE, FALSE, TRUE, TRUE, TRUE, FALSE,

FALSE, TRUE, TRUE, FALSE, FALSE, TRUE, FALSE, FALSE, FALSE, TRUE,

FALSE, FALSE, FALSE, TRUE, TRUE, FALSE, FALSE, FALSE, FALSE,

FALSE, FALSE, TRUE, TRUE, FALSE, FALSE, FALSE, TRUE, FALSE, TRUE,

FALSE, FALSE, FALSE, FALSE, FALSE, FALSE, FALSE, TRUE, TRUE,

FALSE, FALSE, TRUE, TRUE, FALSE, FALSE, FALSE, FALSE, FALSE,

FALSE, FALSE, FALSE, TRUE, FALSE, FALSE, FALSE, FALSE, TRUE,

TRUE, FALSE, FALSE, FALSE, FALSE, FALSE, FALSE, TRUE, TRUE, FALSE,

TRUE, TRUE, FALSE, FALSE, FALSE, TRUE, FALSE, FALSE, FALSE, FALSE,

TRUE, TRUE, FALSE, FALSE, FALSE, FALSE, FALSE, TRUE, FALSE, FALSE,

FALSE, FALSE), OUTCOMES_L1_score = c(FALSE, FALSE, TRUE, TRUE,

TRUE, FALSE, FALSE, TRUE, FALSE, FALSE, FALSE, FALSE, FALSE,

TRUE, FALSE, FALSE, TRUE, FALSE, FALSE, TRUE, FALSE, FALSE, FALSE,

FALSE, FALSE, FALSE, FALSE, FALSE, FALSE, FALSE, FALSE, FALSE,

FALSE, FALSE, FALSE, FALSE, FALSE, FALSE, FALSE, FALSE, FALSE,

FALSE, FALSE, FALSE, FALSE, FALSE, FALSE, FALSE, FALSE, FALSE,

TRUE, FALSE, FALSE, FALSE, FALSE, FALSE, FALSE, FALSE, FALSE,

FALSE, FALSE, TRUE, FALSE, FALSE, TRUE, TRUE, FALSE, FALSE, FALSE,

FALSE, FALSE, FALSE, FALSE, FALSE, FALSE, FALSE, TRUE, FALSE,

FALSE, FALSE, FALSE, FALSE, FALSE, FALSE, FALSE, FALSE, TRUE,

FALSE, FALSE, TRUE, TRUE, FALSE, FALSE, FALSE, FALSE, FALSE,

TRUE, TRUE, FALSE, FALSE, TRUE, TRUE, FALSE, FALSE, FALSE, FALSE,

FALSE, FALSE, FALSE, FALSE, FALSE, FALSE, FALSE, FALSE, FALSE,

FALSE, FALSE, TRUE, TRUE, FALSE, FALSE, FALSE, FALSE, FALSE,

FALSE, FALSE, FALSE, FALSE, FALSE, TRUE, FALSE, FALSE, FALSE,

FALSE, FALSE, FALSE, TRUE, FALSE, TRUE), OUTCOMES_L1_ix = c(FALSE,

FALSE, FALSE, FALSE, FALSE, FALSE, FALSE, FALSE, FALSE, FALSE,

FALSE, FALSE, FALSE, FALSE, FALSE, FALSE, TRUE, FALSE, FALSE,

FALSE, TRUE, FALSE, FALSE, FALSE, FALSE, FALSE, FALSE, FALSE,

FALSE, FALSE, FALSE, FALSE, FALSE, FALSE, FALSE, FALSE, TRUE,

FALSE, FALSE, FALSE, FALSE, FALSE, FALSE, FALSE, FALSE, FALSE,

FALSE, FALSE, FALSE, FALSE, FALSE, TRUE, FALSE, FALSE, FALSE,

FALSE, FALSE, FALSE, FALSE, FALSE, TRUE, FALSE, FALSE, TRUE,

FALSE, FALSE, FALSE, FALSE, FALSE, FALSE, FALSE, FALSE, TRUE,

FALSE, FALSE, FALSE, FALSE, TRUE, FALSE, FALSE, FALSE, FALSE,

FALSE, FALSE, FALSE, FALSE, TRUE, TRUE, TRUE, FALSE, FALSE, TRUE,

TRUE, FALSE, FALSE, FALSE, FALSE, FALSE, FALSE, FALSE, FALSE,

FALSE, FALSE, FALSE, FALSE, FALSE, TRUE, FALSE, FALSE, FALSE,

FALSE, FALSE, FALSE, FALSE, FALSE, FALSE, FALSE, FALSE, TRUE,

FALSE, FALSE, FALSE, TRUE, FALSE, FALSE, FALSE, FALSE, FALSE,

FALSE, FALSE, FALSE, FALSE, FALSE, FALSE, TRUE, FALSE, FALSE,

FALSE, FALSE), OUTCOMES_L1_complications = c(FALSE, FALSE, FALSE,

FALSE, FALSE, TRUE, FALSE, FALSE, FALSE, FALSE, FALSE, FALSE,

FALSE, FALSE, FALSE, TRUE, TRUE, FALSE, FALSE, FALSE, TRUE, TRUE,

FALSE, TRUE, FALSE, FALSE, FALSE, TRUE, FALSE, TRUE, TRUE, FALSE,

FALSE, FALSE, FALSE, TRUE, FALSE, FALSE, FALSE, FALSE, FALSE,

FALSE, FALSE, FALSE, FALSE, FALSE, FALSE, FALSE, TRUE, TRUE,

FALSE, TRUE, FALSE, FALSE, FALSE, FALSE, FALSE, FALSE, FALSE,

FALSE, FALSE, FALSE, FALSE, FALSE, TRUE, TRUE, FALSE, FALSE,

FALSE, FALSE, FALSE, FALSE, FALSE, TRUE, FALSE, FALSE, FALSE,

FALSE, FALSE, FALSE, FALSE, FALSE, FALSE, FALSE, FALSE, TRUE,

TRUE, FALSE, FALSE, TRUE, TRUE, TRUE, TRUE, FALSE, FALSE, FALSE,

FALSE, FALSE, TRUE, FALSE, FALSE, FALSE, FALSE, FALSE, FALSE,

FALSE, FALSE, FALSE, FALSE, TRUE, FALSE, FALSE, FALSE, FALSE,

FALSE, FALSE, FALSE, FALSE, FALSE, FALSE, FALSE, TRUE, FALSE,

FALSE, FALSE, FALSE, FALSE, FALSE, FALSE, TRUE, FALSE, FALSE,

FALSE, FALSE, FALSE, FALSE, FALSE, FALSE, FALSE), OUTCOMES_L1_treatment = c(FALSE,

FALSE, FALSE, FALSE, FALSE, TRUE, FALSE, TRUE, FALSE, FALSE,

FALSE, FALSE, FALSE, FALSE, FALSE, FALSE, FALSE, FALSE, FALSE,

FALSE, TRUE, FALSE, TRUE, FALSE, FALSE, FALSE, FALSE, FALSE,

FALSE, FALSE, FALSE, TRUE, TRUE, TRUE, FALSE, FALSE, FALSE, FALSE,

FALSE, FALSE, FALSE, TRUE, TRUE, FALSE, FALSE, FALSE, FALSE,

TRUE, TRUE, TRUE, FALSE, FALSE, FALSE, FALSE, TRUE, FALSE, FALSE,

FALSE, FALSE, FALSE, FALSE, FALSE, FALSE, TRUE, TRUE, TRUE, TRUE,

FALSE, FALSE, FALSE, FALSE, FALSE, TRUE, TRUE, FALSE, FALSE,

TRUE, TRUE, FALSE, TRUE, FALSE, FALSE, TRUE, FALSE, FALSE, FALSE,

TRUE, TRUE, TRUE, TRUE, TRUE, FALSE, FALSE, FALSE, FALSE, FALSE,

FALSE, FALSE, FALSE, FALSE, FALSE, FALSE, FALSE, FALSE, FALSE,

FALSE, FALSE, FALSE, FALSE, FALSE, TRUE, FALSE, FALSE, FALSE,

FALSE, FALSE, TRUE, FALSE, TRUE, FALSE, FALSE, FALSE, TRUE, TRUE,

FALSE, FALSE, TRUE, FALSE, FALSE, TRUE, FALSE, FALSE, TRUE, TRUE,

FALSE, FALSE, FALSE, FALSE, FALSE), OUTCOMES_L1_hospital = c(FALSE,

FALSE, FALSE, TRUE, FALSE, TRUE, TRUE, TRUE, TRUE, TRUE, TRUE,

FALSE, FALSE, TRUE, TRUE, TRUE, TRUE, FALSE, TRUE, TRUE, TRUE,

TRUE, TRUE, FALSE, TRUE, TRUE, FALSE, TRUE, TRUE, TRUE, TRUE,

FALSE, TRUE, FALSE, FALSE, FALSE, TRUE, FALSE, FALSE, FALSE,

TRUE, FALSE, TRUE, FALSE, FALSE, FALSE, FALSE, TRUE, TRUE, FALSE,

FALSE, TRUE, FALSE, TRUE, TRUE, TRUE, FALSE, TRUE, TRUE, FALSE,

TRUE, FALSE, FALSE, TRUE, TRUE, TRUE, TRUE, FALSE, TRUE, FALSE,

FALSE, TRUE, TRUE, TRUE, TRUE, TRUE, TRUE, TRUE, FALSE, TRUE,

FALSE, FALSE, TRUE, TRUE, FALSE, TRUE, TRUE, TRUE, TRUE, TRUE,

TRUE, FALSE, FALSE, TRUE, FALSE, FALSE, TRUE, TRUE, TRUE, FALSE,

TRUE, FALSE, TRUE, TRUE, TRUE, TRUE, TRUE, FALSE, FALSE, TRUE,

TRUE, FALSE, FALSE, FALSE, TRUE, FALSE, TRUE, FALSE, TRUE, FALSE,

FALSE, FALSE, TRUE, TRUE, FALSE, FALSE, TRUE, FALSE, TRUE, FALSE,

TRUE, FALSE, TRUE, TRUE, TRUE, TRUE, FALSE, TRUE, FALSE), OUTCOMES_L1_icu = c(FALSE,

TRUE, FALSE, TRUE, FALSE, TRUE, FALSE, FALSE, TRUE, FALSE, FALSE,

TRUE, FALSE, TRUE, FALSE, TRUE, TRUE, TRUE, TRUE, FALSE, TRUE,

FALSE, FALSE, TRUE, TRUE, TRUE, TRUE, TRUE, FALSE, FALSE, TRUE,

TRUE, FALSE, TRUE, TRUE, TRUE, TRUE, FALSE, FALSE, FALSE, FALSE,

TRUE, FALSE, FALSE, FALSE, FALSE, FALSE, TRUE, FALSE, FALSE,

FALSE, TRUE, FALSE, FALSE, TRUE, TRUE, TRUE, TRUE, FALSE, FALSE,

TRUE, FALSE, FALSE, TRUE, FALSE, FALSE, TRUE, TRUE, TRUE, FALSE,

TRUE, FALSE, TRUE, FALSE, FALSE, FALSE, FALSE, FALSE, TRUE, FALSE,

FALSE, TRUE, TRUE, FALSE, FALSE, TRUE, TRUE, FALSE, TRUE, FALSE,

TRUE, TRUE, TRUE, FALSE, FALSE, FALSE, TRUE, TRUE, TRUE, FALSE,

FALSE, FALSE, FALSE, TRUE, TRUE, FALSE, TRUE, FALSE, TRUE, TRUE,

TRUE, TRUE, TRUE, FALSE, TRUE, FALSE, TRUE, TRUE, FALSE, TRUE,

FALSE, TRUE, TRUE, TRUE, TRUE, FALSE, TRUE, FALSE, FALSE, TRUE,

TRUE, FALSE, TRUE, FALSE, TRUE, TRUE, TRUE, FALSE, TRUE), OUTCOMES_L1_death = c(FALSE,

TRUE, FALSE, TRUE, FALSE, TRUE, FALSE, FALSE, TRUE, FALSE, FALSE,

TRUE, TRUE, TRUE, TRUE, TRUE, TRUE, TRUE, TRUE, TRUE, TRUE, TRUE,

TRUE, TRUE, TRUE, TRUE, TRUE, FALSE, FALSE, FALSE, TRUE, TRUE,

FALSE, TRUE, FALSE, TRUE, FALSE, FALSE, FALSE, FALSE, TRUE, TRUE,

TRUE, TRUE, TRUE, TRUE, TRUE, TRUE, TRUE, TRUE, FALSE, TRUE,

FALSE, TRUE, TRUE, TRUE, TRUE, TRUE, FALSE, FALSE, TRUE, TRUE,

TRUE, TRUE, FALSE, TRUE, TRUE, TRUE, TRUE, FALSE, TRUE, FALSE,

FALSE, FALSE, TRUE, FALSE, FALSE, FALSE, TRUE, TRUE, TRUE, TRUE,

TRUE, FALSE, TRUE, TRUE, TRUE, TRUE, TRUE, TRUE, TRUE, TRUE,

TRUE, TRUE, TRUE, TRUE, TRUE, FALSE, TRUE, TRUE, TRUE, FALSE,

FALSE, TRUE, TRUE, FALSE, TRUE, TRUE, FALSE, TRUE, TRUE, TRUE,

TRUE, TRUE, TRUE, TRUE, TRUE, TRUE, FALSE, TRUE, TRUE, FALSE,

TRUE, TRUE, TRUE, TRUE, TRUE, FALSE, TRUE, TRUE, TRUE, TRUE,

TRUE, FALSE, TRUE, TRUE, TRUE, FALSE, TRUE), OUTCOMES_L2_SYMPTOM_abdominal_pain = c(FALSE,

FALSE, FALSE, FALSE, FALSE, TRUE, FALSE, FALSE, FALSE, FALSE,

FALSE, FALSE, FALSE, FALSE, FALSE, FALSE, TRUE, FALSE, FALSE,

FALSE, TRUE, FALSE, FALSE, FALSE, FALSE, FALSE, FALSE, FALSE,

FALSE, FALSE, FALSE, FALSE, FALSE, FALSE, FALSE, FALSE, FALSE,

FALSE, FALSE, FALSE, FALSE, FALSE, TRUE, FALSE, FALSE, FALSE,

FALSE, FALSE, FALSE, FALSE, FALSE, FALSE, TRUE, TRUE, FALSE,

FALSE, TRUE, FALSE, FALSE, FALSE, FALSE, FALSE, FALSE, FALSE,

FALSE, FALSE, FALSE, FALSE, FALSE, FALSE, FALSE, FALSE, FALSE,

FALSE, FALSE, FALSE, FALSE, FALSE, FALSE, FALSE, FALSE, FALSE,

FALSE, FALSE, FALSE, FALSE, FALSE, FALSE, FALSE, FALSE, FALSE,

FALSE, FALSE, FALSE, FALSE, TRUE, FALSE, TRUE, FALSE, FALSE,

FALSE, FALSE, FALSE, FALSE, FALSE, FALSE, FALSE, FALSE, FALSE,

FALSE, FALSE, FALSE, FALSE, FALSE, FALSE, FALSE, FALSE, FALSE,

FALSE, FALSE, FALSE, FALSE, FALSE, FALSE, FALSE, FALSE, FALSE,

FALSE, FALSE, FALSE, FALSE, FALSE, FALSE, FALSE, TRUE, FALSE,

FALSE, FALSE, FALSE), OUTCOMES_L2_SYMPTOM_chest_pain = c(TRUE,

TRUE, TRUE, FALSE, FALSE, FALSE, FALSE, FALSE, FALSE, FALSE,

FALSE, FALSE, FALSE, FALSE, FALSE, FALSE, FALSE, FALSE, FALSE,

TRUE, FALSE, FALSE, FALSE, FALSE, TRUE, FALSE, FALSE, FALSE,

FALSE, FALSE, FALSE, TRUE, FALSE, FALSE, FALSE, FALSE, FALSE,

FALSE, FALSE, FALSE, FALSE, FALSE, TRUE, FALSE, FALSE, FALSE,

FALSE, FALSE, TRUE, TRUE, FALSE, FALSE, TRUE, TRUE, FALSE, FALSE,

FALSE, FALSE, FALSE, FALSE, FALSE, FALSE, FALSE, FALSE, FALSE,

FALSE, FALSE, FALSE, FALSE, FALSE, FALSE, FALSE, FALSE, FALSE,

FALSE, FALSE, FALSE, FALSE, FALSE, FALSE, FALSE, FALSE, FALSE,

FALSE, FALSE, FALSE, FALSE, TRUE, FALSE, FALSE, FALSE, FALSE,

FALSE, FALSE, FALSE, TRUE, FALSE, TRUE, FALSE, FALSE, FALSE,

FALSE, FALSE, FALSE, FALSE, FALSE, FALSE, FALSE, FALSE, FALSE,

FALSE, FALSE, FALSE, FALSE, FALSE, FALSE, FALSE, FALSE, FALSE,

FALSE, FALSE, FALSE, FALSE, FALSE, FALSE, FALSE, FALSE, FALSE,

FALSE, FALSE, FALSE, FALSE, FALSE, FALSE, TRUE, FALSE, FALSE,

FALSE, FALSE), OUTCOMES_L2_SYMPTOM_confusion = c(FALSE, FALSE,

FALSE, FALSE, FALSE, FALSE, FALSE, FALSE, FALSE, FALSE, FALSE,

FALSE, FALSE, FALSE, FALSE, FALSE, FALSE, FALSE, FALSE, FALSE,

FALSE, FALSE, FALSE, FALSE, FALSE, FALSE, FALSE, FALSE, FALSE,

FALSE, FALSE, FALSE, FALSE, FALSE, FALSE, FALSE, FALSE, FALSE,

FALSE, FALSE, FALSE, FALSE, FALSE, FALSE, FALSE, FALSE, FALSE,

FALSE, FALSE, FALSE, FALSE, FALSE, FALSE, FALSE, FALSE, FALSE,

FALSE, FALSE, FALSE, FALSE, FALSE, FALSE, FALSE, TRUE, FALSE,

FALSE, FALSE, FALSE, FALSE, FALSE, FALSE, FALSE, FALSE, FALSE,

FALSE, FALSE, FALSE, FALSE, FALSE, FALSE, FALSE, FALSE, FALSE,

FALSE, FALSE, FALSE, FALSE, FALSE, TRUE, FALSE, FALSE, FALSE,

FALSE, FALSE, FALSE, TRUE, FALSE, FALSE, FALSE, FALSE, FALSE,

FALSE, FALSE, FALSE, FALSE, FALSE, FALSE, FALSE, FALSE, FALSE,

FALSE, FALSE, FALSE, FALSE, FALSE, FALSE, FALSE, FALSE, FALSE,

FALSE, FALSE, FALSE, FALSE, FALSE, FALSE, FALSE, FALSE, FALSE,

FALSE, FALSE, FALSE, FALSE, FALSE, FALSE, FALSE, FALSE, FALSE,

FALSE, FALSE), OUTCOMES_L2_SYMPTOM_convulsions = c(FALSE, FALSE,

FALSE, FALSE, FALSE, FALSE, FALSE, FALSE, FALSE, FALSE, FALSE,

FALSE, FALSE, FALSE, FALSE, FALSE, FALSE, FALSE, FALSE, FALSE,

FALSE, FALSE, FALSE, TRUE, FALSE, FALSE, FALSE, FALSE, FALSE,

FALSE, FALSE, FALSE, TRUE, TRUE, FALSE, FALSE, FALSE, FALSE,

FALSE, FALSE, FALSE, FALSE, FALSE, FALSE, FALSE, FALSE, FALSE,

FALSE, FALSE, FALSE, FALSE, FALSE, FALSE, FALSE, FALSE, FALSE,

FALSE, FALSE, FALSE, FALSE, FALSE, FALSE, FALSE, FALSE, FALSE,

FALSE, FALSE, FALSE, FALSE, FALSE, FALSE, FALSE, FALSE, TRUE,

FALSE, FALSE, FALSE, FALSE, FALSE, FALSE, FALSE, FALSE, FALSE,

FALSE, FALSE, FALSE, FALSE, FALSE, TRUE, FALSE, FALSE, TRUE,

TRUE, FALSE, FALSE, FALSE, FALSE, FALSE, FALSE, FALSE, FALSE,

FALSE, FALSE, FALSE, FALSE, FALSE, TRUE, FALSE, FALSE, FALSE,

FALSE, FALSE, FALSE, FALSE, FALSE, FALSE, FALSE, FALSE, FALSE,

FALSE, FALSE, FALSE, FALSE, FALSE, FALSE, FALSE, FALSE, FALSE,

FALSE, FALSE, FALSE, FALSE, FALSE, FALSE, FALSE, FALSE, FALSE,

FALSE, FALSE), OUTCOMES_L2_SYMPTOM_coryza_rhinorrea_congestion = c(TRUE,

TRUE, TRUE, FALSE, FALSE, TRUE, FALSE, FALSE, FALSE, FALSE, FALSE,

FALSE, FALSE, FALSE, FALSE, FALSE, FALSE, FALSE, FALSE, FALSE,

TRUE, FALSE, FALSE, TRUE, FALSE, FALSE, FALSE, TRUE, FALSE, FALSE,

FALSE, TRUE, TRUE, TRUE, TRUE, FALSE, FALSE, FALSE, FALSE, FALSE,

FALSE, FALSE, TRUE, FALSE, FALSE, TRUE, FALSE, FALSE, FALSE,

FALSE, FALSE, FALSE, TRUE, TRUE, FALSE, FALSE, TRUE, FALSE, FALSE,

FALSE, FALSE, FALSE, FALSE, TRUE, FALSE, FALSE, FALSE, FALSE,

FALSE, TRUE, FALSE, FALSE, FALSE, TRUE, FALSE, FALSE, FALSE,

FALSE, FALSE, TRUE, FALSE, FALSE, FALSE, FALSE, FALSE, FALSE,

FALSE, TRUE, FALSE, FALSE, FALSE, TRUE, TRUE, FALSE, FALSE, FALSE,

FALSE, TRUE, FALSE, FALSE, TRUE, FALSE, FALSE, FALSE, FALSE,

FALSE, FALSE, TRUE, FALSE, FALSE, FALSE, FALSE, FALSE, FALSE,

FALSE, FALSE, FALSE, FALSE, TRUE, FALSE, FALSE, FALSE, FALSE,

FALSE, FALSE, FALSE, FALSE, TRUE, TRUE, FALSE, FALSE, FALSE,

FALSE, FALSE, TRUE, FALSE, FALSE, FALSE, FALSE), OUTCOMES_L2_SYMPTOM_cough = c(TRUE,

TRUE, TRUE, FALSE, FALSE, TRUE, FALSE, FALSE, FALSE, FALSE, FALSE,

FALSE, FALSE, FALSE, FALSE, TRUE, TRUE, FALSE, FALSE, TRUE, TRUE,

TRUE, TRUE, TRUE, TRUE, FALSE, FALSE, TRUE, FALSE, FALSE, FALSE,

FALSE, FALSE, FALSE, TRUE, TRUE, FALSE, FALSE, FALSE, TRUE, TRUE,

FALSE, TRUE, FALSE, FALSE, TRUE, FALSE, FALSE, TRUE, TRUE, FALSE,

FALSE, TRUE, TRUE, FALSE, FALSE, TRUE, FALSE, FALSE, FALSE, TRUE,

FALSE, FALSE, TRUE, FALSE, TRUE, FALSE, FALSE, FALSE, TRUE, FALSE,

FALSE, FALSE, TRUE, FALSE, FALSE, FALSE, FALSE, FALSE, TRUE,

FALSE, FALSE, FALSE, FALSE, FALSE, TRUE, FALSE, TRUE, FALSE,

FALSE, FALSE, TRUE, TRUE, FALSE, FALSE, TRUE, FALSE, TRUE, FALSE,

FALSE, TRUE, FALSE, FALSE, FALSE, FALSE, FALSE, TRUE, FALSE,

FALSE, TRUE, TRUE, FALSE, FALSE, FALSE, FALSE, TRUE, FALSE, FALSE,

TRUE, FALSE, FALSE, FALSE, TRUE, FALSE, FALSE, FALSE, FALSE,

FALSE, FALSE, TRUE, TRUE, FALSE, FALSE, TRUE, TRUE, FALSE, FALSE,

FALSE, FALSE), OUTCOMES_L2_SYMPTOM_productive_cough = c(TRUE,

TRUE, TRUE, FALSE, FALSE, FALSE, FALSE, FALSE, FALSE, FALSE,

FALSE, FALSE, FALSE, FALSE, FALSE, FALSE, FALSE, FALSE, FALSE,

FALSE, TRUE, FALSE, TRUE, TRUE, FALSE, FALSE, FALSE, FALSE, FALSE,

FALSE, FALSE, FALSE, FALSE, FALSE, TRUE, FALSE, FALSE, FALSE,

FALSE, FALSE, FALSE, FALSE, FALSE, FALSE, FALSE, FALSE, FALSE,

FALSE, FALSE, FALSE, FALSE, FALSE, FALSE, FALSE, FALSE, FALSE,

TRUE, FALSE, FALSE, FALSE, FALSE, FALSE, FALSE, TRUE, FALSE,

FALSE, FALSE, FALSE, FALSE, FALSE, FALSE, FALSE, FALSE, FALSE,

FALSE, FALSE, FALSE, FALSE, FALSE, FALSE, FALSE, FALSE, FALSE,

FALSE, FALSE, FALSE, FALSE, FALSE, FALSE, FALSE, FALSE, TRUE,

TRUE, FALSE, FALSE, FALSE, FALSE, FALSE, FALSE, FALSE, FALSE,

FALSE, FALSE, FALSE, FALSE, FALSE, FALSE, FALSE, FALSE, FALSE,

FALSE, FALSE, FALSE, FALSE, FALSE, FALSE, FALSE, FALSE, FALSE,

FALSE, FALSE, FALSE, FALSE, FALSE, FALSE, FALSE, FALSE, FALSE,

FALSE, FALSE, FALSE, FALSE, FALSE, FALSE, TRUE, FALSE, FALSE,

FALSE, FALSE), OUTCOMES_L2_SYMPTOM_diarrhea = c(TRUE, TRUE, TRUE,

TRUE, FALSE, FALSE, FALSE, FALSE, FALSE, FALSE, FALSE, FALSE,

FALSE, FALSE, FALSE, FALSE, TRUE, FALSE, FALSE, TRUE, TRUE, FALSE,

TRUE, FALSE, FALSE, FALSE, FALSE, TRUE, FALSE, FALSE, FALSE,

TRUE, TRUE, TRUE, TRUE, FALSE, FALSE, FALSE, FALSE, TRUE, TRUE,

FALSE, TRUE, FALSE, FALSE, FALSE, FALSE, FALSE, FALSE, FALSE,

FALSE, FALSE, TRUE, TRUE, FALSE, FALSE, TRUE, FALSE, FALSE, FALSE,

FALSE, FALSE, FALSE, TRUE, FALSE, TRUE, FALSE, FALSE, FALSE,

FALSE, FALSE, FALSE, FALSE, FALSE, FALSE, FALSE, FALSE, FALSE,

FALSE, TRUE, FALSE, FALSE, FALSE, FALSE, FALSE, FALSE, FALSE,

FALSE, FALSE, FALSE, FALSE, FALSE, FALSE, FALSE, FALSE, TRUE,

FALSE, FALSE, FALSE, FALSE, FALSE, FALSE, FALSE, FALSE, FALSE,

FALSE, FALSE, FALSE, FALSE, FALSE, FALSE, FALSE, FALSE, FALSE,

FALSE, FALSE, FALSE, FALSE, FALSE, FALSE, FALSE, FALSE, FALSE,

FALSE, FALSE, FALSE, FALSE, FALSE, FALSE, TRUE, TRUE, FALSE,

FALSE, TRUE, TRUE, FALSE, FALSE, FALSE, FALSE), OUTCOMES_L2_SYMPTOM_ear_pain = c(FALSE,

FALSE, FALSE, FALSE, FALSE, FALSE, FALSE, FALSE, FALSE, FALSE,

FALSE, FALSE, FALSE, FALSE, FALSE, FALSE, FALSE, FALSE, FALSE,

FALSE, FALSE, FALSE, FALSE, FALSE, FALSE, FALSE, FALSE, FALSE,

FALSE, FALSE, FALSE, FALSE, FALSE, FALSE, FALSE, FALSE, FALSE,

FALSE, FALSE, FALSE, FALSE, FALSE, FALSE, FALSE, FALSE, FALSE,

FALSE, FALSE, FALSE, FALSE, FALSE, FALSE, TRUE, TRUE, FALSE,

FALSE, FALSE, FALSE, FALSE, FALSE, FALSE, FALSE, FALSE, FALSE,

FALSE, FALSE, FALSE, FALSE, FALSE, FALSE, FALSE, FALSE, FALSE,

FALSE, FALSE, FALSE, FALSE, FALSE, FALSE, FALSE, FALSE, FALSE,

FALSE, FALSE, FALSE, FALSE, FALSE, FALSE, FALSE, FALSE, FALSE,

FALSE, FALSE, FALSE, FALSE, FALSE, FALSE, TRUE, FALSE, FALSE,

FALSE, FALSE, FALSE, FALSE, FALSE, FALSE, FALSE, FALSE, FALSE,

FALSE, FALSE, FALSE, FALSE, FALSE, FALSE, FALSE, FALSE, FALSE,

FALSE, FALSE, FALSE, FALSE, FALSE, FALSE, FALSE, FALSE, FALSE,

FALSE, FALSE, FALSE, FALSE, FALSE, FALSE, FALSE, FALSE, FALSE,

FALSE, FALSE, FALSE), OUTCOMES_L2_SYMPTOM_fever = c(TRUE, TRUE,

TRUE, FALSE, FALSE, TRUE, FALSE, FALSE, FALSE, FALSE, FALSE,

FALSE, FALSE, FALSE, FALSE, TRUE, TRUE, FALSE, FALSE, TRUE, FALSE,

TRUE, TRUE, FALSE, TRUE, FALSE, FALSE, TRUE, FALSE, FALSE, FALSE,

TRUE, TRUE, TRUE, TRUE, TRUE, FALSE, FALSE, FALSE, FALSE, FALSE,

FALSE, TRUE, FALSE, FALSE, TRUE, FALSE, FALSE, TRUE, TRUE, FALSE,

FALSE, TRUE, TRUE, FALSE, FALSE, TRUE, FALSE, FALSE, FALSE, TRUE,

FALSE, FALSE, TRUE, TRUE, TRUE, FALSE, FALSE, FALSE, TRUE, FALSE,

FALSE, FALSE, TRUE, FALSE, FALSE, FALSE, FALSE, FALSE, FALSE,

FALSE, FALSE, FALSE, FALSE, FALSE, TRUE, FALSE, TRUE, FALSE,

FALSE, FALSE, FALSE, FALSE, FALSE, FALSE, TRUE, FALSE, TRUE,

FALSE, FALSE, TRUE, FALSE, FALSE, FALSE, FALSE, FALSE, TRUE,

FALSE, FALSE, TRUE, TRUE, FALSE, FALSE, FALSE, FALSE, FALSE,

FALSE, FALSE, TRUE, FALSE, FALSE, FALSE, TRUE, FALSE, FALSE,

FALSE, FALSE, FALSE, FALSE, TRUE, TRUE, FALSE, FALSE, FALSE,

FALSE, FALSE, FALSE, FALSE, FALSE), OUTCOMES_L2_SYMPTOM_haemoptysis = c(FALSE,

FALSE, FALSE, FALSE, FALSE, FALSE, FALSE, FALSE, FALSE, FALSE,

FALSE, FALSE, FALSE, FALSE, FALSE, FALSE, FALSE, FALSE, FALSE,

FALSE, FALSE, FALSE, FALSE, TRUE, FALSE, FALSE, FALSE, FALSE,

FALSE, FALSE, FALSE, TRUE, FALSE, FALSE, FALSE, FALSE, FALSE,

FALSE, FALSE, FALSE, FALSE, FALSE, FALSE, FALSE, FALSE, FALSE,

FALSE, FALSE, TRUE, TRUE, FALSE, FALSE, TRUE, TRUE, FALSE, FALSE,

FALSE, FALSE, FALSE, FALSE, FALSE, FALSE, FALSE, FALSE, FALSE,

FALSE, FALSE, FALSE, FALSE, FALSE, FALSE, FALSE, FALSE, FALSE,

FALSE, FALSE, FALSE, FALSE, FALSE, FALSE, FALSE, FALSE, FALSE,

FALSE, FALSE, FALSE, FALSE, FALSE, FALSE, FALSE, FALSE, FALSE,

FALSE, FALSE, FALSE, FALSE, FALSE, FALSE, FALSE, FALSE, FALSE,

FALSE, FALSE, FALSE, FALSE, FALSE, FALSE, FALSE, FALSE, FALSE,

FALSE, FALSE, FALSE, FALSE, FALSE, FALSE, FALSE, FALSE, FALSE,

FALSE, FALSE, FALSE, FALSE, FALSE, FALSE, FALSE, FALSE, FALSE,

FALSE, FALSE, FALSE, FALSE, FALSE, FALSE, TRUE, FALSE, FALSE,

FALSE, FALSE), OUTCOMES_L2_SYMPTOM_irritability = c(FALSE, FALSE,

FALSE, FALSE, FALSE, FALSE, FALSE, FALSE, FALSE, FALSE, FALSE,

FALSE, FALSE, FALSE, FALSE, FALSE, FALSE, FALSE, FALSE, FALSE,

FALSE, FALSE, FALSE, FALSE, FALSE, FALSE, FALSE, FALSE, FALSE,

FALSE, FALSE, FALSE, FALSE, FALSE, FALSE, FALSE, FALSE, FALSE,

FALSE, FALSE, FALSE, FALSE, FALSE, FALSE, FALSE, FALSE, FALSE,

FALSE, FALSE, FALSE, FALSE, FALSE, FALSE, FALSE, FALSE, FALSE,

FALSE, FALSE, FALSE, FALSE, FALSE, FALSE, FALSE, FALSE, FALSE,

FALSE, FALSE, FALSE, FALSE, FALSE, FALSE, FALSE, FALSE, FALSE,

FALSE, FALSE, FALSE, FALSE, FALSE, FALSE, FALSE, FALSE, FALSE,

FALSE, FALSE, FALSE, FALSE, TRUE, FALSE, FALSE, FALSE, FALSE,

FALSE, FALSE, FALSE, FALSE, FALSE, FALSE, FALSE, FALSE, FALSE,

FALSE, FALSE, FALSE, FALSE, FALSE, FALSE, FALSE, FALSE, FALSE,

FALSE, FALSE, FALSE, FALSE, FALSE, FALSE, FALSE, FALSE, FALSE,

FALSE, FALSE, FALSE, FALSE, FALSE, FALSE, FALSE, FALSE, FALSE,

FALSE, FALSE, FALSE, FALSE, FALSE, FALSE, FALSE, FALSE, FALSE,

FALSE, FALSE), OUTCOMES_L2_SYMPTOM_length_of_illness = c(TRUE,

FALSE, FALSE, FALSE, FALSE, FALSE, FALSE, FALSE, FALSE, FALSE,

FALSE, FALSE, FALSE, FALSE, FALSE, FALSE, FALSE, FALSE, FALSE,

FALSE, FALSE, FALSE, FALSE, FALSE, FALSE, FALSE, FALSE, FALSE,

FALSE, FALSE, FALSE, FALSE, FALSE, FALSE, FALSE, FALSE, FALSE,

TRUE, TRUE, FALSE, FALSE, FALSE, FALSE, FALSE, FALSE, FALSE,

FALSE, FALSE, FALSE, FALSE, FALSE, FALSE, FALSE, FALSE, FALSE,

FALSE, FALSE, FALSE, FALSE, FALSE, FALSE, FALSE, FALSE, FALSE,

FALSE, FALSE, FALSE, FALSE, FALSE, TRUE, FALSE, FALSE, FALSE,

FALSE, FALSE, FALSE, FALSE, FALSE, FALSE, FALSE, FALSE, FALSE,

FALSE, FALSE, FALSE, FALSE, FALSE, FALSE, FALSE, FALSE, FALSE,

FALSE, FALSE, TRUE, FALSE, FALSE, FALSE, FALSE, FALSE, FALSE,

FALSE, FALSE, FALSE, TRUE, FALSE, FALSE, FALSE, FALSE, FALSE,

FALSE, FALSE, FALSE, FALSE, FALSE, FALSE, FALSE, FALSE, FALSE,

TRUE, FALSE, FALSE, FALSE, FALSE, FALSE, FALSE, FALSE, FALSE,

TRUE, FALSE, FALSE, FALSE, FALSE, FALSE, TRUE, FALSE, FALSE,

FALSE, FALSE, FALSE), OUTCOMES_L2_SYMPTOM_loss_of_taste_or_smell = c(FALSE,

FALSE, TRUE, TRUE, FALSE, FALSE, FALSE, FALSE, FALSE, FALSE,

FALSE, FALSE, FALSE, FALSE, FALSE, FALSE, FALSE, FALSE, FALSE,

TRUE, FALSE, FALSE, FALSE, FALSE, FALSE, FALSE, FALSE, FALSE,

FALSE, FALSE, FALSE, FALSE, FALSE, FALSE, FALSE, TRUE, FALSE,

FALSE, FALSE, FALSE, FALSE, FALSE, FALSE, FALSE, FALSE, FALSE,

FALSE, FALSE, FALSE, FALSE, FALSE, FALSE, FALSE, FALSE, FALSE,

FALSE, TRUE, FALSE, FALSE, FALSE, FALSE, FALSE, FALSE, FALSE,

FALSE, FALSE, FALSE, FALSE, FALSE, FALSE, FALSE, FALSE, FALSE,

FALSE, FALSE, FALSE, FALSE, FALSE, FALSE, FALSE, FALSE, FALSE,

FALSE, FALSE, FALSE, FALSE, FALSE, FALSE, FALSE, FALSE, FALSE,

FALSE, FALSE, FALSE, FALSE, TRUE, FALSE, TRUE, FALSE, FALSE,

FALSE, FALSE, FALSE, FALSE, FALSE, FALSE, FALSE, FALSE, FALSE,

TRUE, FALSE, FALSE, FALSE, FALSE, FALSE, FALSE, FALSE, FALSE,

FALSE, FALSE, FALSE, FALSE, FALSE, FALSE, FALSE, FALSE, FALSE,

FALSE, FALSE, FALSE, FALSE, FALSE, FALSE, FALSE, FALSE, FALSE,

FALSE, FALSE, FALSE), OUTCOMES_L2_SYMPTOM_malaise_anorexia = c(FALSE,

FALSE, TRUE, FALSE, FALSE, TRUE, FALSE, FALSE, FALSE, FALSE,

FALSE, FALSE, FALSE, FALSE, FALSE, FALSE, FALSE, FALSE, FALSE,

TRUE, FALSE, TRUE, TRUE, FALSE, FALSE, FALSE, FALSE, TRUE, FALSE,

FALSE, FALSE, FALSE, FALSE, FALSE, FALSE, TRUE, FALSE, FALSE,

FALSE, FALSE, FALSE, FALSE, TRUE, FALSE, FALSE, FALSE, FALSE,

FALSE, FALSE, FALSE, FALSE, FALSE, TRUE, TRUE, FALSE, FALSE,

TRUE, FALSE, FALSE, FALSE, FALSE, FALSE, FALSE, TRUE, FALSE,

FALSE, FALSE, FALSE, FALSE, FALSE, FALSE, FALSE, FALSE, FALSE,

FALSE, FALSE, FALSE, FALSE, FALSE, FALSE, FALSE, FALSE, FALSE,

FALSE, FALSE, TRUE, FALSE, TRUE, FALSE, FALSE, FALSE, FALSE,

FALSE, FALSE, FALSE, TRUE, FALSE, TRUE, FALSE, FALSE, TRUE, FALSE,

FALSE, FALSE, FALSE, FALSE, TRUE, FALSE, FALSE, TRUE, FALSE,

FALSE, FALSE, FALSE, FALSE, TRUE, FALSE, FALSE, FALSE, FALSE,

FALSE, FALSE, FALSE, FALSE, FALSE, FALSE, FALSE, TRUE, TRUE,

FALSE, FALSE, FALSE, FALSE, TRUE, FALSE, FALSE, FALSE, FALSE,

FALSE), OUTCOMES_L2_SYMPTOM_myalgia_arthralgia = c(TRUE, TRUE,

FALSE, TRUE, FALSE, TRUE, FALSE, FALSE, FALSE, FALSE, FALSE,

FALSE, FALSE, FALSE, FALSE, TRUE, TRUE, FALSE, FALSE, TRUE, FALSE,

TRUE, FALSE, TRUE, FALSE, FALSE, FALSE, FALSE, FALSE, FALSE,

FALSE, FALSE, TRUE, TRUE, FALSE, FALSE, FALSE, FALSE, FALSE,

TRUE, TRUE, FALSE, TRUE, FALSE, FALSE, FALSE, FALSE, FALSE, FALSE,

FALSE, FALSE, FALSE, TRUE, TRUE, FALSE, FALSE, TRUE, FALSE, FALSE,

FALSE, FALSE, FALSE, FALSE, TRUE, FALSE, FALSE, FALSE, FALSE,

FALSE, TRUE, FALSE, FALSE, FALSE, TRUE, FALSE, FALSE, FALSE,

FALSE, FALSE, TRUE, FALSE, FALSE, FALSE, FALSE, FALSE, TRUE,

FALSE, TRUE, FALSE, FALSE, FALSE, TRUE, TRUE, FALSE, FALSE, TRUE,

FALSE, TRUE, FALSE, FALSE, TRUE, FALSE, FALSE, FALSE, FALSE,

FALSE, FALSE, FALSE, FALSE, TRUE, FALSE, FALSE, FALSE, FALSE,

FALSE, FALSE, FALSE, FALSE, FALSE, FALSE, FALSE, FALSE, FALSE,

FALSE, FALSE, FALSE, FALSE, FALSE, FALSE, FALSE, FALSE, FALSE,

FALSE, TRUE, FALSE, FALSE, FALSE, FALSE, FALSE), OUTCOMES_L2_SYMPTOM_nausea_vomiting = c(FALSE,

FALSE, FALSE, FALSE, FALSE, TRUE, FALSE, FALSE, FALSE, FALSE,

FALSE, FALSE, FALSE, FALSE, FALSE, FALSE, TRUE, FALSE, FALSE,

TRUE, TRUE, FALSE, TRUE, TRUE, FALSE, FALSE, FALSE, TRUE, FALSE,

FALSE, FALSE, TRUE, TRUE, TRUE, FALSE, TRUE, FALSE, FALSE, FALSE,

TRUE, TRUE, FALSE, FALSE, FALSE, FALSE, FALSE, FALSE, FALSE,

FALSE, FALSE, FALSE, FALSE, TRUE, TRUE, FALSE, FALSE, TRUE, FALSE,

FALSE, FALSE, FALSE, FALSE, FALSE, TRUE, FALSE, FALSE, FALSE,

FALSE, FALSE, FALSE, FALSE, FALSE, FALSE, FALSE, FALSE, FALSE,

FALSE, FALSE, FALSE, FALSE, FALSE, FALSE, FALSE, FALSE, FALSE,

FALSE, FALSE, FALSE, FALSE, FALSE, FALSE, TRUE, TRUE, FALSE,

FALSE, TRUE, FALSE, TRUE, FALSE, FALSE, FALSE, TRUE, FALSE, FALSE,

FALSE, FALSE, TRUE, FALSE, FALSE, FALSE, FALSE, FALSE, FALSE,

FALSE, FALSE, TRUE, FALSE, FALSE, FALSE, FALSE, FALSE, FALSE,

FALSE, FALSE, FALSE, FALSE, FALSE, FALSE, FALSE, TRUE, TRUE,

FALSE, FALSE, TRUE, FALSE, FALSE, FALSE, FALSE, FALSE), OUTCOMES_L2_SYMPTOM_retroocular_pain = c(FALSE,

FALSE, FALSE, FALSE, FALSE, FALSE, FALSE, FALSE, FALSE, FALSE,

FALSE, FALSE, FALSE, FALSE, FALSE, FALSE, FALSE, FALSE, FALSE,

FALSE, FALSE, FALSE, FALSE, FALSE, FALSE, FALSE, FALSE, FALSE,

FALSE, FALSE, FALSE, FALSE, FALSE, FALSE, FALSE, FALSE, FALSE,

FALSE, FALSE, FALSE, FALSE, FALSE, FALSE, FALSE, FALSE, FALSE,

FALSE, FALSE, FALSE, FALSE, FALSE, FALSE, FALSE, FALSE, FALSE,

FALSE, FALSE, FALSE, FALSE, FALSE, FALSE, FALSE, FALSE, FALSE,

FALSE, FALSE, FALSE, FALSE, FALSE, FALSE, FALSE, FALSE, FALSE,

FALSE, FALSE, FALSE, FALSE, FALSE, FALSE, FALSE, FALSE, FALSE,

FALSE, FALSE, FALSE, FALSE, FALSE, FALSE, FALSE, FALSE, FALSE,

FALSE, FALSE, FALSE, FALSE, FALSE, FALSE, TRUE, FALSE, FALSE,

FALSE, FALSE, FALSE, FALSE, FALSE, FALSE, FALSE, FALSE, FALSE,

FALSE, FALSE, FALSE, FALSE, FALSE, FALSE, FALSE, FALSE, FALSE,

FALSE, FALSE, FALSE, FALSE, FALSE, FALSE, FALSE, FALSE, FALSE,

FALSE, FALSE, FALSE, FALSE, FALSE, FALSE, FALSE, FALSE, FALSE,

FALSE, FALSE, FALSE), OUTCOMES_L2_SYMPTOM_shortness_of_breath = c(TRUE,

TRUE, TRUE, FALSE, FALSE, TRUE, FALSE, FALSE, FALSE, FALSE, FALSE,

FALSE, FALSE, FALSE, FALSE, TRUE, TRUE, FALSE, FALSE, TRUE, TRUE,

TRUE, FALSE, TRUE, FALSE, FALSE, FALSE, TRUE, FALSE, FALSE, FALSE,

FALSE, FALSE, FALSE, TRUE, TRUE, FALSE, FALSE, FALSE, TRUE, TRUE,

FALSE, TRUE, FALSE, FALSE, FALSE, FALSE, FALSE, TRUE, TRUE, FALSE,

FALSE, TRUE, TRUE, FALSE, FALSE, TRUE, FALSE, FALSE, FALSE, FALSE,

FALSE, FALSE, TRUE, FALSE, FALSE, FALSE, FALSE, FALSE, FALSE,

FALSE, FALSE, FALSE, FALSE, FALSE, FALSE, FALSE, TRUE, FALSE,

TRUE, FALSE, FALSE, FALSE, FALSE, FALSE, TRUE, FALSE, TRUE, TRUE,

FALSE, FALSE, TRUE, TRUE, FALSE, FALSE, TRUE, FALSE, TRUE, FALSE,

FALSE, FALSE, FALSE, FALSE, FALSE, FALSE, FALSE, FALSE, TRUE,

FALSE, TRUE, TRUE, FALSE, FALSE, FALSE, FALSE, TRUE, FALSE, FALSE,

TRUE, FALSE, FALSE, FALSE, TRUE, FALSE, FALSE, FALSE, FALSE,

TRUE, TRUE, TRUE, TRUE, FALSE, FALSE, FALSE, TRUE, FALSE, FALSE,

FALSE, FALSE), OUTCOMES_L2_SYMPTOM_sorethroat = c(TRUE, TRUE,

TRUE, FALSE, FALSE, TRUE, FALSE, FALSE, FALSE, FALSE, FALSE,

FALSE, FALSE, FALSE, FALSE, FALSE, FALSE, FALSE, FALSE, FALSE,

TRUE, FALSE, TRUE, TRUE, TRUE, FALSE, FALSE, FALSE, FALSE, FALSE,

FALSE, TRUE, TRUE, TRUE, FALSE, FALSE, FALSE, FALSE, FALSE, TRUE,

TRUE, FALSE, TRUE, FALSE, FALSE, TRUE, FALSE, FALSE, TRUE, TRUE,

FALSE, FALSE, TRUE, TRUE, FALSE, FALSE, TRUE, FALSE, FALSE, FALSE,

FALSE, FALSE, FALSE, TRUE, FALSE, TRUE, FALSE, FALSE, FALSE,

TRUE, FALSE, FALSE, FALSE, TRUE, FALSE, FALSE, FALSE, FALSE,

FALSE, TRUE, FALSE, FALSE, FALSE, FALSE, FALSE, FALSE, FALSE,

TRUE, FALSE, FALSE, FALSE, TRUE, TRUE, FALSE, FALSE, TRUE, FALSE,

TRUE, FALSE, FALSE, TRUE, FALSE, FALSE, FALSE, FALSE, FALSE,

FALSE, TRUE, FALSE, TRUE, FALSE, FALSE, FALSE, FALSE, FALSE,

FALSE, FALSE, FALSE, FALSE, FALSE, FALSE, FALSE, FALSE, FALSE,

FALSE, FALSE, FALSE, FALSE, FALSE, FALSE, TRUE, FALSE, FALSE,

TRUE, TRUE, FALSE, FALSE, FALSE, FALSE), OUTCOMES_L2_ABSENCE_school_absence = c(FALSE,

FALSE, FALSE, FALSE, FALSE, FALSE, FALSE, FALSE, FALSE, FALSE,

FALSE, FALSE, FALSE, FALSE, FALSE, FALSE, FALSE, FALSE, FALSE,

FALSE, FALSE, FALSE, FALSE, FALSE, FALSE, FALSE, FALSE, FALSE,

FALSE, FALSE, FALSE, FALSE, FALSE, FALSE, FALSE, FALSE, FALSE,

TRUE, TRUE, FALSE, FALSE, FALSE, FALSE, FALSE, FALSE, FALSE,

FALSE, FALSE, FALSE, FALSE, FALSE, FALSE, FALSE, FALSE, FALSE,

FALSE, FALSE, FALSE, FALSE, TRUE, FALSE, FALSE, FALSE, FALSE,

TRUE, FALSE, FALSE, FALSE, FALSE, TRUE, FALSE, FALSE, FALSE,

FALSE, FALSE, FALSE, FALSE, FALSE, FALSE, FALSE, FALSE, FALSE,

FALSE, FALSE, FALSE, FALSE, FALSE, FALSE, FALSE, FALSE, FALSE,

FALSE, FALSE, FALSE, FALSE, FALSE, FALSE, FALSE, FALSE, FALSE,

FALSE, FALSE, FALSE, FALSE, FALSE, FALSE, FALSE, FALSE, FALSE,

FALSE, FALSE, FALSE, FALSE, FALSE, FALSE, FALSE, FALSE, FALSE,

FALSE, FALSE, FALSE, FALSE, FALSE, FALSE, FALSE, FALSE, FALSE,

FALSE, FALSE, FALSE, FALSE, FALSE, FALSE, FALSE, FALSE, FALSE,

FALSE, FALSE, FALSE), OUTCOMES_L2_ABSENCE_work_absence = c(FALSE,

FALSE, FALSE, FALSE, FALSE, FALSE, FALSE, FALSE, FALSE, TRUE,

FALSE, FALSE, FALSE, FALSE, FALSE, FALSE, FALSE, FALSE, FALSE,

FALSE, FALSE, FALSE, FALSE, FALSE, FALSE, FALSE, FALSE, FALSE,

FALSE, FALSE, FALSE, FALSE, FALSE, FALSE, FALSE, FALSE, FALSE,

TRUE, TRUE, FALSE, FALSE, FALSE, FALSE, FALSE, FALSE, FALSE,

FALSE, FALSE, FALSE, FALSE, FALSE, FALSE, FALSE, FALSE, FALSE,

FALSE, FALSE, FALSE, FALSE, TRUE, FALSE, FALSE, FALSE, FALSE,

TRUE, FALSE, FALSE, FALSE, FALSE, TRUE, FALSE, FALSE, FALSE,

FALSE, FALSE, FALSE, FALSE, FALSE, FALSE, FALSE, FALSE, FALSE,

FALSE, FALSE, FALSE, FALSE, FALSE, FALSE, FALSE, FALSE, FALSE,

FALSE, FALSE, FALSE, FALSE, FALSE, FALSE, FALSE, FALSE, FALSE,

FALSE, FALSE, FALSE, FALSE, FALSE, FALSE, FALSE, FALSE, FALSE,

FALSE, FALSE, FALSE, FALSE, FALSE, FALSE, FALSE, FALSE, FALSE,

FALSE, FALSE, FALSE, FALSE, FALSE, FALSE, FALSE, FALSE, FALSE,

FALSE, FALSE, FALSE, FALSE, FALSE, FALSE, TRUE, FALSE, FALSE,

FALSE, FALSE, FALSE), OUTCOMES_L2_SIGNS_blood_pressure = c(FALSE,

FALSE, FALSE, TRUE, FALSE, FALSE, FALSE, FALSE, FALSE, FALSE,

FALSE, FALSE, FALSE, FALSE, FALSE, FALSE, FALSE, FALSE, FALSE,

FALSE, FALSE, FALSE, FALSE, FALSE, FALSE, FALSE, FALSE, FALSE,

FALSE, FALSE, FALSE, FALSE, FALSE, FALSE, FALSE, FALSE, FALSE,

FALSE, FALSE, FALSE, FALSE, FALSE, FALSE, FALSE, FALSE, FALSE,

FALSE, FALSE, TRUE, TRUE, FALSE, FALSE, FALSE, FALSE, FALSE,

FALSE, FALSE, FALSE, FALSE, FALSE, FALSE, FALSE, FALSE, FALSE,

FALSE, FALSE, FALSE, FALSE, FALSE, FALSE, FALSE, FALSE, FALSE,

FALSE, FALSE, FALSE, FALSE, FALSE, FALSE, FALSE, FALSE, FALSE,

FALSE, FALSE, FALSE, FALSE, FALSE, FALSE, TRUE, FALSE, FALSE,

FALSE, FALSE, FALSE, FALSE, FALSE, FALSE, FALSE, FALSE, FALSE,

FALSE, FALSE, FALSE, FALSE, FALSE, FALSE, FALSE, FALSE, FALSE,

FALSE, FALSE, FALSE, FALSE, FALSE, FALSE, FALSE, FALSE, FALSE,

FALSE, FALSE, FALSE, FALSE, FALSE, FALSE, FALSE, FALSE, FALSE,

FALSE, FALSE, FALSE, FALSE, FALSE, FALSE, FALSE, FALSE, FALSE,

FALSE, FALSE, FALSE), OUTCOMES_L2_SIGNS_chest_signs = c(TRUE,

TRUE, FALSE, FALSE, FALSE, FALSE, FALSE, FALSE, FALSE, FALSE,

FALSE, FALSE, FALSE, FALSE, FALSE, FALSE, FALSE, FALSE, FALSE,

FALSE, TRUE, FALSE, FALSE, TRUE, FALSE, FALSE, FALSE, TRUE, FALSE,

FALSE, FALSE, FALSE, FALSE, FALSE, FALSE, FALSE, FALSE, FALSE,

FALSE, FALSE, FALSE, FALSE, FALSE, FALSE, FALSE, TRUE, FALSE,

FALSE, FALSE, FALSE, FALSE, FALSE, FALSE, FALSE, FALSE, FALSE,

FALSE, FALSE, FALSE, FALSE, FALSE, FALSE, FALSE, FALSE, FALSE,

FALSE, FALSE, FALSE, FALSE, FALSE, FALSE, FALSE, TRUE, FALSE,

FALSE, FALSE, FALSE, TRUE, FALSE, FALSE, FALSE, FALSE, FALSE,

FALSE, FALSE, FALSE, FALSE, FALSE, TRUE, FALSE, FALSE, TRUE,

TRUE, FALSE, FALSE, FALSE, FALSE, FALSE, FALSE, FALSE, FALSE,

FALSE, FALSE, FALSE, FALSE, FALSE, FALSE, FALSE, FALSE, FALSE,

FALSE, FALSE, FALSE, FALSE, FALSE, FALSE, FALSE, FALSE, FALSE,

FALSE, FALSE, FALSE, TRUE, FALSE, FALSE, FALSE, FALSE, FALSE,

FALSE, FALSE, FALSE, FALSE, FALSE, FALSE, TRUE, FALSE, FALSE,

FALSE, FALSE), OUTCOMES_L2_SIGNS_cyanosis = c(FALSE, FALSE, FALSE,

FALSE, FALSE, FALSE, FALSE, FALSE, FALSE, FALSE, FALSE, FALSE,

FALSE, FALSE, FALSE, FALSE, FALSE, FALSE, FALSE, FALSE, FALSE,

FALSE, FALSE, FALSE, FALSE, FALSE, FALSE, FALSE, FALSE, FALSE,

FALSE, FALSE, FALSE, FALSE, FALSE, FALSE, FALSE, FALSE, FALSE,

FALSE, FALSE, FALSE, TRUE, FALSE, FALSE, FALSE, FALSE, FALSE,

FALSE, FALSE, FALSE, FALSE, FALSE, FALSE, FALSE, FALSE, FALSE,

FALSE, FALSE, FALSE, FALSE, FALSE, FALSE, FALSE, FALSE, FALSE,

FALSE, FALSE, FALSE, FALSE, FALSE, FALSE, FALSE, FALSE, FALSE,

FALSE, FALSE, FALSE, FALSE, FALSE, FALSE, FALSE, FALSE, FALSE,

FALSE, FALSE, FALSE, TRUE, TRUE, FALSE, FALSE, FALSE, FALSE,

FALSE, FALSE, FALSE, FALSE, FALSE, FALSE, FALSE, FALSE, FALSE,

FALSE, FALSE, FALSE, FALSE, TRUE, FALSE, FALSE, FALSE, FALSE,

FALSE, FALSE, FALSE, FALSE, FALSE, FALSE, FALSE, FALSE, FALSE,

FALSE, FALSE, FALSE, FALSE, FALSE, FALSE, FALSE, FALSE, FALSE,

FALSE, FALSE, FALSE, FALSE, FALSE, FALSE, FALSE, FALSE, FALSE,

FALSE), OUTCOMES_L2_SIGNS_o2_saturation = c(FALSE, FALSE, FALSE,

TRUE, FALSE, FALSE, FALSE, FALSE, FALSE, FALSE, FALSE, FALSE,

FALSE, FALSE, FALSE, FALSE, TRUE, FALSE, FALSE, FALSE, FALSE,

FALSE, FALSE, FALSE, FALSE, FALSE, FALSE, FALSE, FALSE, FALSE,

FALSE, FALSE, FALSE, FALSE, FALSE, FALSE, FALSE, FALSE, FALSE,

FALSE, FALSE, FALSE, FALSE, FALSE, FALSE, FALSE, FALSE, FALSE,

FALSE, FALSE, FALSE, FALSE, TRUE, TRUE, FALSE, FALSE, FALSE,

FALSE, FALSE, FALSE, FALSE, FALSE, FALSE, FALSE, FALSE, TRUE,

FALSE, FALSE, FALSE, FALSE, FALSE, FALSE, FALSE, FALSE, FALSE,

FALSE, FALSE, TRUE, FALSE, FALSE, FALSE, FALSE, FALSE, FALSE,

FALSE, FALSE, FALSE, FALSE, TRUE, FALSE, FALSE, FALSE, FALSE,

FALSE, FALSE, FALSE, FALSE, FALSE, FALSE, FALSE, FALSE, TRUE,

FALSE, FALSE, FALSE, FALSE, FALSE, FALSE, FALSE, FALSE, FALSE,

FALSE, FALSE, FALSE, TRUE, TRUE, FALSE, TRUE, TRUE, FALSE, FALSE,

FALSE, TRUE, FALSE, FALSE, FALSE, FALSE, TRUE, TRUE, FALSE, FALSE,

FALSE, FALSE, FALSE, FALSE, FALSE, FALSE, FALSE, FALSE), OUTCOMES_L2_SIGNS_pulse_rate = c(FALSE,

FALSE, FALSE, TRUE, FALSE, FALSE, FALSE, FALSE, FALSE, FALSE,

FALSE, FALSE, FALSE, FALSE, FALSE, FALSE, FALSE, FALSE, FALSE,

FALSE, FALSE, FALSE, FALSE, FALSE, FALSE, FALSE, FALSE, FALSE,

FALSE, FALSE, FALSE, FALSE, FALSE, FALSE, FALSE, FALSE, FALSE,

FALSE, FALSE, FALSE, FALSE, FALSE, FALSE, FALSE, FALSE, FALSE,

FALSE, TRUE, FALSE, FALSE, FALSE, FALSE, TRUE, TRUE, FALSE, FALSE,

FALSE, FALSE, FALSE, FALSE, FALSE, FALSE, FALSE, FALSE, FALSE,

FALSE, FALSE, FALSE, FALSE, FALSE, FALSE, FALSE, TRUE, FALSE,

FALSE, FALSE, FALSE, FALSE, FALSE, FALSE, FALSE, FALSE, FALSE,

FALSE, FALSE, FALSE, FALSE, FALSE, TRUE, FALSE, FALSE, FALSE,

FALSE, FALSE, FALSE, FALSE, FALSE, FALSE, FALSE, FALSE, FALSE,

FALSE, FALSE, FALSE, FALSE, FALSE, FALSE, FALSE, FALSE, FALSE,

FALSE, FALSE, FALSE, FALSE, FALSE, FALSE, FALSE, FALSE, FALSE,

FALSE, FALSE, FALSE, FALSE, FALSE, FALSE, FALSE, FALSE, FALSE,

FALSE, FALSE, FALSE, FALSE, FALSE, FALSE, FALSE, FALSE, FALSE,

FALSE, FALSE), OUTCOMES_L2_SIGNS_respiratory_rate = c(FALSE,

FALSE, FALSE, TRUE, FALSE, FALSE, FALSE, FALSE, FALSE, FALSE,

FALSE, FALSE, FALSE, FALSE, FALSE, FALSE, FALSE, FALSE, FALSE,

FALSE, TRUE, FALSE, FALSE, TRUE, FALSE, FALSE, FALSE, TRUE, FALSE,

FALSE, FALSE, FALSE, FALSE, FALSE, FALSE, FALSE, FALSE, FALSE,

FALSE, FALSE, FALSE, FALSE, FALSE, FALSE, FALSE, FALSE, FALSE,

TRUE, FALSE, FALSE, FALSE, FALSE, FALSE, FALSE, FALSE, FALSE,

FALSE, FALSE, FALSE, FALSE, FALSE, FALSE, FALSE, FALSE, FALSE,

TRUE, FALSE, FALSE, FALSE, FALSE, FALSE, FALSE, FALSE, FALSE,

FALSE, FALSE, FALSE, TRUE, FALSE, FALSE, FALSE, FALSE, FALSE,

FALSE, FALSE, FALSE, FALSE, TRUE, TRUE, FALSE, FALSE, FALSE,

FALSE, FALSE, FALSE, FALSE, FALSE, FALSE, FALSE, FALSE, FALSE,

TRUE, FALSE, FALSE, FALSE, FALSE, FALSE, FALSE, FALSE, FALSE,

FALSE, FALSE, FALSE, FALSE, TRUE, FALSE, FALSE, FALSE, TRUE,

FALSE, FALSE, FALSE, TRUE, FALSE, FALSE, FALSE, FALSE, FALSE,

FALSE, FALSE, FALSE, FALSE, FALSE, FALSE, TRUE, FALSE, FALSE,

FALSE, FALSE), OUTCOMES_L2_SIGNS_respiratory_distress = c(FALSE,

FALSE, FALSE, FALSE, FALSE, FALSE, FALSE, FALSE, FALSE, FALSE,

FALSE, FALSE, FALSE, FALSE, FALSE, FALSE, FALSE, FALSE, FALSE,

FALSE, FALSE, FALSE, FALSE, FALSE, FALSE, FALSE, FALSE, FALSE,

FALSE, FALSE, FALSE, FALSE, FALSE, FALSE, FALSE, FALSE, FALSE,

FALSE, FALSE, FALSE, FALSE, FALSE, FALSE, FALSE, FALSE, FALSE,

FALSE, FALSE, FALSE, FALSE, FALSE, FALSE, TRUE, TRUE, FALSE,

FALSE, FALSE, FALSE, FALSE, FALSE, FALSE, FALSE, FALSE, FALSE,

FALSE, FALSE, FALSE, FALSE, FALSE, FALSE, FALSE, FALSE, FALSE,

FALSE, FALSE, FALSE, FALSE, FALSE, FALSE, FALSE, FALSE, FALSE,

FALSE, FALSE, FALSE, FALSE, FALSE, FALSE, FALSE, FALSE, FALSE,

FALSE, FALSE, FALSE, FALSE, FALSE, FALSE, FALSE, FALSE, FALSE,

FALSE, TRUE, FALSE, FALSE, FALSE, FALSE, TRUE, FALSE, FALSE,

FALSE, FALSE, FALSE, FALSE, FALSE, FALSE, TRUE, FALSE, FALSE,

FALSE, FALSE, FALSE, FALSE, FALSE, FALSE, FALSE, FALSE, FALSE,

TRUE, TRUE, FALSE, FALSE, FALSE, FALSE, FALSE, FALSE, FALSE,

FALSE, FALSE, FALSE), OUTCOMES_L2_SIGNS_temperature = c(FALSE,

FALSE, FALSE, FALSE, FALSE, FALSE, FALSE, FALSE, FALSE, FALSE,

FALSE, FALSE, FALSE, FALSE, FALSE, FALSE, FALSE, FALSE, FALSE,

FALSE, TRUE, FALSE, FALSE, FALSE, FALSE, FALSE, FALSE, FALSE,

FALSE, FALSE, FALSE, FALSE, FALSE, FALSE, FALSE, FALSE, FALSE,

FALSE, FALSE, TRUE, TRUE, FALSE, FALSE, FALSE, FALSE, FALSE,

FALSE, TRUE, FALSE, FALSE, FALSE, FALSE, FALSE, FALSE, FALSE,

FALSE, TRUE, FALSE, FALSE, FALSE, TRUE, FALSE, FALSE, FALSE,

TRUE, TRUE, FALSE, FALSE, FALSE, FALSE, FALSE, FALSE, TRUE, TRUE,

FALSE, FALSE, FALSE, FALSE, FALSE, TRUE, FALSE, FALSE, FALSE,

FALSE, FALSE, FALSE, FALSE, FALSE, FALSE, FALSE, FALSE, FALSE,

FALSE, FALSE, FALSE, FALSE, FALSE, FALSE, FALSE, FALSE, FALSE,

TRUE, FALSE, FALSE, FALSE, FALSE, FALSE, TRUE, FALSE, FALSE,

FALSE, FALSE, FALSE, FALSE, TRUE, TRUE, FALSE, FALSE, FALSE,

FALSE, FALSE, FALSE, TRUE, FALSE, FALSE, FALSE, FALSE, FALSE,

FALSE, FALSE, FALSE, FALSE, FALSE, FALSE, TRUE, FALSE, FALSE,

FALSE, FALSE), OUTCOMES_L2_SCORE_american_academy_pediatrics_guideline_criteria = c(FALSE,

FALSE, FALSE, FALSE, FALSE, FALSE, FALSE, FALSE, FALSE, FALSE,

FALSE, FALSE, FALSE, FALSE, FALSE, FALSE, FALSE, FALSE, FALSE,

FALSE, FALSE, FALSE, FALSE, FALSE, FALSE, FALSE, FALSE, FALSE,

FALSE, FALSE, FALSE, FALSE, FALSE, FALSE, FALSE, FALSE, FALSE,

FALSE, FALSE, FALSE, FALSE, FALSE, FALSE, FALSE, FALSE, FALSE,

FALSE, FALSE, FALSE, FALSE, FALSE, FALSE, FALSE, FALSE, FALSE,

FALSE, FALSE, FALSE, FALSE, FALSE, FALSE, FALSE, FALSE, FALSE,

TRUE, FALSE, FALSE, FALSE, FALSE, FALSE, FALSE, FALSE, FALSE,

FALSE, FALSE, FALSE, FALSE, FALSE, FALSE, FALSE, FALSE, FALSE,

FALSE, FALSE, FALSE, FALSE, FALSE, FALSE, FALSE, FALSE, FALSE,

FALSE, FALSE, FALSE, FALSE, FALSE, FALSE, FALSE, FALSE, FALSE,

FALSE, FALSE, FALSE, FALSE, FALSE, FALSE, FALSE, FALSE, FALSE,

FALSE, FALSE, FALSE, FALSE, FALSE, FALSE, FALSE, FALSE, FALSE,

FALSE, FALSE, FALSE, FALSE, FALSE, FALSE, FALSE, FALSE, FALSE,

FALSE, FALSE, FALSE, FALSE, FALSE, FALSE, FALSE, FALSE, FALSE,

FALSE, FALSE, FALSE), OUTCOMES_L2_SCORE_apache_iv = c(FALSE,

FALSE, FALSE, FALSE, FALSE, FALSE, FALSE, FALSE, FALSE, FALSE,

FALSE, FALSE, FALSE, FALSE, FALSE, FALSE, FALSE, FALSE, FALSE,

FALSE, FALSE, FALSE, FALSE, FALSE, FALSE, FALSE, FALSE, FALSE,

FALSE, FALSE, FALSE, FALSE, FALSE, FALSE, FALSE, FALSE, FALSE,

FALSE, FALSE, FALSE, FALSE, FALSE, FALSE, FALSE, FALSE, FALSE,

FALSE, FALSE, FALSE, FALSE, FALSE, FALSE, FALSE, FALSE, FALSE,

FALSE, FALSE, FALSE, FALSE, FALSE, FALSE, TRUE, FALSE, FALSE,

FALSE, FALSE, FALSE, FALSE, FALSE, FALSE, FALSE, FALSE, FALSE,

FALSE, FALSE, FALSE, FALSE, FALSE, FALSE, FALSE, FALSE, FALSE,

FALSE, FALSE, FALSE, FALSE, FALSE, FALSE, FALSE, FALSE, FALSE,

FALSE, FALSE, FALSE, FALSE, FALSE, FALSE, FALSE, FALSE, FALSE,

FALSE, FALSE, FALSE, FALSE, FALSE, FALSE, FALSE, FALSE, FALSE,

FALSE, FALSE, FALSE, FALSE, FALSE, FALSE, FALSE, FALSE, FALSE,

FALSE, FALSE, FALSE, FALSE, FALSE, FALSE, FALSE, FALSE, FALSE,

FALSE, FALSE, FALSE, FALSE, FALSE, FALSE, FALSE, FALSE, FALSE,

FALSE, FALSE, FALSE), OUTCOMES_L2_SCORE_barthel_index = c(FALSE,

FALSE, FALSE, FALSE, FALSE, FALSE, FALSE, FALSE, FALSE, FALSE,

FALSE, FALSE, FALSE, FALSE, FALSE, FALSE, FALSE, FALSE, FALSE,

FALSE, FALSE, FALSE, FALSE, FALSE, FALSE, FALSE, FALSE, FALSE,

FALSE, FALSE, FALSE, FALSE, FALSE, FALSE, FALSE, FALSE, FALSE,

FALSE, FALSE, FALSE, FALSE, FALSE, FALSE, FALSE, FALSE, FALSE,

FALSE, FALSE, FALSE, FALSE, FALSE, FALSE, FALSE, FALSE, FALSE,

FALSE, FALSE, FALSE, FALSE, FALSE, FALSE, FALSE, FALSE, FALSE,

FALSE, FALSE, FALSE, FALSE, FALSE, FALSE, FALSE, FALSE, FALSE,

FALSE, FALSE, FALSE, FALSE, FALSE, FALSE, FALSE, FALSE, FALSE,

FALSE, FALSE, FALSE, FALSE, FALSE, FALSE, FALSE, FALSE, FALSE,

FALSE, FALSE, FALSE, FALSE, FALSE, TRUE, FALSE, FALSE, FALSE,

FALSE, FALSE, FALSE, FALSE, FALSE, FALSE, FALSE, FALSE, FALSE,

FALSE, FALSE, FALSE, FALSE, FALSE, FALSE, FALSE, FALSE, FALSE,

FALSE, FALSE, FALSE, FALSE, FALSE, FALSE, FALSE, FALSE, FALSE,

FALSE, FALSE, FALSE, FALSE, FALSE, FALSE, FALSE, FALSE, FALSE,

FALSE, FALSE, FALSE), OUTCOMES_L2_SCORE_euroqol = c(FALSE, FALSE,

FALSE, FALSE, FALSE, FALSE, FALSE, FALSE, FALSE, FALSE, FALSE,

FALSE, FALSE, FALSE, FALSE, FALSE, FALSE, FALSE, FALSE, FALSE,

FALSE, FALSE, FALSE, FALSE, FALSE, FALSE, FALSE, FALSE, FALSE,

FALSE, FALSE, FALSE, FALSE, FALSE, FALSE, FALSE, FALSE, FALSE,

FALSE, FALSE, FALSE, FALSE, FALSE, FALSE, FALSE, FALSE, FALSE,

FALSE, FALSE, FALSE, FALSE, FALSE, FALSE, FALSE, FALSE, FALSE,

FALSE, FALSE, FALSE, FALSE, FALSE, FALSE, FALSE, FALSE, FALSE,

FALSE, FALSE, FALSE, FALSE, FALSE, FALSE, FALSE, FALSE, FALSE,

FALSE, FALSE, FALSE, FALSE, FALSE, FALSE, FALSE, FALSE, FALSE,

FALSE, FALSE, FALSE, FALSE, FALSE, FALSE, FALSE, FALSE, FALSE,

FALSE, FALSE, FALSE, FALSE, FALSE, TRUE, FALSE, FALSE, FALSE,

FALSE, FALSE, FALSE, FALSE, FALSE, FALSE, FALSE, FALSE, FALSE,

FALSE, FALSE, FALSE, FALSE, FALSE, FALSE, FALSE, FALSE, FALSE,

FALSE, FALSE, FALSE, FALSE, FALSE, FALSE, FALSE, FALSE, FALSE,

FALSE, FALSE, FALSE, FALSE, FALSE, FALSE, FALSE, FALSE, FALSE,

FALSE, FALSE), OUTCOMES_L2_SCORE_gcs = c(FALSE, FALSE, FALSE,

FALSE, FALSE, FALSE, FALSE, FALSE, FALSE, FALSE, FALSE, FALSE,

FALSE, FALSE, FALSE, FALSE, FALSE, FALSE, FALSE, FALSE, FALSE,

FALSE, FALSE, FALSE, FALSE, FALSE, FALSE, FALSE, FALSE, FALSE,

FALSE, FALSE, FALSE, FALSE, FALSE, FALSE, FALSE, FALSE, FALSE,

FALSE, FALSE, FALSE, FALSE, FALSE, FALSE, FALSE, FALSE, FALSE,

FALSE, FALSE, FALSE, FALSE, FALSE, FALSE, FALSE, FALSE, FALSE,

FALSE, FALSE, FALSE, FALSE, FALSE, FALSE, FALSE, FALSE, FALSE,

FALSE, FALSE, FALSE, FALSE, FALSE, FALSE, FALSE, FALSE, FALSE,

FALSE, FALSE, FALSE, FALSE, FALSE, FALSE, FALSE, FALSE, FALSE,

FALSE, FALSE, FALSE, FALSE, FALSE, FALSE, FALSE, FALSE, FALSE,

FALSE, FALSE, FALSE, FALSE, FALSE, FALSE, FALSE, FALSE, FALSE,

FALSE, FALSE, FALSE, FALSE, FALSE, FALSE, FALSE, FALSE, FALSE,

FALSE, FALSE, FALSE, FALSE, FALSE, FALSE, TRUE, FALSE, FALSE,

FALSE, FALSE, FALSE, FALSE, FALSE, FALSE, FALSE, FALSE, FALSE,

FALSE, FALSE, FALSE, FALSE, FALSE, FALSE, FALSE, FALSE, FALSE,

FALSE), OUTCOMES_L2_SCORE_iss = c(FALSE, FALSE, FALSE, FALSE,

FALSE, FALSE, FALSE, FALSE, FALSE, FALSE, FALSE, FALSE, FALSE,

FALSE, FALSE, FALSE, FALSE, FALSE, FALSE, FALSE, FALSE, FALSE,

FALSE, FALSE, FALSE, FALSE, FALSE, FALSE, FALSE, FALSE, FALSE,

FALSE, FALSE, FALSE, FALSE, FALSE, FALSE, FALSE, FALSE, FALSE,

FALSE, FALSE, FALSE, FALSE, FALSE, FALSE, FALSE, FALSE, FALSE,

FALSE, FALSE, FALSE, FALSE, FALSE, FALSE, FALSE, FALSE, FALSE,

FALSE, FALSE, FALSE, FALSE, FALSE, FALSE, FALSE, FALSE, FALSE,

FALSE, FALSE, FALSE, FALSE, FALSE, FALSE, FALSE, FALSE, FALSE,

FALSE, FALSE, FALSE, FALSE, FALSE, FALSE, FALSE, FALSE, FALSE,

FALSE, FALSE, FALSE, FALSE, FALSE, FALSE, FALSE, FALSE, FALSE,

FALSE, FALSE, FALSE, FALSE, FALSE, FALSE, TRUE, FALSE, FALSE,

FALSE, FALSE, FALSE, FALSE, FALSE, FALSE, FALSE, FALSE, FALSE,

FALSE, FALSE, FALSE, FALSE, FALSE, FALSE, FALSE, FALSE, FALSE,

FALSE, FALSE, FALSE, FALSE, FALSE, FALSE, FALSE, FALSE, FALSE,

FALSE, FALSE, FALSE, FALSE, FALSE, FALSE, FALSE, FALSE, FALSE

), OUTCOMES_L2_SCORE_mews = c(FALSE, FALSE, FALSE, FALSE, FALSE,

FALSE, FALSE, FALSE, FALSE, FALSE, FALSE, FALSE, FALSE, FALSE,

FALSE, FALSE, FALSE, FALSE, FALSE, FALSE, FALSE, FALSE, FALSE,

FALSE, FALSE, FALSE, FALSE, FALSE, FALSE, FALSE, FALSE, FALSE,

FALSE, FALSE, FALSE, FALSE, FALSE, FALSE, FALSE, FALSE, FALSE,

FALSE, FALSE, FALSE, FALSE, FALSE, FALSE, FALSE, FALSE, FALSE,

TRUE, FALSE, FALSE, FALSE, FALSE, FALSE, FALSE, FALSE, FALSE,

FALSE, FALSE, FALSE, FALSE, FALSE, FALSE, FALSE, FALSE, FALSE,

FALSE, FALSE, FALSE, FALSE, FALSE, FALSE, FALSE, FALSE, FALSE,

FALSE, FALSE, FALSE, FALSE, FALSE, FALSE, FALSE, FALSE, FALSE,

FALSE, FALSE, FALSE, FALSE, FALSE, FALSE, FALSE, FALSE, FALSE,

FALSE, FALSE, FALSE, FALSE, FALSE, FALSE, FALSE, FALSE, FALSE,

FALSE, FALSE, FALSE, FALSE, FALSE, FALSE, FALSE, FALSE, FALSE,

FALSE, FALSE, FALSE, FALSE, FALSE, FALSE, FALSE, FALSE, FALSE,

FALSE, FALSE, FALSE, FALSE, FALSE, FALSE, FALSE, FALSE, FALSE,

FALSE, FALSE, FALSE, FALSE, FALSE, FALSE, FALSE, FALSE), OUTCOMES_L2_SCORE_paediatric_chinese_medical_association = c(FALSE,

FALSE, FALSE, FALSE, FALSE, FALSE, FALSE, FALSE, FALSE, FALSE,

FALSE, FALSE, FALSE, FALSE, FALSE, FALSE, TRUE, FALSE, FALSE,

FALSE, FALSE, FALSE, FALSE, FALSE, FALSE, FALSE, FALSE, FALSE,

FALSE, FALSE, FALSE, FALSE, FALSE, FALSE, FALSE, FALSE, FALSE,

FALSE, FALSE, FALSE, FALSE, FALSE, FALSE, FALSE, FALSE, FALSE,

FALSE, FALSE, FALSE, FALSE, FALSE, FALSE, FALSE, FALSE, FALSE,

FALSE, FALSE, FALSE, FALSE, FALSE, FALSE, FALSE, FALSE, FALSE,

FALSE, FALSE, FALSE, FALSE, FALSE, FALSE, FALSE, FALSE, FALSE,

FALSE, FALSE, FALSE, FALSE, FALSE, FALSE, FALSE, FALSE, FALSE,

FALSE, FALSE, FALSE, FALSE, FALSE, FALSE, FALSE, FALSE, FALSE,

FALSE, FALSE, FALSE, FALSE, FALSE, FALSE, FALSE, FALSE, FALSE,

FALSE, FALSE, FALSE, FALSE, FALSE, FALSE, FALSE, FALSE, FALSE,

FALSE, FALSE, FALSE, FALSE, FALSE, FALSE, FALSE, FALSE, FALSE,

FALSE, FALSE, FALSE, FALSE, FALSE, FALSE, FALSE, FALSE, FALSE,

FALSE, FALSE, FALSE, FALSE, FALSE, FALSE, FALSE, FALSE, FALSE,

FALSE, FALSE, FALSE), OUTCOMES_L2_SCORE_pediatric_risk_of_mortality_III_score = c(FALSE,

FALSE, FALSE, FALSE, FALSE, FALSE, FALSE, FALSE, FALSE, FALSE,

FALSE, FALSE, FALSE, FALSE, FALSE, FALSE, FALSE, FALSE, FALSE,

FALSE, FALSE, FALSE, FALSE, FALSE, FALSE, FALSE, FALSE, FALSE,

FALSE, FALSE, FALSE, FALSE, FALSE, FALSE, FALSE, FALSE, FALSE,

FALSE, FALSE, FALSE, FALSE, FALSE, FALSE, FALSE, FALSE, FALSE,

FALSE, FALSE, FALSE, FALSE, FALSE, FALSE, FALSE, FALSE, FALSE,

FALSE, FALSE, FALSE, FALSE, FALSE, FALSE, FALSE, FALSE, FALSE,

FALSE, FALSE, FALSE, FALSE, FALSE, FALSE, FALSE, FALSE, FALSE,

FALSE, FALSE, FALSE, FALSE, FALSE, FALSE, FALSE, FALSE, FALSE,

FALSE, FALSE, FALSE, FALSE, TRUE, FALSE, FALSE, FALSE, FALSE,

FALSE, FALSE, FALSE, FALSE, FALSE, FALSE, FALSE, FALSE, FALSE,

FALSE, FALSE, FALSE, FALSE, FALSE, FALSE, FALSE, FALSE, FALSE,

FALSE, FALSE, FALSE, FALSE, FALSE, FALSE, FALSE, FALSE, FALSE,

FALSE, FALSE, FALSE, FALSE, FALSE, FALSE, FALSE, FALSE, FALSE,

FALSE, FALSE, FALSE, FALSE, FALSE, FALSE, FALSE, FALSE, FALSE,

FALSE, FALSE, FALSE), OUTCOMES_L2_SCORE_project_specific_composite = c(FALSE,

FALSE, TRUE, FALSE, TRUE, FALSE, FALSE, FALSE, FALSE, FALSE,

FALSE, FALSE, FALSE, TRUE, FALSE, FALSE, FALSE, FALSE, FALSE,

TRUE, FALSE, FALSE, FALSE, FALSE, FALSE, FALSE, FALSE, FALSE,

FALSE, FALSE, FALSE, FALSE, FALSE, FALSE, FALSE, FALSE, FALSE,

FALSE, FALSE, FALSE, FALSE, FALSE, FALSE, FALSE, FALSE, FALSE,

FALSE, FALSE, FALSE, FALSE, FALSE, FALSE, FALSE, FALSE, FALSE,

FALSE, FALSE, FALSE, FALSE, FALSE, FALSE, FALSE, FALSE, FALSE,

FALSE, TRUE, FALSE, FALSE, FALSE, FALSE, FALSE, FALSE, FALSE,

FALSE, FALSE, FALSE, FALSE, FALSE, FALSE, FALSE, FALSE, FALSE,

FALSE, FALSE, FALSE, FALSE, FALSE, FALSE, FALSE, TRUE, TRUE,

FALSE, FALSE, FALSE, FALSE, FALSE, FALSE, FALSE, FALSE, FALSE,

FALSE, TRUE, FALSE, FALSE, FALSE, FALSE, FALSE, FALSE, FALSE,

FALSE, FALSE, FALSE, FALSE, FALSE, FALSE, FALSE, FALSE, FALSE,

TRUE, FALSE, FALSE, FALSE, FALSE, FALSE, FALSE, FALSE, FALSE,

FALSE, FALSE, FALSE, FALSE, FALSE, FALSE, FALSE, FALSE, FALSE,

FALSE, FALSE, TRUE), OUTCOMES_L2_SCORE_saps_2_score = c(FALSE,

FALSE, FALSE, FALSE, FALSE, FALSE, FALSE, FALSE, FALSE, FALSE,

FALSE, FALSE, FALSE, FALSE, FALSE, FALSE, FALSE, FALSE, FALSE,

FALSE, FALSE, FALSE, FALSE, FALSE, FALSE, FALSE, FALSE, FALSE,

FALSE, FALSE, FALSE, FALSE, FALSE, FALSE, FALSE, FALSE, FALSE,

FALSE, FALSE, FALSE, FALSE, FALSE, FALSE, FALSE, FALSE, FALSE,

FALSE, FALSE, FALSE, FALSE, FALSE, FALSE, FALSE, FALSE, FALSE,

FALSE, FALSE, FALSE, FALSE, FALSE, FALSE, FALSE, FALSE, FALSE,

FALSE, FALSE, FALSE, FALSE, FALSE, FALSE, FALSE, FALSE, FALSE,

FALSE, FALSE, FALSE, FALSE, FALSE, FALSE, FALSE, FALSE, FALSE,

FALSE, FALSE, FALSE, FALSE, FALSE, FALSE, FALSE, FALSE, FALSE,

FALSE, FALSE, FALSE, FALSE, FALSE, FALSE, FALSE, FALSE, FALSE,

FALSE, FALSE, FALSE, FALSE, FALSE, FALSE, FALSE, FALSE, FALSE,

FALSE, FALSE, FALSE, FALSE, FALSE, FALSE, FALSE, FALSE, FALSE,

FALSE, FALSE, FALSE, FALSE, FALSE, FALSE, FALSE, FALSE, FALSE,

FALSE, FALSE, TRUE, FALSE, FALSE, FALSE, FALSE, FALSE, FALSE,

FALSE, FALSE, FALSE), OUTCOMES_L2_SCORE_sofa_score = c(FALSE,

FALSE, FALSE, TRUE, FALSE, FALSE, FALSE, FALSE, FALSE, FALSE,

FALSE, FALSE, FALSE, FALSE, FALSE, FALSE, FALSE, FALSE, FALSE,

FALSE, FALSE, FALSE, FALSE, FALSE, FALSE, FALSE, FALSE, FALSE,

FALSE, FALSE, FALSE, FALSE, FALSE, FALSE, FALSE, FALSE, FALSE,

FALSE, FALSE, FALSE, FALSE, FALSE, FALSE, FALSE, FALSE, FALSE,

FALSE, FALSE, FALSE, FALSE, FALSE, FALSE, FALSE, FALSE, FALSE,

FALSE, FALSE, FALSE, FALSE, FALSE, FALSE, FALSE, FALSE, FALSE,

FALSE, FALSE, FALSE, FALSE, FALSE, FALSE, FALSE, FALSE, FALSE,

FALSE, FALSE, FALSE, FALSE, FALSE, FALSE, FALSE, FALSE, FALSE,

FALSE, FALSE, FALSE, FALSE, FALSE, FALSE, FALSE, FALSE, FALSE,

FALSE, FALSE, FALSE, FALSE, FALSE, FALSE, FALSE, FALSE, FALSE,

FALSE, FALSE, FALSE, FALSE, FALSE, FALSE, FALSE, FALSE, FALSE,

FALSE, FALSE, FALSE, FALSE, FALSE, FALSE, FALSE, FALSE, FALSE,

FALSE, FALSE, FALSE, FALSE, FALSE, FALSE, FALSE, FALSE, FALSE,

FALSE, FALSE, TRUE, FALSE, FALSE, FALSE, FALSE, FALSE, FALSE,

TRUE, FALSE, FALSE), OUTCOMES_L2_SCORE_vivl_score = c(FALSE,

FALSE, FALSE, FALSE, FALSE, FALSE, FALSE, FALSE, FALSE, FALSE,

FALSE, FALSE, FALSE, FALSE, FALSE, FALSE, FALSE, FALSE, FALSE,

FALSE, FALSE, FALSE, FALSE, FALSE, FALSE, FALSE, FALSE, FALSE,

FALSE, FALSE, FALSE, FALSE, FALSE, FALSE, FALSE, FALSE, FALSE,

FALSE, FALSE, FALSE, FALSE, FALSE, FALSE, FALSE, FALSE, FALSE,

FALSE, FALSE, FALSE, FALSE, FALSE, FALSE, FALSE, FALSE, FALSE,

FALSE, FALSE, FALSE, FALSE, FALSE, FALSE, FALSE, FALSE, FALSE,

FALSE, FALSE, FALSE, FALSE, FALSE, FALSE, FALSE, FALSE, FALSE,

FALSE, FALSE, FALSE, TRUE, FALSE, FALSE, FALSE, FALSE, FALSE,

FALSE, FALSE, FALSE, FALSE, FALSE, FALSE, FALSE, FALSE, FALSE,

FALSE, FALSE, FALSE, FALSE, FALSE, FALSE, FALSE, FALSE, FALSE,

FALSE, FALSE, FALSE, FALSE, FALSE, FALSE, FALSE, FALSE, FALSE,

FALSE, FALSE, FALSE, FALSE, FALSE, FALSE, FALSE, FALSE, FALSE,

FALSE, FALSE, FALSE, FALSE, FALSE, FALSE, FALSE, FALSE, FALSE,

FALSE, FALSE, FALSE, FALSE, FALSE, FALSE, FALSE, FALSE, FALSE,

FALSE, FALSE, FALSE), OUTCOMES_L2_SCORE_who_severity = c(FALSE,

FALSE, FALSE, TRUE, FALSE, FALSE, FALSE, TRUE, FALSE, FALSE,

FALSE, FALSE, FALSE, FALSE, FALSE, FALSE, FALSE, FALSE, FALSE,

FALSE, FALSE, FALSE, FALSE, FALSE, FALSE, FALSE, FALSE, FALSE,

FALSE, FALSE, FALSE, FALSE, FALSE, FALSE, FALSE, FALSE, FALSE,

FALSE, FALSE, FALSE, FALSE, FALSE, FALSE, FALSE, FALSE, FALSE,

FALSE, FALSE, FALSE, FALSE, FALSE, FALSE, FALSE, FALSE, FALSE,

FALSE, FALSE, FALSE, FALSE, FALSE, FALSE, FALSE, FALSE, FALSE,

FALSE, FALSE, FALSE, FALSE, FALSE, FALSE, FALSE, FALSE, FALSE,

FALSE, FALSE, FALSE, FALSE, FALSE, FALSE, FALSE, FALSE, FALSE,

FALSE, FALSE, FALSE, FALSE, FALSE, FALSE, FALSE, FALSE, FALSE,

FALSE, FALSE, FALSE, FALSE, FALSE, FALSE, FALSE, FALSE, FALSE,

FALSE, FALSE, FALSE, FALSE, FALSE, FALSE, FALSE, FALSE, FALSE,

FALSE, FALSE, FALSE, FALSE, FALSE, FALSE, FALSE, FALSE, FALSE,

FALSE, FALSE, FALSE, FALSE, FALSE, FALSE, FALSE, FALSE, FALSE,

FALSE, FALSE, FALSE, FALSE, FALSE, FALSE, FALSE, FALSE, FALSE,

FALSE, FALSE, FALSE), OUTCOMES_L2_IX_arterial_blood_gas = c(FALSE,

FALSE, FALSE, FALSE, FALSE, FALSE, FALSE, FALSE, FALSE, FALSE,

FALSE, FALSE, FALSE, FALSE, FALSE, FALSE, FALSE, FALSE, FALSE,

FALSE, FALSE, FALSE, FALSE, FALSE, FALSE, FALSE, FALSE, FALSE,

FALSE, FALSE, FALSE, FALSE, FALSE, FALSE, FALSE, FALSE, FALSE,

FALSE, FALSE, FALSE, FALSE, FALSE, FALSE, FALSE, FALSE, FALSE,

FALSE, FALSE, FALSE, FALSE, FALSE, FALSE, FALSE, FALSE, FALSE,

FALSE, FALSE, FALSE, FALSE, FALSE, FALSE, FALSE, FALSE, FALSE,

FALSE, FALSE, FALSE, FALSE, FALSE, FALSE, FALSE, FALSE, FALSE,

FALSE, FALSE, FALSE, FALSE, FALSE, FALSE, FALSE, FALSE, FALSE,

FALSE, FALSE, FALSE, FALSE, FALSE, FALSE, TRUE, FALSE, FALSE,

FALSE, FALSE, FALSE, FALSE, FALSE, FALSE, FALSE, FALSE, FALSE,

FALSE, FALSE, FALSE, FALSE, FALSE, FALSE, FALSE, FALSE, FALSE,

FALSE, FALSE, FALSE, FALSE, FALSE, FALSE, FALSE, FALSE, FALSE,

FALSE, FALSE, FALSE, FALSE, FALSE, FALSE, FALSE, FALSE, FALSE,

FALSE, FALSE, FALSE, FALSE, FALSE, FALSE, FALSE, FALSE, FALSE,

FALSE, FALSE, FALSE), OUTCOMES_L2_IX_chest_xray = c(FALSE, FALSE,

FALSE, FALSE, FALSE, FALSE, FALSE, FALSE, FALSE, FALSE, FALSE,

FALSE, FALSE, FALSE, FALSE, FALSE, FALSE, FALSE, FALSE, FALSE,

FALSE, FALSE, FALSE, FALSE, FALSE, FALSE, FALSE, FALSE, FALSE,

FALSE, FALSE, FALSE, FALSE, FALSE, FALSE, FALSE, TRUE, FALSE,

FALSE, FALSE, FALSE, FALSE, FALSE, FALSE, FALSE, FALSE, FALSE,

FALSE, FALSE, FALSE, FALSE, FALSE, FALSE, FALSE, FALSE, FALSE,

FALSE, FALSE, FALSE, FALSE, TRUE, FALSE, FALSE, FALSE, FALSE,

FALSE, FALSE, FALSE, FALSE, FALSE, FALSE, FALSE, TRUE, FALSE,

FALSE, FALSE, FALSE, TRUE, FALSE, FALSE, FALSE, FALSE, FALSE,

FALSE, FALSE, FALSE, FALSE, FALSE, FALSE, FALSE, FALSE, TRUE,

TRUE, FALSE, FALSE, FALSE, FALSE, FALSE, FALSE, FALSE, FALSE,

FALSE, FALSE, FALSE, FALSE, FALSE, TRUE, FALSE, FALSE, FALSE,

FALSE, FALSE, FALSE, FALSE, FALSE, FALSE, FALSE, FALSE, FALSE,

FALSE, FALSE, FALSE, TRUE, FALSE, FALSE, FALSE, FALSE, FALSE,

FALSE, FALSE, FALSE, FALSE, FALSE, FALSE, TRUE, FALSE, FALSE,

FALSE, FALSE), OUTCOMES_L2_IX_fibronogen = c(FALSE, FALSE, FALSE,

FALSE, FALSE, FALSE, FALSE, FALSE, FALSE, FALSE, FALSE, FALSE,

FALSE, FALSE, FALSE, FALSE, TRUE, FALSE, FALSE, FALSE, FALSE,

FALSE, FALSE, FALSE, FALSE, FALSE, FALSE, FALSE, FALSE, FALSE,

FALSE, FALSE, FALSE, FALSE, FALSE, FALSE, FALSE, FALSE, FALSE,

FALSE, FALSE, FALSE, FALSE, FALSE, FALSE, FALSE, FALSE, FALSE,

FALSE, FALSE, FALSE, FALSE, FALSE, FALSE, FALSE, FALSE, FALSE,

FALSE, FALSE, FALSE, FALSE, FALSE, FALSE, FALSE, FALSE, FALSE,

FALSE, FALSE, FALSE, FALSE, FALSE, FALSE, FALSE, FALSE, FALSE,

FALSE, FALSE, FALSE, FALSE, FALSE, FALSE, FALSE, FALSE, FALSE,

FALSE, FALSE, FALSE, FALSE, FALSE, FALSE, FALSE, FALSE, FALSE,

FALSE, FALSE, FALSE, FALSE, FALSE, FALSE, FALSE, FALSE, FALSE,

FALSE, FALSE, FALSE, FALSE, FALSE, FALSE, FALSE, FALSE, FALSE,

FALSE, FALSE, FALSE, FALSE, FALSE, FALSE, FALSE, FALSE, FALSE,

FALSE, FALSE, FALSE, FALSE, FALSE, FALSE, FALSE, FALSE, FALSE,

FALSE, FALSE, FALSE, FALSE, FALSE, FALSE, FALSE, FALSE, FALSE,

FALSE), OUTCOMES_L2_IX_inflammatory_markers = c(FALSE, FALSE,

FALSE, FALSE, FALSE, FALSE, FALSE, FALSE, FALSE, FALSE, FALSE,

FALSE, FALSE, FALSE, FALSE, FALSE, TRUE, FALSE, FALSE, FALSE,

TRUE, FALSE, FALSE, FALSE, FALSE, FALSE, FALSE, FALSE, FALSE,

FALSE, FALSE, FALSE, FALSE, FALSE, FALSE, FALSE, FALSE, FALSE,

FALSE, FALSE, FALSE, FALSE, FALSE, FALSE, FALSE, FALSE, FALSE,

FALSE, FALSE, FALSE, FALSE, FALSE, FALSE, FALSE, FALSE, FALSE,

FALSE, FALSE, FALSE, FALSE, FALSE, FALSE, FALSE, TRUE, FALSE,

FALSE, FALSE, FALSE, FALSE, FALSE, FALSE, FALSE, FALSE, FALSE,

FALSE, FALSE, FALSE, FALSE, FALSE, FALSE, FALSE, FALSE, FALSE,

FALSE, FALSE, FALSE, FALSE, FALSE, TRUE, FALSE, FALSE, FALSE,

FALSE, FALSE, FALSE, FALSE, FALSE, FALSE, FALSE, FALSE, FALSE,

FALSE, FALSE, FALSE, FALSE, FALSE, FALSE, FALSE, FALSE, FALSE,

FALSE, FALSE, FALSE, FALSE, FALSE, FALSE, FALSE, FALSE, TRUE,

FALSE, FALSE, FALSE, FALSE, FALSE, FALSE, FALSE, FALSE, FALSE,

FALSE, FALSE, FALSE, FALSE, FALSE, FALSE, FALSE, FALSE, FALSE,

FALSE, FALSE), OUTCOMES_L2_IX_liver_function_tests = c(FALSE,

FALSE, FALSE, FALSE, FALSE, FALSE, FALSE, FALSE, FALSE, FALSE,

FALSE, FALSE, FALSE, FALSE, FALSE, FALSE, TRUE, FALSE, FALSE,

FALSE, FALSE, FALSE, FALSE, FALSE, FALSE, FALSE, FALSE, FALSE,

FALSE, FALSE, FALSE, FALSE, FALSE, FALSE, FALSE, FALSE, FALSE,

FALSE, FALSE, FALSE, FALSE, FALSE, FALSE, FALSE, FALSE, FALSE,

FALSE, FALSE, FALSE, FALSE, FALSE, FALSE, FALSE, FALSE, FALSE,

FALSE, FALSE, FALSE, FALSE, FALSE, FALSE, FALSE, FALSE, FALSE,

FALSE, FALSE, FALSE, FALSE, FALSE, FALSE, FALSE, FALSE, FALSE,

FALSE, FALSE, FALSE, FALSE, FALSE, FALSE, FALSE, FALSE, FALSE,

FALSE, FALSE, FALSE, FALSE, FALSE, FALSE, TRUE, FALSE, FALSE,

FALSE, FALSE, FALSE, FALSE, FALSE, FALSE, FALSE, FALSE, FALSE,

FALSE, FALSE, FALSE, FALSE, FALSE, FALSE, FALSE, FALSE, FALSE,

FALSE, FALSE, FALSE, FALSE, FALSE, FALSE, FALSE, FALSE, FALSE,

FALSE, FALSE, FALSE, FALSE, FALSE, FALSE, FALSE, FALSE, FALSE,

FALSE, FALSE, FALSE, FALSE, FALSE, FALSE, FALSE, FALSE, FALSE,

FALSE, FALSE, FALSE), OUTCOMES_L2_IX_procalcitonin = c(FALSE,

FALSE, FALSE, FALSE, FALSE, FALSE, FALSE, FALSE, FALSE, FALSE,

FALSE, FALSE, FALSE, FALSE, FALSE, FALSE, TRUE, FALSE, FALSE,

FALSE, FALSE, FALSE, FALSE, FALSE, FALSE, FALSE, FALSE, FALSE,

FALSE, FALSE, FALSE, FALSE, FALSE, FALSE, FALSE, FALSE, FALSE,

FALSE, FALSE, FALSE, FALSE, FALSE, FALSE, FALSE, FALSE, FALSE,

FALSE, FALSE, FALSE, FALSE, FALSE, FALSE, FALSE, FALSE, FALSE,

FALSE, FALSE, FALSE, FALSE, FALSE, FALSE, FALSE, FALSE, FALSE,

FALSE, FALSE, FALSE, FALSE, FALSE, FALSE, FALSE, FALSE, FALSE,

FALSE, FALSE, FALSE, FALSE, FALSE, FALSE, FALSE, FALSE, FALSE,

FALSE, FALSE, FALSE, FALSE, FALSE, FALSE, FALSE, FALSE, FALSE,

FALSE, FALSE, FALSE, FALSE, FALSE, FALSE, FALSE, FALSE, FALSE,

FALSE, FALSE, FALSE, FALSE, FALSE, FALSE, FALSE, FALSE, FALSE,

FALSE, FALSE, FALSE, FALSE, FALSE, FALSE, FALSE, FALSE, FALSE,

FALSE, FALSE, FALSE, FALSE, FALSE, FALSE, FALSE, FALSE, FALSE,

FALSE, FALSE, FALSE, FALSE, FALSE, FALSE, FALSE, FALSE, FALSE,

FALSE, FALSE, FALSE), OUTCOMES_L2_IX_urea_electrolytes = c(FALSE,

FALSE, FALSE, FALSE, FALSE, FALSE, FALSE, FALSE, FALSE, FALSE,

FALSE, FALSE, FALSE, FALSE, FALSE, FALSE, TRUE, FALSE, FALSE,

FALSE, FALSE, FALSE, FALSE, FALSE, FALSE, FALSE, FALSE, FALSE,

FALSE, FALSE, FALSE, FALSE, FALSE, FALSE, FALSE, FALSE, FALSE,

FALSE, FALSE, FALSE, FALSE, FALSE, FALSE, FALSE, FALSE, FALSE,

FALSE, FALSE, FALSE, FALSE, FALSE, TRUE, FALSE, FALSE, FALSE,

FALSE, FALSE, FALSE, FALSE, FALSE, FALSE, FALSE, FALSE, FALSE,

FALSE, FALSE, FALSE, FALSE, FALSE, FALSE, FALSE, FALSE, FALSE,

FALSE, FALSE, FALSE, FALSE, FALSE, FALSE, FALSE, FALSE, FALSE,

FALSE, FALSE, FALSE, FALSE, FALSE, FALSE, TRUE, FALSE, FALSE,

FALSE, FALSE, FALSE, FALSE, FALSE, FALSE, FALSE, FALSE, FALSE,

FALSE, FALSE, FALSE, FALSE, FALSE, FALSE, FALSE, FALSE, FALSE,

FALSE, FALSE, FALSE, FALSE, FALSE, FALSE, FALSE, FALSE, FALSE,

FALSE, FALSE, FALSE, FALSE, FALSE, FALSE, FALSE, FALSE, FALSE,

FALSE, FALSE, FALSE, FALSE, FALSE, FALSE, FALSE, FALSE, FALSE,

FALSE, FALSE, FALSE), OUTCOMES_L2_IX_white_cell_count = c(FALSE,

FALSE, FALSE, FALSE, FALSE, FALSE, FALSE, FALSE, FALSE, FALSE,

FALSE, FALSE, FALSE, FALSE, FALSE, FALSE, TRUE, FALSE, FALSE,

FALSE, TRUE, FALSE, FALSE, FALSE, FALSE, FALSE, FALSE, FALSE,

FALSE, FALSE, FALSE, FALSE, FALSE, FALSE, FALSE, FALSE, FALSE,

FALSE, FALSE, FALSE, FALSE, FALSE, FALSE, FALSE, FALSE, FALSE,

FALSE, FALSE, FALSE, FALSE, FALSE, FALSE, FALSE, FALSE, FALSE,

FALSE, FALSE, FALSE, FALSE, FALSE, FALSE, FALSE, FALSE, TRUE,

FALSE, FALSE, FALSE, FALSE, FALSE, FALSE, FALSE, FALSE, TRUE,

FALSE, FALSE, FALSE, FALSE, TRUE, FALSE, FALSE, FALSE, FALSE,

FALSE, FALSE, FALSE, FALSE, TRUE, TRUE, TRUE, FALSE, FALSE, FALSE,

FALSE, FALSE, FALSE, FALSE, FALSE, FALSE, FALSE, FALSE, FALSE,

FALSE, FALSE, FALSE, FALSE, FALSE, TRUE, FALSE, FALSE, FALSE,

FALSE, FALSE, FALSE, FALSE, FALSE, FALSE, FALSE, FALSE, TRUE,

FALSE, FALSE, FALSE, TRUE, FALSE, FALSE, FALSE, FALSE, FALSE,

FALSE, FALSE, FALSE, FALSE, FALSE, FALSE, FALSE, FALSE, FALSE,

FALSE, FALSE), OUTCOMES_L2_COMPLICATIONS_cardiac_complications = c(FALSE,

FALSE, FALSE, FALSE, FALSE, TRUE, FALSE, FALSE, FALSE, FALSE,

FALSE, FALSE, FALSE, FALSE, FALSE, TRUE, TRUE, FALSE, FALSE,

FALSE, TRUE, TRUE, FALSE, FALSE, FALSE, FALSE, FALSE, FALSE,

FALSE, FALSE, FALSE, FALSE, FALSE, FALSE, FALSE, FALSE, FALSE,

FALSE, FALSE, FALSE, FALSE, FALSE, FALSE, FALSE, FALSE, FALSE,

FALSE, FALSE, TRUE, TRUE, FALSE, TRUE, FALSE, FALSE, FALSE, FALSE,

FALSE, FALSE, FALSE, FALSE, FALSE, FALSE, FALSE, FALSE, FALSE,

FALSE, FALSE, FALSE, FALSE, FALSE, FALSE, FALSE, FALSE, TRUE,

FALSE, FALSE, FALSE, FALSE, FALSE, FALSE, FALSE, FALSE, FALSE,

FALSE, FALSE, TRUE, FALSE, FALSE, FALSE, FALSE, FALSE, FALSE,

FALSE, FALSE, FALSE, FALSE, FALSE, FALSE, FALSE, FALSE, FALSE,

FALSE, FALSE, FALSE, FALSE, FALSE, FALSE, FALSE, FALSE, TRUE,

FALSE, FALSE, FALSE, FALSE, FALSE, FALSE, FALSE, FALSE, FALSE,

FALSE, FALSE, FALSE, FALSE, FALSE, FALSE, FALSE, FALSE, FALSE,

FALSE, FALSE, FALSE, FALSE, FALSE, FALSE, FALSE, FALSE, FALSE,

FALSE, FALSE), OUTCOMES_L2_COMPLICATIONS_neurological_complications = c(FALSE,

FALSE, FALSE, FALSE, FALSE, TRUE, FALSE, FALSE, FALSE, FALSE,

FALSE, FALSE, FALSE, FALSE, FALSE, FALSE, TRUE, FALSE, FALSE,

FALSE, TRUE, FALSE, FALSE, FALSE, FALSE, FALSE, FALSE, TRUE,

FALSE, TRUE, TRUE, FALSE, FALSE, FALSE, FALSE, FALSE, FALSE,

FALSE, FALSE, FALSE, FALSE, FALSE, FALSE, FALSE, FALSE, FALSE,

FALSE, FALSE, TRUE, TRUE, FALSE, TRUE, FALSE, FALSE, FALSE, FALSE,

FALSE, FALSE, FALSE, FALSE, FALSE, FALSE, FALSE, FALSE, FALSE,

FALSE, FALSE, FALSE, FALSE, FALSE, FALSE, FALSE, FALSE, TRUE,

FALSE, FALSE, FALSE, FALSE, FALSE, FALSE, FALSE, FALSE, FALSE,

FALSE, FALSE, FALSE, FALSE, FALSE, FALSE, FALSE, FALSE, FALSE,

FALSE, FALSE, FALSE, FALSE, FALSE, FALSE, FALSE, FALSE, FALSE,

FALSE, FALSE, FALSE, FALSE, FALSE, FALSE, FALSE, FALSE, TRUE,

FALSE, FALSE, FALSE, FALSE, FALSE, FALSE, FALSE, FALSE, FALSE,

FALSE, FALSE, FALSE, FALSE, FALSE, FALSE, FALSE, FALSE, FALSE,

FALSE, FALSE, FALSE, FALSE, FALSE, FALSE, FALSE, FALSE, FALSE,

FALSE, FALSE), OUTCOMES_L2_COMPLICATIONS_organ_failure = c(FALSE,

FALSE, FALSE, FALSE, FALSE, TRUE, FALSE, FALSE, FALSE, FALSE,

FALSE, FALSE, FALSE, FALSE, FALSE, FALSE, FALSE, FALSE, FALSE,

FALSE, FALSE, FALSE, FALSE, FALSE, FALSE, FALSE, FALSE, FALSE,

FALSE, FALSE, FALSE, FALSE, FALSE, FALSE, FALSE, FALSE, FALSE,

FALSE, FALSE, FALSE, FALSE, FALSE, FALSE, FALSE, FALSE, FALSE,

FALSE, FALSE, FALSE, FALSE, FALSE, FALSE, FALSE, FALSE, FALSE,

FALSE, FALSE, FALSE, FALSE, FALSE, FALSE, FALSE, FALSE, FALSE,

TRUE, FALSE, FALSE, FALSE, FALSE, FALSE, FALSE, FALSE, FALSE,

FALSE, FALSE, FALSE, FALSE, FALSE, FALSE, FALSE, FALSE, FALSE,

FALSE, FALSE, FALSE, FALSE, FALSE, FALSE, FALSE, FALSE, FALSE,

FALSE, FALSE, FALSE, FALSE, FALSE, FALSE, FALSE, FALSE, FALSE,

FALSE, FALSE, FALSE, FALSE, FALSE, FALSE, FALSE, FALSE, FALSE,

TRUE, FALSE, FALSE, FALSE, FALSE, FALSE, FALSE, FALSE, FALSE,

FALSE, FALSE, FALSE, FALSE, FALSE, FALSE, FALSE, FALSE, FALSE,

FALSE, FALSE, TRUE, FALSE, FALSE, FALSE, FALSE, FALSE, FALSE,

FALSE, FALSE, FALSE), OUTCOMES_L2_COMPLICATIONS_respiratory_complications = c(FALSE,

FALSE, FALSE, FALSE, FALSE, TRUE, FALSE, FALSE, FALSE, FALSE,

FALSE, FALSE, FALSE, FALSE, FALSE, TRUE, TRUE, FALSE, FALSE,

FALSE, TRUE, TRUE, FALSE, TRUE, FALSE, FALSE, FALSE, FALSE, FALSE,

FALSE, FALSE, FALSE, FALSE, FALSE, FALSE, TRUE, FALSE, FALSE,

FALSE, FALSE, FALSE, FALSE, FALSE, FALSE, FALSE, FALSE, FALSE,

FALSE, TRUE, TRUE, FALSE, TRUE, FALSE, FALSE, FALSE, FALSE, FALSE,

FALSE, FALSE, FALSE, FALSE, FALSE, FALSE, FALSE, FALSE, FALSE,

FALSE, FALSE, FALSE, FALSE, FALSE, FALSE, FALSE, FALSE, FALSE,

FALSE, FALSE, FALSE, FALSE, FALSE, FALSE, FALSE, FALSE, FALSE,

FALSE, TRUE, FALSE, FALSE, FALSE, TRUE, TRUE, TRUE, TRUE, FALSE,

FALSE, FALSE, FALSE, FALSE, TRUE, FALSE, FALSE, FALSE, FALSE,

FALSE, FALSE, FALSE, FALSE, FALSE, FALSE, TRUE, FALSE, FALSE,

FALSE, FALSE, FALSE, FALSE, FALSE, FALSE, FALSE, FALSE, FALSE,

TRUE, FALSE, FALSE, FALSE, FALSE, FALSE, FALSE, FALSE, FALSE,

FALSE, FALSE, FALSE, FALSE, FALSE, FALSE, FALSE, FALSE, FALSE

), OUTCOMES_L2_COMPLICATIONS_renal_complications = c(FALSE, FALSE,

FALSE, FALSE, FALSE, FALSE, FALSE, FALSE, FALSE, FALSE, FALSE,

FALSE, FALSE, FALSE, FALSE, TRUE, TRUE, FALSE, FALSE, FALSE,

FALSE, FALSE, FALSE, FALSE, FALSE, FALSE, FALSE, FALSE, FALSE,

FALSE, FALSE, FALSE, FALSE, FALSE, FALSE, FALSE, FALSE, FALSE,

FALSE, FALSE, FALSE, FALSE, FALSE, FALSE, FALSE, FALSE, FALSE,

FALSE, FALSE, FALSE, FALSE, TRUE, FALSE, FALSE, FALSE, FALSE,

FALSE, FALSE, FALSE, FALSE, FALSE, FALSE, FALSE, FALSE, FALSE,

FALSE, FALSE, FALSE, FALSE, FALSE, FALSE, FALSE, FALSE, FALSE,

FALSE, FALSE, FALSE, FALSE, FALSE, FALSE, FALSE, FALSE, FALSE,

FALSE, FALSE, FALSE, FALSE, FALSE, FALSE, TRUE, TRUE, FALSE,

FALSE, FALSE, FALSE, FALSE, FALSE, FALSE, FALSE, FALSE, FALSE,

FALSE, FALSE, FALSE, FALSE, FALSE, FALSE, FALSE, FALSE, FALSE,

FALSE, FALSE, FALSE, FALSE, FALSE, FALSE, FALSE, FALSE, FALSE,

FALSE, FALSE, FALSE, FALSE, FALSE, FALSE, FALSE, FALSE, FALSE,

FALSE, FALSE, FALSE, FALSE, FALSE, FALSE, FALSE, FALSE, FALSE,

FALSE, FALSE), OUTCOMES_L2_COMPLICATIONS_sepsis = c(FALSE, FALSE,

FALSE, FALSE, FALSE, FALSE, FALSE, FALSE, FALSE, FALSE, FALSE,

FALSE, FALSE, FALSE, FALSE, FALSE, FALSE, FALSE, FALSE, FALSE,

FALSE, FALSE, FALSE, FALSE, FALSE, FALSE, FALSE, FALSE, FALSE,

FALSE, FALSE, FALSE, FALSE, FALSE, FALSE, FALSE, FALSE, FALSE,

FALSE, FALSE, FALSE, FALSE, FALSE, FALSE, FALSE, FALSE, FALSE,

FALSE, FALSE, FALSE, FALSE, FALSE, FALSE, FALSE, FALSE, FALSE,

FALSE, FALSE, FALSE, FALSE, FALSE, FALSE, FALSE, FALSE, FALSE,

TRUE, FALSE, FALSE, FALSE, FALSE, FALSE, FALSE, FALSE, TRUE,

FALSE, FALSE, FALSE, FALSE, FALSE, FALSE, FALSE, FALSE, FALSE,

FALSE, FALSE, FALSE, TRUE, FALSE, FALSE, FALSE, FALSE, FALSE,

FALSE, FALSE, FALSE, FALSE, FALSE, FALSE, FALSE, FALSE, FALSE,

FALSE, FALSE, FALSE, FALSE, FALSE, FALSE, FALSE, FALSE, TRUE,

FALSE, FALSE, FALSE, FALSE, FALSE, FALSE, FALSE, FALSE, FALSE,

FALSE, FALSE, FALSE, FALSE, FALSE, FALSE, FALSE, FALSE, FALSE,

FALSE, FALSE, FALSE, FALSE, FALSE, FALSE, FALSE, FALSE, FALSE,

FALSE, FALSE), OUTCOMES_L2_COMPLICATIONS_shock = c(FALSE, FALSE,

FALSE, FALSE, FALSE, FALSE, FALSE, FALSE, FALSE, FALSE, FALSE,

FALSE, FALSE, FALSE, FALSE, FALSE, FALSE, FALSE, FALSE, FALSE,

FALSE, FALSE, FALSE, FALSE, FALSE, FALSE, FALSE, FALSE, FALSE,

FALSE, FALSE, FALSE, FALSE, FALSE, FALSE, FALSE, FALSE, FALSE,

FALSE, FALSE, FALSE, FALSE, FALSE, FALSE, FALSE, FALSE, FALSE,

FALSE, FALSE, FALSE, FALSE, TRUE, FALSE, FALSE, FALSE, FALSE,

FALSE, FALSE, FALSE, FALSE, FALSE, FALSE, FALSE, FALSE, FALSE,

FALSE, FALSE, FALSE, FALSE, FALSE, FALSE, FALSE, FALSE, FALSE,

FALSE, FALSE, FALSE, FALSE, FALSE, FALSE, FALSE, FALSE, FALSE,

FALSE, FALSE, FALSE, FALSE, FALSE, FALSE, FALSE, FALSE, FALSE,

FALSE, FALSE, FALSE, FALSE, FALSE, FALSE, FALSE, FALSE, FALSE,

FALSE, FALSE, FALSE, FALSE, FALSE, FALSE, FALSE, FALSE, FALSE,

FALSE, FALSE, FALSE, FALSE, FALSE, FALSE, FALSE, FALSE, FALSE,

FALSE, FALSE, FALSE, FALSE, FALSE, FALSE, FALSE, FALSE, FALSE,

FALSE, FALSE, FALSE, FALSE, FALSE, FALSE, FALSE, FALSE, FALSE,

FALSE, FALSE), OUTCOMES_L2_TREATMENT_antibiotics = c(FALSE, FALSE,

FALSE, FALSE, FALSE, FALSE, FALSE, TRUE, FALSE, FALSE, FALSE,

FALSE, FALSE, FALSE, FALSE, FALSE, FALSE, FALSE, FALSE, FALSE,

TRUE, FALSE, FALSE, FALSE, FALSE, FALSE, FALSE, FALSE, FALSE,

FALSE, FALSE, FALSE, FALSE, FALSE, FALSE, FALSE, FALSE, FALSE,

FALSE, FALSE, FALSE, FALSE, FALSE, FALSE, FALSE, FALSE, FALSE,

TRUE, TRUE, TRUE, FALSE, FALSE, FALSE, FALSE, TRUE, FALSE, FALSE,

FALSE, FALSE, FALSE, FALSE, FALSE, FALSE, FALSE, TRUE, TRUE,

FALSE, FALSE, FALSE, FALSE, FALSE, FALSE, TRUE, TRUE, FALSE,

FALSE, FALSE, TRUE, FALSE, FALSE, FALSE, FALSE, TRUE, FALSE,

FALSE, FALSE, TRUE, FALSE, TRUE, FALSE, FALSE, FALSE, FALSE,

FALSE, FALSE, FALSE, FALSE, FALSE, FALSE, FALSE, FALSE, FALSE,

FALSE, FALSE, FALSE, FALSE, FALSE, FALSE, FALSE, FALSE, FALSE,

FALSE, FALSE, FALSE, FALSE, FALSE, TRUE, FALSE, TRUE, FALSE,

FALSE, FALSE, TRUE, FALSE, FALSE, FALSE, TRUE, FALSE, FALSE,

TRUE, FALSE, FALSE, FALSE, FALSE, FALSE, FALSE, FALSE, FALSE,

FALSE), OUTCOMES_L2_TREATMENT_antivirals = c(FALSE, FALSE, FALSE,

FALSE, FALSE, TRUE, FALSE, FALSE, FALSE, FALSE, FALSE, FALSE,

FALSE, FALSE, FALSE, FALSE, FALSE, FALSE, FALSE, FALSE, TRUE,

FALSE, TRUE, FALSE, FALSE, FALSE, FALSE, FALSE, FALSE, FALSE,

FALSE, TRUE, TRUE, TRUE, FALSE, FALSE, FALSE, FALSE, FALSE, FALSE,

FALSE, TRUE, TRUE, FALSE, FALSE, FALSE, FALSE, FALSE, FALSE,

FALSE, FALSE, FALSE, FALSE, FALSE, FALSE, FALSE, FALSE, FALSE,

FALSE, FALSE, FALSE, FALSE, FALSE, FALSE, TRUE, FALSE, TRUE,

FALSE, FALSE, FALSE, FALSE, FALSE, FALSE, FALSE, FALSE, FALSE,

TRUE, FALSE, FALSE, TRUE, FALSE, FALSE, FALSE, FALSE, FALSE,

FALSE, TRUE, TRUE, FALSE, TRUE, TRUE, FALSE, FALSE, FALSE, FALSE,

FALSE, FALSE, FALSE, FALSE, FALSE, FALSE, FALSE, FALSE, FALSE,

FALSE, FALSE, FALSE, FALSE, FALSE, FALSE, TRUE, FALSE, FALSE,

FALSE, FALSE, FALSE, FALSE, FALSE, FALSE, FALSE, FALSE, FALSE,

TRUE, TRUE, FALSE, FALSE, FALSE, FALSE, FALSE, FALSE, FALSE,

FALSE, TRUE, TRUE, FALSE, FALSE, FALSE, FALSE, FALSE), OUTCOMES_L2_TREATMENT_steroids = c(FALSE,

FALSE, FALSE, FALSE, FALSE, FALSE, FALSE, FALSE, FALSE, FALSE,

FALSE, FALSE, FALSE, FALSE, FALSE, FALSE, FALSE, FALSE, FALSE,

FALSE, FALSE, FALSE, FALSE, FALSE, FALSE, FALSE, FALSE, FALSE,

FALSE, FALSE, FALSE, FALSE, FALSE, FALSE, FALSE, FALSE, FALSE,

FALSE, FALSE, FALSE, FALSE, FALSE, FALSE, FALSE, FALSE, FALSE,

FALSE, FALSE, FALSE, FALSE, FALSE, FALSE, FALSE, FALSE, FALSE,

FALSE, FALSE, FALSE, FALSE, FALSE, FALSE, FALSE, FALSE, FALSE,

FALSE, FALSE, FALSE, FALSE, FALSE, FALSE, FALSE, FALSE, FALSE,

FALSE, FALSE, FALSE, FALSE, FALSE, FALSE, FALSE, FALSE, FALSE,

FALSE, FALSE, FALSE, FALSE, FALSE, FALSE, FALSE, FALSE, FALSE,

FALSE, FALSE, FALSE, FALSE, FALSE, FALSE, FALSE, FALSE, FALSE,

FALSE, FALSE, FALSE, FALSE, FALSE, FALSE, FALSE, FALSE, FALSE,

FALSE, FALSE, FALSE, FALSE, FALSE, TRUE, FALSE, FALSE, FALSE,

FALSE, FALSE, FALSE, FALSE, FALSE, FALSE, FALSE, FALSE, FALSE,

FALSE, FALSE, FALSE, FALSE, FALSE, FALSE, FALSE, FALSE, FALSE,

FALSE, FALSE, FALSE), OUTCOMES_L2_HOSPITAL_hospital_admission = c(FALSE,

FALSE, FALSE, TRUE, FALSE, FALSE, FALSE, FALSE, FALSE, FALSE,

FALSE, FALSE, FALSE, TRUE, FALSE, FALSE, TRUE, FALSE, TRUE, FALSE,

TRUE, FALSE, FALSE, FALSE, TRUE, FALSE, FALSE, TRUE, FALSE, TRUE,

TRUE, FALSE, FALSE, FALSE, FALSE, FALSE, TRUE, FALSE, FALSE,

FALSE, FALSE, FALSE, FALSE, FALSE, FALSE, FALSE, FALSE, FALSE,

FALSE, FALSE, FALSE, FALSE, FALSE, FALSE, TRUE, FALSE, FALSE,

FALSE, FALSE, FALSE, TRUE, FALSE, FALSE, FALSE, TRUE, FALSE,

FALSE, FALSE, FALSE, FALSE, FALSE, FALSE, FALSE, FALSE, FALSE,

FALSE, FALSE, FALSE, FALSE, FALSE, FALSE, FALSE, FALSE, FALSE,

FALSE, FALSE, TRUE, FALSE, TRUE, TRUE, TRUE, FALSE, FALSE, FALSE,

FALSE, FALSE, FALSE, FALSE, FALSE, FALSE, FALSE, FALSE, TRUE,

TRUE, TRUE, TRUE, TRUE, FALSE, FALSE, TRUE, FALSE, FALSE, FALSE,

FALSE, FALSE, FALSE, TRUE, FALSE, TRUE, FALSE, FALSE, FALSE,

TRUE, TRUE, FALSE, FALSE, FALSE, FALSE, FALSE, FALSE, FALSE,

FALSE, FALSE, FALSE, FALSE, FALSE, FALSE, FALSE, FALSE), OUTCOMES_L2_HOSPITAL_hospital_attendance = c(FALSE,

FALSE, FALSE, FALSE, FALSE, FALSE, FALSE, FALSE, FALSE, FALSE,

FALSE, FALSE, FALSE, FALSE, FALSE, FALSE, FALSE, FALSE, FALSE,

FALSE, FALSE, FALSE, FALSE, FALSE, FALSE, FALSE, FALSE, FALSE,

FALSE, FALSE, FALSE, FALSE, FALSE, FALSE, FALSE, FALSE, FALSE,

FALSE, FALSE, FALSE, FALSE, FALSE, FALSE, FALSE, FALSE, FALSE,

FALSE, FALSE, FALSE, FALSE, FALSE, FALSE, FALSE, FALSE, FALSE,

FALSE, FALSE, FALSE, FALSE, FALSE, FALSE, FALSE, FALSE, FALSE,

FALSE, FALSE, FALSE, FALSE, FALSE, FALSE, FALSE, FALSE, FALSE,

FALSE, FALSE, FALSE, FALSE, FALSE, FALSE, FALSE, FALSE, FALSE,

FALSE, FALSE, FALSE, FALSE, FALSE, FALSE, FALSE, FALSE, FALSE,

FALSE, FALSE, FALSE, FALSE, FALSE, FALSE, FALSE, FALSE, FALSE,

FALSE, FALSE, FALSE, FALSE, FALSE, FALSE, TRUE, FALSE, FALSE,

FALSE, FALSE, FALSE, FALSE, FALSE, FALSE, FALSE, FALSE, FALSE,

TRUE, FALSE, FALSE, FALSE, FALSE, FALSE, FALSE, FALSE, FALSE,

FALSE, FALSE, FALSE, FALSE, FALSE, FALSE, FALSE, FALSE, FALSE,

FALSE, FALSE, FALSE), OUTCOMES_L2_HOSPITAL_hospital_attendance_advised = c(FALSE,

FALSE, FALSE, FALSE, FALSE, FALSE, FALSE, FALSE, FALSE, FALSE,

FALSE, FALSE, FALSE, FALSE, FALSE, FALSE, FALSE, FALSE, FALSE,

FALSE, TRUE, FALSE, FALSE, FALSE, FALSE, FALSE, FALSE, FALSE,

FALSE, FALSE, FALSE, FALSE, FALSE, FALSE, FALSE, FALSE, FALSE,

FALSE, FALSE, FALSE, FALSE, FALSE, FALSE, FALSE, FALSE, FALSE,

FALSE, FALSE, FALSE, FALSE, FALSE, FALSE, FALSE, FALSE, FALSE,

FALSE, FALSE, FALSE, FALSE, FALSE, FALSE, FALSE, FALSE, TRUE,

FALSE, FALSE, FALSE, FALSE, FALSE, FALSE, FALSE, FALSE, TRUE,

FALSE, FALSE, FALSE, FALSE, TRUE, FALSE, FALSE, FALSE, FALSE,

FALSE, FALSE, FALSE, FALSE, TRUE, FALSE, TRUE, FALSE, FALSE,

FALSE, FALSE, FALSE, FALSE, FALSE, FALSE, FALSE, FALSE, FALSE,

FALSE, FALSE, FALSE, FALSE, FALSE, FALSE, FALSE, FALSE, FALSE,

FALSE, FALSE, FALSE, FALSE, FALSE, FALSE, FALSE, FALSE, FALSE,

FALSE, FALSE, FALSE, FALSE, FALSE, FALSE, FALSE, FALSE, FALSE,

FALSE, FALSE, FALSE, FALSE, FALSE, FALSE, FALSE, FALSE, FALSE,

FALSE, FALSE, FALSE), OUTCOMES_L2_HOSPITAL_iv_fluids = c(FALSE,

FALSE, FALSE, TRUE, FALSE, TRUE, TRUE, FALSE, TRUE, TRUE, TRUE,

FALSE, FALSE, FALSE, TRUE, FALSE, FALSE, FALSE, FALSE, TRUE,

FALSE, FALSE, TRUE, FALSE, FALSE, TRUE, FALSE, FALSE, TRUE, TRUE,

FALSE, FALSE, TRUE, FALSE, FALSE, FALSE, FALSE, FALSE, FALSE,

FALSE, FALSE, FALSE, FALSE, FALSE, FALSE, FALSE, FALSE, FALSE,

TRUE, FALSE, FALSE, FALSE, FALSE, TRUE, FALSE, TRUE, FALSE, FALSE,

FALSE, FALSE, FALSE, FALSE, FALSE, TRUE, TRUE, TRUE, TRUE, FALSE,

FALSE, FALSE, FALSE, FALSE, FALSE, FALSE, TRUE, TRUE, TRUE, FALSE,

FALSE, TRUE, FALSE, FALSE, FALSE, FALSE, FALSE, FALSE, FALSE,

TRUE, TRUE, TRUE, FALSE, FALSE, FALSE, FALSE, FALSE, FALSE, FALSE,

TRUE, TRUE, FALSE, TRUE, FALSE, TRUE, FALSE, FALSE, TRUE, FALSE,

FALSE, FALSE, FALSE, FALSE, FALSE, FALSE, FALSE, FALSE, FALSE,

FALSE, FALSE, TRUE, FALSE, FALSE, FALSE, FALSE, FALSE, FALSE,

FALSE, FALSE, FALSE, FALSE, FALSE, TRUE, FALSE, TRUE, TRUE, FALSE,

TRUE, FALSE, TRUE, FALSE), OUTCOMES_L2_HOSPITAL_o2_therapy = c(FALSE,

FALSE, FALSE, FALSE, FALSE, FALSE, FALSE, FALSE, FALSE, TRUE,

TRUE, FALSE, FALSE, FALSE, FALSE, FALSE, FALSE, FALSE, FALSE,

FALSE, FALSE, FALSE, FALSE, FALSE, FALSE, FALSE, FALSE, FALSE,

FALSE, FALSE, FALSE, FALSE, FALSE, FALSE, FALSE, FALSE, FALSE,

FALSE, FALSE, FALSE, FALSE, FALSE, FALSE, FALSE, FALSE, FALSE,

FALSE, FALSE, FALSE, FALSE, FALSE, FALSE, FALSE, FALSE, FALSE,

FALSE, FALSE, FALSE, TRUE, FALSE, FALSE, FALSE, FALSE, FALSE,

FALSE, FALSE, FALSE, FALSE, FALSE, FALSE, FALSE, TRUE, FALSE,

FALSE, FALSE, FALSE, FALSE, FALSE, FALSE, FALSE, FALSE, FALSE,

FALSE, TRUE, FALSE, FALSE, FALSE, FALSE, FALSE, FALSE, FALSE,

FALSE, FALSE, FALSE, FALSE, FALSE, FALSE, FALSE, FALSE, FALSE,

FALSE, FALSE, FALSE, FALSE, FALSE, FALSE, FALSE, FALSE, FALSE,

FALSE, FALSE, FALSE, FALSE, FALSE, FALSE, FALSE, FALSE, FALSE,

FALSE, FALSE, FALSE, FALSE, FALSE, FALSE, FALSE, FALSE, FALSE,

FALSE, FALSE, FALSE, FALSE, FALSE, FALSE, FALSE, FALSE, FALSE,

FALSE, FALSE, FALSE), OUTCOMES_L2_HOSPITAL_duration_o2_therapy = c(FALSE,

FALSE, FALSE, FALSE, FALSE, FALSE, FALSE, FALSE, FALSE, TRUE,

FALSE, FALSE, FALSE, FALSE, FALSE, FALSE, FALSE, FALSE, FALSE,

FALSE, FALSE, FALSE, FALSE, FALSE, FALSE, FALSE, FALSE, FALSE,

FALSE, FALSE, FALSE, FALSE, FALSE, FALSE, FALSE, FALSE, FALSE,

FALSE, FALSE, FALSE, FALSE, FALSE, FALSE, FALSE, FALSE, FALSE,

FALSE, FALSE, FALSE, FALSE, FALSE, FALSE, FALSE, FALSE, FALSE,

FALSE, FALSE, FALSE, TRUE, FALSE, FALSE, FALSE, FALSE, FALSE,

FALSE, FALSE, FALSE, FALSE, FALSE, FALSE, FALSE, TRUE, FALSE,

FALSE, FALSE, FALSE, FALSE, FALSE, FALSE, FALSE, FALSE, FALSE,

FALSE, FALSE, FALSE, FALSE, FALSE, FALSE, FALSE, FALSE, FALSE,

FALSE, FALSE, FALSE, FALSE, FALSE, FALSE, FALSE, FALSE, FALSE,

FALSE, FALSE, FALSE, FALSE, FALSE, FALSE, FALSE, FALSE, FALSE,

FALSE, FALSE, FALSE, FALSE, FALSE, FALSE, FALSE, FALSE, FALSE,

FALSE, FALSE, FALSE, FALSE, FALSE, FALSE, FALSE, FALSE, FALSE,

FALSE, FALSE, FALSE, FALSE, FALSE, FALSE, FALSE, FALSE, FALSE,

FALSE, FALSE, FALSE), OUTCOMES_L2_HOSPITAL_hospital_length_of_stay = c(FALSE,

FALSE, FALSE, FALSE, FALSE, FALSE, FALSE, TRUE, TRUE, FALSE,

FALSE, FALSE, FALSE, TRUE, FALSE, TRUE, TRUE, FALSE, TRUE, FALSE,

TRUE, TRUE, FALSE, FALSE, FALSE, TRUE, FALSE, TRUE, FALSE, FALSE,

FALSE, FALSE, TRUE, FALSE, FALSE, FALSE, TRUE, FALSE, FALSE,

FALSE, TRUE, FALSE, TRUE, FALSE, FALSE, FALSE, FALSE, TRUE, FALSE,

FALSE, FALSE, TRUE, FALSE, FALSE, TRUE, TRUE, FALSE, TRUE, FALSE,

FALSE, FALSE, FALSE, FALSE, FALSE, FALSE, FALSE, FALSE, FALSE,

TRUE, FALSE, FALSE, FALSE, TRUE, TRUE, FALSE, FALSE, FALSE, TRUE,

FALSE, FALSE, FALSE, FALSE, TRUE, FALSE, FALSE, TRUE, TRUE, FALSE,

FALSE, FALSE, TRUE, FALSE, FALSE, TRUE, FALSE, FALSE, TRUE, FALSE,

FALSE, FALSE, FALSE, FALSE, FALSE, TRUE, TRUE, FALSE, TRUE, FALSE,

FALSE, FALSE, TRUE, FALSE, FALSE, FALSE, TRUE, FALSE, TRUE, FALSE,

TRUE, FALSE, FALSE, FALSE, TRUE, FALSE, FALSE, FALSE, TRUE, FALSE,

TRUE, FALSE, TRUE, FALSE, FALSE, FALSE, TRUE, FALSE, FALSE, FALSE,

FALSE), OUTCOMES_L2_ICU_icu_admission = c(FALSE, TRUE, FALSE,

TRUE, FALSE, TRUE, FALSE, FALSE, TRUE, FALSE, FALSE, TRUE, FALSE,

TRUE, FALSE, TRUE, FALSE, TRUE, TRUE, FALSE, TRUE, FALSE, FALSE,

TRUE, FALSE, FALSE, TRUE, TRUE, FALSE, FALSE, FALSE, FALSE, FALSE,

TRUE, TRUE, TRUE, TRUE, FALSE, FALSE, FALSE, FALSE, TRUE, FALSE,

FALSE, FALSE, FALSE, FALSE, TRUE, FALSE, FALSE, FALSE, TRUE,

FALSE, FALSE, TRUE, TRUE, TRUE, TRUE, FALSE, FALSE, TRUE, FALSE,

FALSE, TRUE, FALSE, FALSE, TRUE, TRUE, TRUE, FALSE, TRUE, FALSE,

TRUE, FALSE, FALSE, FALSE, FALSE, FALSE, TRUE, FALSE, FALSE,

TRUE, TRUE, FALSE, FALSE, TRUE, TRUE, FALSE, TRUE, FALSE, TRUE,

TRUE, FALSE, FALSE, FALSE, FALSE, TRUE, TRUE, TRUE, FALSE, FALSE,

FALSE, FALSE, TRUE, TRUE, FALSE, TRUE, FALSE, TRUE, TRUE, TRUE,

TRUE, TRUE, FALSE, TRUE, FALSE, TRUE, FALSE, FALSE, TRUE, FALSE,

TRUE, TRUE, TRUE, TRUE, FALSE, TRUE, FALSE, FALSE, FALSE, TRUE,

FALSE, FALSE, FALSE, TRUE, TRUE, FALSE, FALSE, TRUE), OUTCOMES_L2_ICU_icu_length_of_stay = c(FALSE,

FALSE, FALSE, FALSE, FALSE, FALSE, FALSE, FALSE, FALSE, FALSE,

FALSE, FALSE, FALSE, FALSE, FALSE, FALSE, FALSE, FALSE, FALSE,

FALSE, FALSE, FALSE, FALSE, FALSE, FALSE, FALSE, FALSE, FALSE,

FALSE, FALSE, FALSE, FALSE, FALSE, FALSE, FALSE, FALSE, FALSE,

FALSE, FALSE, FALSE, FALSE, FALSE, FALSE, FALSE, FALSE, FALSE,

FALSE, FALSE, FALSE, FALSE, FALSE, FALSE, FALSE, FALSE, FALSE,

FALSE, FALSE, FALSE, FALSE, FALSE, FALSE, FALSE, FALSE, FALSE,

FALSE, FALSE, FALSE, FALSE, FALSE, FALSE, FALSE, FALSE, FALSE,

FALSE, FALSE, FALSE, FALSE, FALSE, FALSE, FALSE, FALSE, FALSE,

FALSE, FALSE, FALSE, FALSE, TRUE, FALSE, FALSE, FALSE, FALSE,

FALSE, TRUE, FALSE, FALSE, FALSE, FALSE, FALSE, FALSE, FALSE,

FALSE, FALSE, FALSE, FALSE, FALSE, FALSE, FALSE, FALSE, FALSE,

TRUE, FALSE, FALSE, FALSE, FALSE, FALSE, FALSE, FALSE, FALSE,

FALSE, FALSE, FALSE, FALSE, FALSE, FALSE, FALSE, FALSE, FALSE,

FALSE, FALSE, TRUE, FALSE, FALSE, FALSE, FALSE, FALSE, FALSE,

TRUE, FALSE, FALSE), OUTCOMES_L2_ICU_ventilation = c(FALSE, TRUE,

FALSE, TRUE, FALSE, TRUE, FALSE, FALSE, TRUE, FALSE, FALSE, TRUE,

FALSE, FALSE, FALSE, TRUE, TRUE, TRUE, FALSE, FALSE, TRUE, FALSE,

FALSE, TRUE, TRUE, TRUE, FALSE, TRUE, FALSE, FALSE, TRUE, TRUE,

FALSE, TRUE, FALSE, FALSE, TRUE, FALSE, FALSE, FALSE, FALSE,

TRUE, FALSE, FALSE, FALSE, FALSE, FALSE, TRUE, FALSE, FALSE,

FALSE, FALSE, FALSE, FALSE, TRUE, TRUE, TRUE, TRUE, FALSE, FALSE,

TRUE, FALSE, FALSE, FALSE, FALSE, FALSE, FALSE, TRUE, FALSE,

FALSE, FALSE, FALSE, TRUE, FALSE, FALSE, FALSE, FALSE, FALSE,

FALSE, FALSE, FALSE, TRUE, TRUE, FALSE, FALSE, FALSE, TRUE, FALSE,

TRUE, FALSE, TRUE, FALSE, TRUE, FALSE, FALSE, FALSE, FALSE, FALSE,

FALSE, FALSE, FALSE, FALSE, FALSE, FALSE, FALSE, FALSE, TRUE,

FALSE, FALSE, TRUE, TRUE, FALSE, TRUE, FALSE, FALSE, FALSE, TRUE,

TRUE, FALSE, TRUE, FALSE, FALSE, TRUE, TRUE, FALSE, FALSE, TRUE,

FALSE, FALSE, TRUE, TRUE, FALSE, TRUE, FALSE, FALSE, TRUE, TRUE,

FALSE, TRUE), OUTCOMES_L2_ICU_duration_ventilation = c(FALSE,

FALSE, FALSE, FALSE, FALSE, FALSE, FALSE, FALSE, FALSE, FALSE,

FALSE, FALSE, FALSE, FALSE, FALSE, FALSE, FALSE, FALSE, FALSE,

FALSE, FALSE, FALSE, FALSE, FALSE, FALSE, FALSE, FALSE, FALSE,

FALSE, FALSE, FALSE, FALSE, FALSE, FALSE, FALSE, FALSE, FALSE,

FALSE, FALSE, FALSE, FALSE, FALSE, FALSE, FALSE, FALSE, FALSE,

FALSE, FALSE, FALSE, FALSE, FALSE, FALSE, FALSE, FALSE, FALSE,

FALSE, FALSE, TRUE, FALSE, FALSE, FALSE, FALSE, FALSE, FALSE,

FALSE, FALSE, FALSE, FALSE, FALSE, FALSE, FALSE, FALSE, FALSE,

FALSE, FALSE, FALSE, FALSE, FALSE, FALSE, FALSE, FALSE, FALSE,

FALSE, FALSE, FALSE, FALSE, FALSE, FALSE, FALSE, FALSE, FALSE,

FALSE, FALSE, FALSE, FALSE, FALSE, FALSE, FALSE, FALSE, FALSE,

FALSE, FALSE, FALSE, FALSE, FALSE, FALSE, TRUE, FALSE, FALSE,

FALSE, FALSE, FALSE, FALSE, FALSE, FALSE, FALSE, FALSE, FALSE,

FALSE, FALSE, FALSE, FALSE, FALSE, FALSE, FALSE, FALSE, FALSE,

FALSE, FALSE, FALSE, FALSE, FALSE, FALSE, FALSE, FALSE, FALSE,

FALSE, FALSE, FALSE), OUTCOMES_L2_ICU_inotropes = c(FALSE, FALSE,

FALSE, TRUE, FALSE, FALSE, FALSE, FALSE, FALSE, FALSE, FALSE,

FALSE, FALSE, FALSE, FALSE, FALSE, TRUE, FALSE, FALSE, FALSE,

FALSE, FALSE, FALSE, FALSE, FALSE, FALSE, FALSE, FALSE, FALSE,

FALSE, FALSE, FALSE, FALSE, FALSE, FALSE, FALSE, FALSE, FALSE,

FALSE, FALSE, FALSE, FALSE, FALSE, FALSE, FALSE, FALSE, FALSE,

FALSE, FALSE, FALSE, FALSE, FALSE, FALSE, FALSE, FALSE, FALSE,

FALSE, FALSE, FALSE, FALSE, FALSE, FALSE, FALSE, FALSE, FALSE,

FALSE, FALSE, FALSE, FALSE, FALSE, FALSE, FALSE, FALSE, FALSE,

FALSE, FALSE, FALSE, FALSE, FALSE, FALSE, FALSE, FALSE, FALSE,

FALSE, FALSE, FALSE, FALSE, FALSE, FALSE, FALSE, FALSE, FALSE,

FALSE, FALSE, FALSE, FALSE, FALSE, FALSE, FALSE, FALSE, FALSE,

FALSE, FALSE, FALSE, FALSE, FALSE, FALSE, FALSE, FALSE, FALSE,

FALSE, FALSE, FALSE, FALSE, FALSE, FALSE, FALSE, FALSE, FALSE,

FALSE, FALSE, FALSE, FALSE, FALSE, FALSE, FALSE, FALSE, FALSE,

FALSE, FALSE, FALSE, FALSE, FALSE, FALSE, FALSE, FALSE, FALSE,

FALSE, FALSE), OUTCOMES_L2_ICU_ecmo = c(FALSE, FALSE, FALSE,

FALSE, FALSE, TRUE, FALSE, FALSE, FALSE, FALSE, FALSE, FALSE,

FALSE, FALSE, FALSE, FALSE, FALSE, FALSE, FALSE, FALSE, FALSE,

FALSE, FALSE, FALSE, FALSE, FALSE, FALSE, FALSE, FALSE, FALSE,

TRUE, FALSE, FALSE, TRUE, FALSE, FALSE, FALSE, FALSE, FALSE,

FALSE, FALSE, FALSE, FALSE, FALSE, FALSE, FALSE, FALSE, FALSE,

FALSE, FALSE, FALSE, FALSE, FALSE, FALSE, FALSE, FALSE, FALSE,

FALSE, FALSE, FALSE, FALSE, FALSE, FALSE, FALSE, FALSE, FALSE,

FALSE, FALSE, FALSE, FALSE, FALSE, FALSE, FALSE, FALSE, FALSE,

FALSE, FALSE, FALSE, FALSE, FALSE, FALSE, FALSE, FALSE, FALSE,

FALSE, FALSE, FALSE, FALSE, FALSE, FALSE, TRUE, FALSE, FALSE,

FALSE, FALSE, FALSE, FALSE, FALSE, FALSE, FALSE, FALSE, FALSE,

FALSE, FALSE, FALSE, FALSE, FALSE, FALSE, FALSE, FALSE, FALSE,

FALSE, FALSE, FALSE, FALSE, FALSE, FALSE, FALSE, FALSE, FALSE,

FALSE, TRUE, FALSE, TRUE, TRUE, FALSE, FALSE, FALSE, FALSE, FALSE,

FALSE, FALSE, FALSE, FALSE, FALSE, FALSE, FALSE, FALSE, FALSE

)), class = "data.frame", row.names = c(NA, -139L))}

###############################################################

# Optional: export to CSV for convenience

###############################################################

write.csv(data, file = "appendix_data.csv", row.names = FALSE)
