# Supplementary material for: A Systematic Review of the Markers of Severity in Acute Respiratory Infections to Inform Primary Care Surveillance
Source: Influenza Other Respir Viruses. 2025 Oct 24;19(10):e70172. doi: 10.1111/irv.70172 (PMC12550406; doi:10.1111/irv.70172)
Supplement: Supplementary file 2 — Data S2: Supporting information. [file IRV-19-e70172-s002.docx]

# APPENDIX

**Appendix 1**: Database(s): Global Health 1973 to 2023 Week 23
Search Strategy.

| # | Searches | Results |
| --- | --- | --- |
| 1 | surveillance/ or sentinel surveillance/ or syndromic surveillance/ | 29423 |
| 2 | (ARI or SARI or ILI).mp. [mp=abstract, title, original title, heading words, cabicodes words] | 4924 |
| 3 | "surveillance indicator*".mp. or surveillance.in. | 10225 |
| 4 | ("public health effect*" or "epidemiological effect*").mp. | 364 |
| 5 | 1 or 2 or 3 or 4 | 43049 |
| 6 | exp influenza/ or influenza.mp. | 47257 |
| 7 | SARS-CoV-2/ or COVID-19/ | 111070 |
| 8 | (corona* adj1 (virus* or viral*)).mp. [mp=abstract, title, original title, heading words, cabicodes words] | 2093 |
| 9 | (CoV not (Coefficien* or "co-efficien*" or covalent* or Covington* or covariant* or covarianc* or "cut-off value*" or "cutoff value*" or "cut-off volume*" or "cutoff volume*" or "combined optimi?ation value*" or "central vessel trunk*" or CoVR or CoVS)).ti,ab. | 46438 |
| 10 | (coronavirus* or 2019nCoV* or 19nCoV* or "2019 novel*" or Ncov* or "n-cov" or "SARS- CoV-2*" or "SARSCoV-2*" or SARSCoV2* or "SARS-CoV2*" or COVID*2).mp. [mp=abstract, title, original title, heading words, cabicodes words] | 126133 |
| 11 | "severe acute respiratory syndrome".mp. | 110444 |
| 12 | 7 or 8 or 9 or 10 or 11 | 126595 |
| 13 | limit 12 to yr="2020-current" | 120492 |
| 14 | 6 or 13 | 163906 |
| 15 | ("hospitali?ation rat*" or "fatality rat*" or "mortality rat*" or "death rat*" or "SARI:ARI" or "SARI:ILI").mp. [mp=abstract, title, original title, heading words, cabicodes words] | 184226 |
| 16 | (sever* or "seriousness").ti. | 39616 |
| 17 | ("clinical* sever*" or "clinical* serious*").mp. [mp=abstract, title, original title, heading words, cabicodes words] | 2278 |
| 18 | ((sever* or serious*) adj2 (infection? or disease? or outcome? or endpoint?)).mp. [mp=abstract, title, original title, heading words, cabicodes words] | 71038 |
| 19 | 15 or 16 or 17 or 18 | 270359 |
| 20 | 5 and 14 and 19 | 1732 |
| 21 | exp animals/ not humans/ | 720712 |
| 22 | 20 not 21 | 1701 |
| 23 | limit 22 to yr="2009-current" | 1616 |
| 24 | limit 23 to english language | 1486 |

**Appendix 2:** Database(s): Embase 1974 to present Search Strategy.

| # | Searches | Results |
| --- | --- | --- |
| 1 | community based surveillance/ or epidemiological surveillance/ | 1613 |
| 2 | (ARI or SARI or ILI).ti,ab,kf. | 11313 |
| 3 | "surveillance indicator*".ti,ab,kf. or surveillance.in. | 32987 |
| 4 | ("public health effect*" or "epidemiological effect*").ti,ab,kf. | 719 |
| 5 | 1 or 2 or 3 or 4 | 46300 |
| 6 | exp *influenza/ | 56430 |
| 7 | influenza.ti,ab. | 129765 |
| 8 | (corona* adj1 (virus* or viral*)).ti,ab,kf. | 6062 |
| 9 | (CoV not (Coefficien* or "co-efficien*" or covalent* or Covington* or covariant* or covarianc* or "cut-off value*" or "cutoff value*" or "cut-off volume*" or "cutoff volume*" or "combined optimi?ation value*" or "central vessel trunk*" or CoVR or CoVS)).ti,ab,kf. | 151936 |
| 10 | (coronavirus* or 2019nCoV* or 19nCoV* or "2019 novel*" or Ncov* or "n-cov" or "SARS- CoV-2*" or "SARSCoV-2*" or SARSCoV2* or "SARS-CoV2*" or "severe acute respiratory syndrome*" or COVID*2).ti,ab,kf. | 456528 |
| 11 | exp *severe acute respiratory syndrome/ or *severe acute respiratory syndrome coronavirus 2/ or coronavirus disease 2019/ | 383741 |
| 12 | 8 or 9 or 10 or 11 | 486040 |
| 13 | limit 12 to yr="2020-current" | 469303 |
| 14 | 6 or 7 or 13 | 597126 |
| 15 | ("hospitali?ation rat*" or "fatality rat*" or "mortality rat*" or "death rat*" or "SARI:ARI" or "SARI:ILI").ti,ab,kf. | 321556 |
| 16 | (severe or severity).ti. | 323591 |
| 17 | ("clinical* sever*" or "clinical* serious*").ti,ab,kf. | 19920 |
| 18 | ((sever* or serious*) adj2 (infection? or disease? or outcome? or endpoint?)).ti,ab,kf. | 351980 |
| 19 | 15 or 16 or 17 or 18 | 944751 |
| 20 | 5 and 14 and 19 | 1299 |
| 21 | exp animal/ not human/ | 5333940 |
| 22 | 20 not 21 | 1288 |
| 23 | limit 22 to yr="2009-current" | 1260 |
| 24 | limit 23 to english language | 1213 |

**Appendix 3:** Database(s): Medline (Ovid MEDLINE® Epub Ahead of Print, In-Process & Other Non-Indexed Citations, Ovid MEDLINE® Daily and Ovid MEDLINE®) 1946 to present
Search Strategy.

| # | Searches | Results |
| --- | --- | --- |
| 1 | Sentinel Surveillance/ or Public Health Surveillance/ | 12077 |
| 2 | (ARI or SARI or ILI).ti,ab,kf. | 8064 |
| 3 | "surveillance indicator*".ti,ab,kf. or surveillance.in. | 21946 |
| 4 | ("public health effect*" or "epidemiological effect*").ti,ab,kf. | 678 |
| 5 | 1 or 2 or 3 or 4 | 41720 |
| 6 | influenza.ti,ab,kf. or Influenza, Human/ | 122865 |
| 7 | SARS-CoV-2/ or COVID-19/ | 233242 |
| 8 | (corona* adj1 (virus* or viral*)).ti,ab,kf. | 4442 |
| 9 | (CoV not (Coefficien* or "co-efficien*" or covalent* or Covington* or covariant* or covarianc* or "cut-off value*" or "cutoff value*" or "cut-off volume*" or "cutoff volume*" or "combined optimi?ation value*" or "central vessel trunk*" or CoVR or CoVS)).ti,ab. | 106972 |
| 10 | (coronavirus* or 2019nCoV* or 19nCoV* or "2019 novel*" or Ncov* or "n-cov" or "SARS- CoV-2*" or "SARSCoV-2*" or SARSCoV2* or "SARS-CoV2*" or COVID*2).ti,ab,kf. | 370567 |
| 11 | "severe acute respiratory syndrome".ti,ab,kf. | 42896 |
| 12 | 7 or 8 or 9 or 10 or 11 | 380183 |
| 13 | limit 12 to yr="2020-current" | 366869 |
| 14 | 6 or 13 | 481854 |
| 15 | ("hospitali?ation rat*" or "fatality rat*" or "mortality rat*" or "death rat*" or "SARI:ARI" or "SARI:ILI").ti,ab,kf. | 224265 |
| 16 | (sever* or "seriousness").ti. | 269411 |
| 17 | ("clinical* sever*" or "clinical* serious*").ti,ab,kf. | 13163 |
| 18 | ((sever* or serious*) adj2 (infection? or disease? or outcome? or endpoint?)).ti,ab,kf. | 234861 |
| 19 | 15 or 16 or 17 or 18 | 693052 |
| 20 | 5 and 14 and 19 | 1015 |
| 21 | exp animals/ not humans/ | 5130390 |
| 22 | 20 not 21 | 1008 |
| 23 | limit 22 to yr="2009-current" | 984 |
| 24 | limit 23 to english language | 954 |

**Appendix 4:** Grey-literature search procedure (15 March 2024)

To complement database searching, five public-health web portals were searched for surveillance reports published between 1 January 2018 and 31 December 2023 that contained human respiratory-virus surveillance data.

1. **United States CDC**: we ran the query “respiratory virus surveillance reports” in the agency’s global search box. We screened all returned titles and snippets page-by-page until no new relevant items appeared.

(https://search.cdc.gov/search/?query=Respiratory%20virus%20surveillance%20reports&dpage=1)

1. **WHO Disease Outbreak News:** we scrolled the archive retaining only those that described routine human respiratory-virus surveillance; avian- or animal-only influenza posts were excluded and screened the first 300 notices

(https://www.who.int/emergencies/disease-outbreak-news)

1. **UK Health Security Agency (UKHSA):** using the GOV.UK site search, we reviewed hits sorted by relevance and selected documents explicitly labelled as national respiratory-virus surveillance bulletins.

(https://www.gov.uk/search/all?keywords=Respiratory+virus+surveillance+reports)

1. **ECDC epidemiological updates:** on the ECDC monitoring page we opened and screened the 20 most recent update entries for human respiratory-virus content.

([**https://www.ecdc.europa.eu/en/publications-data/monitoring/epidemiological-**](https://www.ecdc.europa.eu/en/publications-data/monitoring/epidemiological-)updates)

1. **Sentiworld sentinel-network portal:** we accessed each primary-care sentinel system listed on ***https://www.sentiworld.info*** and manually browsed their linked publications pages for weekly or seasonal respiratory-virus surveillance reports.
2. For every source, titles, executive summaries, and (where available) PDF bulletins were examined; items that met the inclusion criteria were exported as PDF.

**Appendix 5:** Data items collected.

| Category | Data element |
| --- | --- |
| Study Details | Study title |
|  | Study aim |
|  | Publication type and date |
|  | Study period (start and end date) |
|  | Geographical scope (country(s) and region(s)) |
| Case Information | Case types:   1. ARI 2. ILI 3. SARI 4. Suspected COVID 5. Other (if felt that the criteria were met e.g. MAARI) |
|  | Case definitions used: e.g., WHO or CDC. |
|  | Number of cases |
|  | Case recruitment type:   1. Hospitalised 2. Treatment-seeking: patients recruited in outpatient settings including: primary care, emergency department and outpatient clinics. 3. Non-treatment-seeking: individuals recruited in the community, but not attending healthcare settings. E.g., community surveys. 4. Intensive Care 5. Mixed |
| Severity markers | Outcomes that could represent possible severity markers |
|  | Severity marker group: (e.g., clinical scores, symptoms, signs, lab parameters, complications, treatments, hospitalisation, death other) |
|  | Severity marker type:   1. A severe outcome (e.g., death, complication or hospitalisation). 2. A possible predictor of an severe outcome (e.g., clinical signs or investigations) |
| Quality Assessment | Study setting and location description |
|  | Eligible population description |
|  | Data sources description |
|  | Selection bias discussion |
|  | Case definition clarity |
|  | Outcome clarity |
| Timeliness | Whether the timeliness of outcomes was reported |
|  | Comments on timeliness |

**Appendix 6:** Fully study summary

See separate excel file

**Appendix 7**: Study count by year showing bi-modal peak around swine flu and COVID19 pandemic.


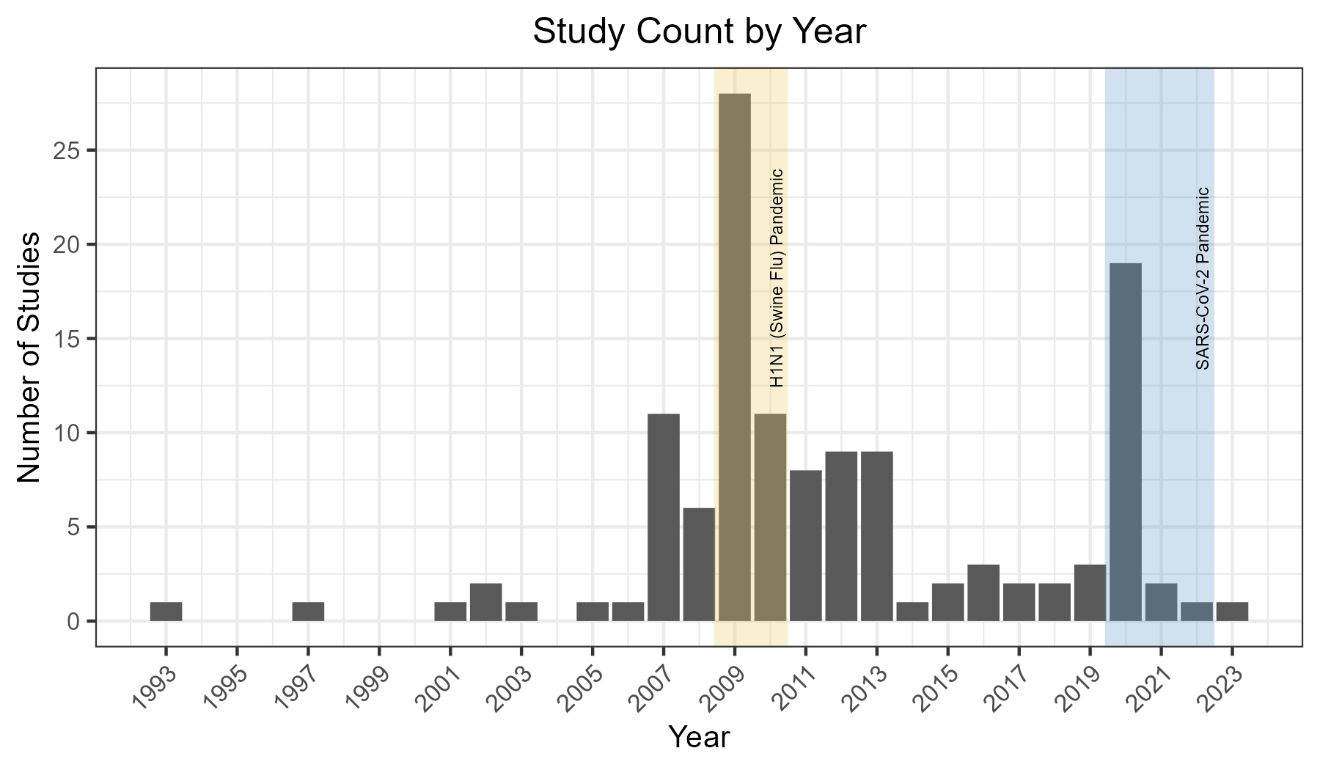


Appendix 7: The study counts by year. Yellow shaded are represents the H1N1 Influenza pandemic. World Health Organization (WHO) declared a Public health emergencies of international concern (PHEIC) on 25th of April 2009 that ended on 10th of August 2010 [2]. Blue shaded area represents the Severe Acute Respiratory Syndrome Coronavirus 2 (SARS-CoV-2) Pandemic, WHO declared a PHEIC on the 30th of January 2020 that ended on 5th of May 2023 [2,3]. The start and end date of shaded bars are rounded to the nearest year.

**Appendix 8:** Frequency of reporting of severity markers by recruitment type.
This table lists all severity markers extracted from included studies, stratified by recruitment type and grouped by clinical domain. Frequencies are reported as the number and percentage of studies in which each marker was reported. Acronyms are expanded for clarity:

- **AAP**: American Academy of Pediatrics
- **APACHE IV**: Acute Physiology and Chronic Health Evaluation IV
- **ECMO**: Extracorporeal membrane oxygenation
- **GCS**: Glasgow Coma Scale
- **HSB**: Health-seeking behaviour
- **ICU**: Intensive care unit
- **ISS**: Influenza Symptom Severity score
- **IV**: Intravenous
- **MEWS**: Modified Early Warning Score
- **PRISM III**: Pediatric Risk of Mortality III score
- **SAPS II**: Simplified Acute Physiology Score II
- **SOFA**: Sequential Organ Failure Assessment score
- **ViVI**: ViVI Disease Severity Score
- **WHO**: World Health Organization

| Recruitment type | Group | Severity marker | Frequency |
| --- | --- | --- | --- |
| All types | Death | Death | 100 (71.9%) |
| All types | ICU-related | ICU admission | 62 (44.6%) |
| All types | ICU-related | Ventilation | 48 (34.5%) |
| All types | Symptom | Cough | 47 (33.8%) |
| All types | Symptom | Shortness of breath | 42 (30.2%) |
| All types | Symptom | Fever | 41 (29.5%) |
| All types | Hospital | Hospital length of stay | 41 (29.5%) |
| All types | Hospital | IV fluids | 38 (27.3%) |
| All types | Symptom | Sorethroat | 36 (25.9%) |
| All types | Symptom | Coryza rhinorrea congestion | 30 (21.6%) |
| All types | Symptom | Myalgia arthralgia | 30 (21.6%) |
| All types | Symptom | Diarrhea | 27 (19.4%) |
| All types | Symptom | Nausea vomiting | 27 (19.4%) |
| All types | Hospital | Hospital admission | 27 (19.4%) |
| All types | Symptom | Malaise anorexia | 23 (16.6%) |
| All types | Treatment | Antivirals | 21 (15.1%) |
| All types | Treatment | Antibiotics | 19 (13.7%) |
| All types | Complications | Respiratory complications | 18 (12.9%) |
| All types | Signs | Temperature | 17 (12.2%) |
| All types | Symptom | Chest pain | 15 (10.8%) |
| All types | Signs | O2 saturation | 15 (10.8%) |
| All types | Signs | Respiratory rate | 14 (10.1%) |
| All types | Signs | Chest signs | 13 (9.3%) |
| All types | Symptom | Productive cough | 12 (8.6%) |
| All types | Investigations | White cell count | 11 (7.9%) |
| All types | Complications | Cardiac complications | 11 (7.9%) |
| All types | Complications | Neurological complications | 11 (7.9%) |
| All types | Symptom | Abdominal pain | 10 (7.2%) |
| All types | Score | Project specific composite | 10 (7.2%) |
| All types | Symptom | Length of illness | 9 (6.5%) |
| All types | Investigations | Chest xray | 9 (6.5%) |
| All types | Symptom | Convulsions | 8 (5.8%) |
| All types | Symptom | Loss of taste or smell | 8 (5.8%) |
| All types | Symptom | Haemoptysis | 7 (5.0%) |
| All types | Absence | Work absence | 7 (5.0%) |
| All types | Signs | Respiratory distress | 7 (5.0%) |
| All types | ICU-related | ECMO | 7 (5.0%) |
| All types | Signs | Pulse rate | 6 (4.3%) |
| All types | Hospital | Hospital attendance advised | 6 (4.3%) |
| All types | Absence | School absence | 5 (3.6%) |
| All types | Investigations | Inflammatory markers | 5 (3.6%) |
| All types | Complications | Renal complications | 5 (3.6%) |
| All types | Hospital | O2 therapy | 5 (3.6%) |
| All types | ICU-related | ICU length of stay | 5 (3.6%) |
| All types | Signs | Blood pressure | 4 (2.9%) |
| All types | Signs | Cyanosis | 4 (2.9%) |
| All types | Complications | Organ failure | 4 (2.9%) |
| All types | Complications | Sepsis | 4 (2.9%) |
| All types | Death | HSB | 3 (2.2%) |
| All types | Symptom | Confusion | 3 (2.2%) |
| All types | Symptom | Ear pain | 3 (2.2%) |
| All types | Score | Sofa score | 3 (2.2%) |
| All types | Investigations | Urea electrolytes | 3 (2.2%) |
| All types | Hospital | Duration o2 therapy | 3 (2.2%) |
| All types | Score | Who severity | 2 (1.4%) |
| All types | Investigations | Liver function tests | 2 (1.4%) |
| All types | Hospital | Hospital attendance | 2 (1.4%) |
| All types | ICU-related | Duration ventilation | 2 (1.4%) |
| All types | ICU-related | Inotropes | 2 (1.4%) |
| All types | Symptom | Irritability | 1 (0.7%) |
| All types | Symptom | Retroocular pain | 1 (0.7%) |
| All types | Score | American academy pediatrics guideline criteria | 1 (0.7%) |
| All types | Score | Apache iv | 1 (0.7%) |
| All types | Score | Barthel index | 1 (0.7%) |
| All types | Score | Euroqol | 1 (0.7%) |
| All types | Score | Gcs | 1 (0.7%) |
| All types | Score | Iss | 1 (0.7%) |
| All types | Score | Mews | 1 (0.7%) |
| All types | Score | Paediatric chinese medical association | 1 (0.7%) |
| All types | Score | Pediatric risk of mortality iii score | 1 (0.7%) |
| All types | Score | Saps 2 score | 1 (0.7%) |
| All types | Score | ViVI score | 1 (0.7%) |
| All types | Investigations | Arterial blood gas | 1 (0.7%) |
| All types | Investigations | Fibronogen | 1 (0.7%) |
| All types | Investigations | Procalcitonin | 1 (0.7%) |
| All types | Complications | Shock | 1 (0.7%) |
| All types | Treatment | Steroids | 1 (0.7%) |
| Hospitalised | Death | Death | 63 (87.5%) |
| Hospitalised | ICU-related | ICU admission | 51 (70.8%) |
| Hospitalised | Hospital | Hospital length of stay | 36 (50.0%) |
| Hospitalised | Icu-related | Ventilation | 34 (47.2%) |
| Hospitalised | Symptom | Cough | 23 (31.9%) |
| Hospitalised | Symptom | Shortness of breath | 21 (29.2%) |
| Hospitalised | Symptom | Fever | 19 (26.4%) |
| Hospitalised | Hospital | Hospital admission | 18 (25.0%) |
| Hospitalised | Symptom | Sorethroat | 15 (20.8%) |
| Hospitalised | Symptom | Coryza rhinorrea congestion | 13 (18.1%) |
| Hospitalised | Treatment | Antibiotics | 13 (18.1%) |
| Hospitalised | Complications | Respiratory complications | 12 (16.7%) |
| Hospitalised | Symptom | Myalgia arthralgia | 11 (15.3%) |
| Hospitalised | Signs | Chest signs | 10 (13.9%) |
| Hospitalised | Signs | Temperature | 10 (13.9%) |
| Hospitalised | Symptom | Diarrhea | 9 (12.5%) |
| Hospitalised | Symptom | Nausea vomiting | 9 (12.5%) |
| Hospitalised | Treatment | Antivirals | 9 (12.5%) |
| Hospitalised | Symptom | Malaise anorexia | 8 (11.1%) |
| Hospitalised | Signs | Respiratory rate | 8 (11.1%) |
| Hospitalised | Investigations | Chest xray | 8 (11.1%) |
| Hospitalised | Complications | Cardiac complications | 8 (11.1%) |
| Hospitalised | Complications | Neurological complications | 7 (9.7%) |
| Hospitalised | Symptom | Productive cough | 6 (8.3%) |
| Hospitalised | Investigations | White cell count | 6 (8.3%) |
| Hospitalised | ICU-related | ECMO | 6 (8.3%) |
| Hospitalised | Symptom | Chest pain | 5 (6.9%) |
| Hospitalised | Symptom | Convulsions | 5 (6.9%) |
| Hospitalised | Signs | O2 saturation | 5 (6.9%) |
| Hospitalised | Hospital | Hospital attendance advised | 4 (5.6%) |
| Hospitalised | Symptom | Abdominal pain | 3 (4.2%) |
| Hospitalised | Symptom | Haemoptysis | 3 (4.2%) |
| Hospitalised | Score | Project specific composite | 3 (4.2%) |
| Hospitalised | Complications | Renal complications | 3 (4.2%) |
| Hospitalised | Complications | Sepsis | 3 (4.2%) |
| Hospitalised | Symptom | Length of illness | 2 (2.8%) |
| Hospitalised | Symptom | Loss of taste or smell | 2 (2.8%) |
| Hospitalised | Signs | Cyanosis | 2 (2.8%) |
| Hospitalised | Signs | Pulse rate | 2 (2.8%) |
| Hospitalised | Signs | Respiratory distress | 2 (2.8%) |
| Hospitalised | Hospital | IV fluids | 2 (2.8%) |
| Hospitalised | ICU-related | ICU length of stay | 2 (2.8%) |
| Hospitalised | ICU-related | Duration ventilation | 2 (2.8%) |
| Hospitalised | Signs | Blood pressure | 1 (1.4%) |
| Hospitalised | Score | Barthel index | 1 (1.4%) |
| Hospitalised | Score | GCS | 1 (1.4%) |
| Hospitalised | Score | Pediatric risk of mortality iii score | 1 (1.4%) |
| Hospitalised | Score | Who severity | 1 (1.4%) |
| Hospitalised | Investigations | Inflammatory markers | 1 (1.4%) |
| Hospitalised | Investigations | Urea electrolytes | 1 (1.4%) |
| Hospitalised | Complications | Organ failure | 1 (1.4%) |
| Hospitalised | Complications | Shock | 1 (1.4%) |
| Hospitalised | Treatment | Steroids | 1 (1.4%) |
| Hospitalised | Hospital | Hospital attendance | 1 (1.4%) |
| Intensive care | Death | Death | 4 (100.0%) |
| Intensive care | ICU-related | ICU length of stay | 3 (75.0%) |
| Intensive care | ICU-related | Ventilation | 3 (75.0%) |
| Intensive care | Symptom | Cough | 2 (50.0%) |
| Intensive care | Symptom | Nausea vomiting | 2 (50.0%) |
| Intensive care | Symptom | Shortness of breath | 2 (50.0%) |
| Intensive care | Score | SOFA score | 2 (50.0%) |
| Intensive care | Symptom | Convulsions | 1 (25.0%) |
| Intensive care | Symptom | Coryza rhinorrea congestion | 1 (25.0%) |
| Intensive care | Symptom | Productive cough | 1 (25.0%) |
| Intensive care | Symptom | Diarrhea | 1 (25.0%) |
| Intensive care | Symptom | Fever | 1 (25.0%) |
| Intensive care | Symptom | Myalgia arthralgia | 1 (25.0%) |
| Intensive care | Symptom | Sorethroat | 1 (25.0%) |
| Intensive care | Signs | Chest signs | 1 (25.0%) |
| Intensive care | Score | APACHE IV | 1 (25.0%) |
| Intensive care | Score | SAPS 2 score | 1 (25.0%) |
| Intensive care | Investigations | Chest xray | 1 (25.0%) |
| Intensive care | Complications | Organ failure | 1 (25.0%) |
| Intensive care | Complications | Respiratory complications | 1 (25.0%) |
| Intensive care | Treatment | Antibiotics | 1 (25.0%) |
| Non treatment seeking | Absence | Work absence | 5 (62.5%) |
| Non treatment seeking | Absence | School absence | 4 (50.0%) |
| Non treatment seeking | Symptom | Cough | 3 (37.5%) |
| Non treatment seeking | Symptom | Fever | 3 (37.5%) |
| Non treatment seeking | Symptom | Length of illness | 3 (37.5%) |
| Non treatment seeking | Symptom | Myalgia arthralgia | 3 (37.5%) |
| Non treatment seeking | Death | Death | 2 (25.0%) |
| Non treatment seeking | Death | HSB | 2 (25.0%) |
| Non treatment seeking | Symptom | Coryza rhinorrea congestion | 2 (25.0%) |
| Non treatment seeking | Symptom | Sorethroat | 2 (25.0%) |
| Non treatment seeking | Hospital | IV fluids | 2 (25.0%) |
| Non treatment seeking | Hospital | O2 therapy | 2 (25.0%) |
| Non treatment seeking | Symptom | Abdominal pain | 1 (12.5%) |
| Non treatment seeking | Symptom | Diarrhea | 1 (12.5%) |
| Non treatment seeking | Symptom | Malaise anorexia | 1 (12.5%) |
| Non treatment seeking | Symptom | Nausea vomiting | 1 (12.5%) |
| Non treatment seeking | Symptom | Shortness of breath | 1 (12.5%) |
| Non treatment seeking | Signs | O2 saturation | 1 (12.5%) |
| Non treatment seeking | Score | ISS | 1 (12.5%) |
| Non treatment seeking | Score | Paediatric chinese medical association | 1 (12.5%) |
| Non treatment seeking | Investigations | Fibronogen | 1 (12.5%) |
| Non treatment seeking | Investigations | Inflammatory markers | 1 (12.5%) |
| Non treatment seeking | Investigations | Liver function tests | 1 (12.5%) |
| Non treatment seeking | Investigations | Procalcitonin | 1 (12.5%) |
| Non treatment seeking | Investigations | Urea electrolytes | 1 (12.5%) |
| Non treatment seeking | Investigations | White cell count | 1 (12.5%) |
| Non treatment seeking | Complications | Cardiac complications | 1 (12.5%) |
| Non treatment seeking | Complications | Neurological complications | 1 (12.5%) |
| Non treatment seeking | Complications | Respiratory complications | 1 (12.5%) |
| Non treatment seeking | Complications | Renal complications | 1 (12.5%) |
| Non treatment seeking | Hospital | Hospital admission | 1 (12.5%) |
| Non treatment seeking | Hospital | Duration O_2_ therapy | 1 (12.5%) |
| Non treatment seeking | Hospital | Hospital length of stay | 1 (12.5%) |
| Non treatment seeking | Icu-related | Ventilation | 1 (12.5%) |
| Non treatment seeking | Icu-related | Inotropes | 1 (12.5%) |
| Treatment seeking | Hospital | IV fluids | 23 (67.7%) |
| Treatment seeking | Death | Death | 14 (41.2%) |
| Treatment seeking | Symptom | Cough | 13 (38.2%) |
| Treatment seeking | Symptom | Shortness of breath | 13 (38.2%) |
| Treatment seeking | Symptom | Diarrhea | 12 (35.3%) |
| Treatment seeking | Symptom | Fever | 12 (35.3%) |
| Treatment seeking | Symptom | Sorethroat | 11 (32.4%) |
| Treatment seeking | Symptom | Coryza rhinorrea congestion | 9 (26.5%) |
| Treatment seeking | Symptom | Myalgia arthralgia | 9 (26.5%) |
| Treatment seeking | Symptom | Nausea vomiting | 9 (26.5%) |
| Treatment seeking | Signs | O2 saturation | 9 (26.5%) |
| Treatment seeking | Symptom | Malaise anorexia | 8 (23.5%) |
| Treatment seeking | Hospital | Hospital admission | 8 (23.5%) |
| Treatment seeking | Treatment | Antivirals | 7 (20.6%) |
| Treatment seeking | Symptom | Chest pain | 6 (17.6%) |
| Treatment seeking | Signs | Temperature | 6 (17.6%) |
| Treatment seeking | Score | Project specific composite | 6 (17.6%) |
| Treatment seeking | Signs | Respiratory rate | 5 (14.7%) |
| Treatment seeking | Signs | Respiratory distress | 5 (14.7%) |
| Treatment seeking | Treatment | Antibiotics | 5 (14.7%) |
| Treatment seeking | Symptom | Productive cough | 4 (11.8%) |
| Treatment seeking | Signs | Pulse rate | 4 (11.8%) |
| Treatment seeking | Icu-related | ICU admission | 4 (11.8%) |
| Treatment seeking | Symptom | Haemoptysis | 3 (8.8%) |
| Treatment seeking | Symptom | Length of illness | 3 (8.8%) |
| Treatment seeking | Symptom | Loss of taste or smell | 3 (8.8%) |
| Treatment seeking | Signs | Blood pressure | 3 (8.8%) |
| Treatment seeking | Investigations | Inflammatory markers | 3 (8.8%) |
| Treatment seeking | Investigations | White cell count | 3 (8.8%) |
| Treatment seeking | Symptom | Abdominal pain | 2 (5.9%) |
| Treatment seeking | Symptom | Confusion | 2 (5.9%) |
| Treatment seeking | Symptom | Convulsions | 2 (5.9%) |
| Treatment seeking | Symptom | Ear pain | 2 (5.9%) |
| Treatment seeking | Signs | Chest signs | 2 (5.9%) |
| Treatment seeking | Complications | Neurological complications | 2 (5.9%) |
| Treatment seeking | Complications | Respiratory complications | 2 (5.9%) |
| Treatment seeking | Hospital | Hospital attendance advised | 2 (5.9%) |
| Treatment seeking | Hospital | O2 therapy | 2 (5.9%) |
| Treatment seeking | Hospital | Duration o2 therapy | 2 (5.9%) |
| Treatment seeking | Hospital | Hospital length of stay | 2 (5.9%) |
| Treatment seeking | ICU-related | Ventilation | 2 (5.9%) |
| Treatment seeking | Death | HSB | 1 (2.9%) |
| Treatment seeking | Absence | School absence | 1 (2.9%) |
| Treatment seeking | Absence | Work absence | 1 (2.9%) |
| Treatment seeking | Signs | Cyanosis | 1 (2.9%) |
| Treatment seeking | Score | American academy pediatrics guideline criteria | 1 (2.9%) |
| Treatment seeking | Score | MEWS | 1 (2.9%) |
| Treatment seeking | Score | SOFA score | 1 (2.9%) |
| Treatment seeking | Score | Vivl score | 1 (2.9%) |
| Treatment seeking | Score | Who severity | 1 (2.9%) |
| Treatment seeking | Investigations | Arterial blood gas | 1 (2.9%) |
| Treatment seeking | Investigations | Liver function tests | 1 (2.9%) |
| Treatment seeking | Investigations | Urea electrolytes | 1 (2.9%) |
| Treatment seeking | Complications | Cardiac complications | 1 (2.9%) |
| Treatment seeking | Complications | Organ failure | 1 (2.9%) |
| Treatment seeking | Complications | Renal complications | 1 (2.9%) |
| Treatment seeking | Complications | Sepsis | 1 (2.9%) |
| Treatment seeking | Hospital | Hospital attendance | 1 (2.9%) |
| Treatment seeking | ICU-related | Inotropes | 1 (2.9%) |
| Treatment seeking hospitalised | Death | Death | 11 (73.3%) |
| Treatment seeking hospitalised | Hospital | IV fluids | 10 (66.7%) |
| Treatment seeking hospitalised | ICU-related | Ventilation | 7 (46.7%) |
| Treatment seeking hospitalised | ICU-related | ICU admission | 6 (40.0%) |
| Treatment seeking hospitalised | Symptom | Nausea vomiting | 5 (33.3%) |
| Treatment seeking hospitalised | Symptom | Sorethroat | 5 (33.3%) |
| Treatment seeking hospitalised | Symptom | Coryza rhinorrea congestion | 4 (26.7%) |
| Treatment seeking hospitalised | Symptom | Cough | 4 (26.7%) |
| Treatment seeking hospitalised | Symptom | Fever | 4 (26.7%) |
| Treatment seeking hospitalised | Symptom | Malaise anorexia | 4 (26.7%) |
| Treatment seeking hospitalised | Symptom | Myalgia arthralgia | 4 (26.7%) |
| Treatment seeking hospitalised | Treatment | Antivirals | 4 (26.7%) |
| Treatment seeking hospitalised | Symptom | Abdominal pain | 3 (20.0%) |
| Treatment seeking hospitalised | Symptom | Diarrhea | 3 (20.0%) |
| Treatment seeking hospitalised | Symptom | Shortness of breath | 3 (20.0%) |
| Treatment seeking hospitalised | Symptom | Chest pain | 2 (13.3%) |
| Treatment seeking hospitalised | Symptom | Loss of taste or smell | 2 (13.3%) |
| Treatment seeking hospitalised | Complications | Respiratory complications | 2 (13.3%) |
| Treatment seeking hospitalised | Hospital | Hospital length of stay | 2 (13.3%) |
| Treatment seeking hospitalised | Symptom | Productive cough | 1 (6.7%) |
| Treatment seeking hospitalised | Symptom | Ear pain | 1 (6.7%) |
| Treatment seeking hospitalised | Symptom | Haemoptysis | 1 (6.7%) |
| Treatment seeking hospitalised | Symptom | Length of illness | 1 (6.7%) |
| Treatment seeking hospitalised | Symptom | Retroocular pain | 1 (6.7%) |
| Treatment seeking hospitalised | Absence | Work absence | 1 (6.7%) |
| Treatment seeking hospitalised | Signs | Temperature | 1 (6.7%) |
| Treatment seeking hospitalised | Score | EUROQOL | 1 (6.7%) |
| Treatment seeking hospitalised | Score | Project specific composite | 1 (6.7%) |
| Treatment seeking hospitalised | Complications | Cardiac complications | 1 (6.7%) |
| Treatment seeking hospitalised | Complications | Neurological complications | 1 (6.7%) |
| Treatment seeking hospitalised | Complications | Organ failure | 1 (6.7%) |
| Treatment seeking hospitalised | Hospital | O2 therapy | 1 (6.7%) |
| Treatment seeking hospitalised | ICU-related | ECMO | 1 (6.7%) |
| Unknown | Death | Death | 6 (100.0%) |
| Unknown | Symptom | Chest pain | 2 (33.3%) |
| Unknown | Symptom | Cough | 2 (33.3%) |
| Unknown | Symptom | Fever | 2 (33.3%) |
| Unknown | Symptom | Malaise anorexia | 2 (33.3%) |
| Unknown | Symptom | Myalgia arthralgia | 2 (33.3%) |
| Unknown | Symptom | Shortness of breath | 2 (33.3%) |
| Unknown | Symptom | Sorethroat | 2 (33.3%) |
| Unknown | Symptom | Abdominal pain | 1 (16.7%) |
| Unknown | Symptom | Confusion | 1 (16.7%) |
| Unknown | Symptom | Coryza rhinorrea congestion | 1 (16.7%) |
| Unknown | Symptom | Diarrhea | 1 (16.7%) |
| Unknown | Symptom | Irritability | 1 (16.7%) |
| Unknown | Symptom | Loss of taste or smell | 1 (16.7%) |
| Unknown | Symptom | Nausea vomiting | 1 (16.7%) |
| Unknown | Signs | Cyanosis | 1 (16.7%) |
| Unknown | Signs | Respiratory rate | 1 (16.7%) |
| Unknown | Investigations | White cell count | 1 (16.7%) |
| Unknown | Treatment | Antivirals | 1 (16.7%) |
| Unknown | Hospital | IV fluids | 1 (16.7%) |
| Unknown | ICU-related | ICU admission | 1 (16.7%) |
| Unknown | ICU-related | Ventilation | 1 (16.7%) |

Appendix 9: Fully referenced severity marker candidates with description and inclusion decision and rational.

| OUTCOME | DESCRIPTION | DECISION & RATIONALE |
| --- | --- | --- |
| SEVERE OUTCOMES | | |
| COMPLICATIONS | | |
| Respiratory complications (18/139, 13%) [17–31] | Any acute complication of the respiratory system including respiratory failure and ARDS. | **INCLUDED**: Severe outcome. ARI-specific (esp. respiratory failure). Recorded in CMR via hospital discharge summaries, so not timely. |
| Cardiac complications (11/139, 8%) [17–21, 24–26, 30, 33] | Any acute complication of the cardiovascular system including acute coronary syndrome (ACS) and acute heart failure. | **EXCLUDED**: Severe, although ACS would likely be recorded in the CMR, less specific for ARI and therefore excluded. Would be recorded from discharge summary and not be timely. |
| Neurological complications (11/139, 8%) [17, 19, 20, 24, 25, 30, 33, 39, 40] | Any acute complication of the nervous system including transient ischaemic attack and cerebrovascular accident TIA/CVA. | **EXCLUDED**: Severe, although TIA/CVA would likely be recorded in the CMR but is less specific for ARI and therefore excluded. Would be recorded from discharge summary and not be timely. |
| Renal complications (5/139, 4%) [18, 19, 25, 27] | Any acute renal complication including acute kidney injury (AKI). | **EXCLUDED**: Severe, although AKI would likely be recorded in the CMR, less specific for ARI and therefore excluded. Would be recorded from discharge summary and not be timely. |
| Organ failure (3/139, 3%)[17, 30, 44, 121] | Any other non-specific organ failure or multiple organ failure. | **EXCLUDED**: Severe but non-specific and less likely recorded in the CMR. If recorded not timely. |
| Sepsis (4/139, 3%) [30, 32–34] | Severe systemic infection including septic shock and systemic inflammatory response syndrome (SIRS). | **INCLUDED**: Severe outcome. Infection-related although not ARI-specific. Recorded in CMR via hospital discharge summaries, so not timely. |
| Haemodynamic shock (1/139, 1%) [25] | Acute circulatory failure. | **EXCLUDED**: Severe complication; Non-specific to ARI, therefore excluded. Recorded in the CMR from discharge summary therefore not timely |
| HOSPITAL | | |
| Hospital Admission (27/139, 19%) [19, 20, 27, 30, 34–52] | Emergency hospital admission for an ARI. | **INCLUDED**: Standard epidemiological severe outcome. In primary care CMRs, both an admission and a diagnosis (e.g., pneumonia) may be recorded, but they are not directly linked, making it difficult to define an ARI-specific admission. Admissions are captured via hospital discharge summaries, so reporting is not timely. |
| Hospital Attendance (2/139, 1%) [48, 50] | Emergency hospital attendance for an ARI. | **INCLUDED**: Standard epidemiological severe outcome. In primary care CMRs, both an attendance and a diagnosis (e.g., pneumonia) may be recorded, but they are not directly linked, so it is difficult to define an ARI-specific attendance. Attendances are captured via emergency department discharge summaries, so reporting is not timely. |
| Hospital Attendance Advised (6/139, 4%) [20, 34, 45, 53–55] | Emergency hospital attendance for an ARI advised. | Indicates higher concern by the clinician, as emergency attendance is recommended. More easily attributable to an ARI since the advice is usually recorded at the same time as the ARI consultation in the primary care CMR, so more timely than outcomes captured via hospital discharge summaries. However, such advice is less consistently recorded compared with admissions or attendances. |
| Length of hospital stay (41/139, 29%) [18–21, 24–27, 33, 34, 36, 37, 39, 41, 42, 46–51, 54, 55, 57, 60, 63, 66, 69, 71, 73, 76, 79, 82–84, 91, 94, 96, 110, 119, 134] | Duration of time spent in hospital for an ARI. | **EXCLUDED**: Not routinely recorded in the primary care CMR therefore excluded. |
| Oxygen administration 95/139, 4%) [128, 129, 131, 136, 137] | The administration of oxygen therapy | **EXCLUDED**: Not routinely recorded in the primary care CMR therefore excluded. |
| Length of oxygen administration (3/139, 2%) [129, 131, 137] | The total duration of oxygen therapy administered to a patient | **EXCLUDED**: Not routinely recorded in the primary care CMR therefore excluded. |
| Intravenous fluid administration (38/139, 27%) [17, 24, 27, 29, 32, 35, 40, 44–47, 50, 53, 57, 60, 63, 64, 74, 83, 85, 89–92, 99, 103, 104, 107, 112, 122, 127, 129, 135, 136, 138–141] | The administration of intravenous fluid therapy | **EXCLUDED**: Not routinely recorded in the primary care CMR therefore excluded. |
| INTENSIVE CARE | | |
| Intensive Care Unit Admission (ICU Admission) (62/139, 45%) [17, 18, 20, 22, 23, 25–31, 34–37, 39, 41–43, 45–49, 51–54, 56–86] | Emergency admission to the Intensive Care Unit (ICU) for ARI. | **INCLUDED**: Standard epidemiological severe outcome. In primary care CMRs, admissions and diagnoses may both be recorded, but they are not easily linked, making ARI-specific attribution difficult. Captured via hospital discharge summaries, so reporting is not timely. |
| Intensive Care Unit Length of Stay (ICU Length of Stay) (5/139, 4%) [28, 30, 34, 121, 123] | Duration of time spent in ICU for an ARI. | **EXCLUDED**: Not routinely recorded in the primary care CMR therefore excluded. |
| Mechanical Ventilation (48/139, 35%) [17–20, 22, 27, 28, 30, 34, 35, 38–43, 45, 48, 49, 51, 52, 54, 56, 57, 60–63, 65–67, 70, 71, 76, 78, 80, 82, 83, 85–87, 91, 93, 115, 121–123] | Either non-invasive or invasive ventilator support. | **EXCLUDED**: Not routinely recorded in the primary care CMR therefore excluded. |
| Duration of Ventilation (2/139, 1%) [48, 66] | Duration of time spent on mechanical ventilation for an ARI. | **EXCLUDED**: Not routinely recorded in the primary care CMR therefore excluded. |
| Inotropic Support (2/139, 1%) [19, 35] | Inotropic support for critically ill patients. | **EXCLUDED**: Not routinely recorded in the primary care CMR therefore excluded. |
| Extracorporeal Membrane Oxygenation (ECMO) (7/139, 7%) [17, 27, 31, 40, 52, 60, 81] | A life-support measure that provides extracorporeal cardiac and respiratory support. | **EXCLUDED**: Not routinely recorded in the primary care CMR therefore excluded. |
| DEATH | | |
| Death (100/139, 72%) [17–30, 32, 34–38, 40, 42, 43, 45–49, 51–53, 56–73, 76–123] | -- | **INCLUDED**: Standard epidemiological severe outcome. Recorded in primary care CMRs, but attribution to ARI is uncertain as cause of death is not systematically coded. Deaths may be recorded more promptly than hospitalisations, since automatic systems exist in the NHS to capture deaths across all healthcare settings. |
| SYMPTOM | | |
| Cough (47/139, 34%) [17–21, 23, 24, 26, 28, 30, 32, 33, 38, 39, 43, 48, 50, 51, 53, 56, 65, 74, 76, 83, 84, 90, 92, 94, 96, 97, 99, 104, 107, 109, 112, 116, 121, 124–127] | -- | **EXCLUDED**: Not thought to be a strong enough discriminator of severity therefore excluded. |
| Productive Cough (12/139, 9%) [20, 22, 28, 53, 56, 65, 84, 92, 124, 125] | -- | **EXCLUDED**: Not thought to be a strong enough discriminator of severity therefore excluded. |
| Haemoptysis (7/139, 5%) [22, 24, 84, 93, 99] | -- | **INCLUDED**: May indicate more severe respiratory infection due to airway or lung tissue damage and associated inflammation. If recorded, it is usually done at the time of the ARI consultation, making it specific and timely. |
| Sore Throat (36/139, 26%) [17, 20, 22, 38, 56, 60, 92–94, 96, 124] | -- | **EXCLUDED**: Not thought to be a strong enough discriminator of severity therefore excluded. |
| Nasal Congestion and Rhinorrhoea (30/139, 22%) [17, 20, 22, 28, 33, 39, 50, 53, 56, 60, 65, 74, 84, 93, 96, 104, 107, 112, 113, 119, 124–126] | -- | **EXCLUDED**: Not thought to be a strong enough discriminator of severity therefore excluded. |
| Ear Pain (3/139, 2%) [74, 99] | -- | **EXCLUDED**: Not thought to be a strong enough discriminator of severity therefore excluded. |
| Chest Pain (15/139, 11%) [24, 38, 56, 74, 84, 90, 93, 96, 99, 107, 109, 124] | -- | **EXCLUDED**: Not thought to be a strong enough discriminator of severity therefore excluded. |
| Loss of Taste or Smell (8/139, 6%) [23, 30, 35, 65, 74, 90, 109, 124] | -- | **EXCLUDED**: Recognised indicator of possible SARS-CoV-2 infection, however this doesn’t necessarily indicate more severe infection, therefore excluded. |
| Dyspnoea (42/139, 30%) [17–24, 26, 28, 30, 39, 45, 50, 51, 53, 55, 56, 65, 74, 76, 83, 84, 90, 94, 96, 99, 104, 107, 109, 113, 116, 119, 121, 124, 125] | -- | **INCLUDED**: May indicate compromise of the respiratory system and hypoxia, making it a marker of more severe infection. If recorded, it is usually done at the time of the ARI consultation, making it specific and timely. |
| Fever (41/139, 29%) [17–19, 21, 23, 24, 26, 30, 32, 33, 38, 39, 43, 44, 48, 50, 51, 53, 56, 60, 65, 74, 76, 83, 90, 92, 93, 96, 97, 99, 107, 109, 112, 121, 124–126] | -- | **INCLUDED**: May indicate a degree of systemic upset and a more widespread response to infection. If recorded, it is usually captured at the time of the ARI consultation, making it specific and timely. Less value as a marker in paediatric patients (where fever is very common) and in older adults (who may not mount a febrile response). |
| Myalgia or Arthralgia (30/139, 22%) [17–19, 21, 22, 26, 28, 30, 33, 35, 53, 56, 60, 65, 74, 90, 94, 96, 99, 104, 107, 109, 112, 126, 127] | -- | **EXCLUDED**: Not thought to be a strong enough discriminator of severity therefore excluded. |
| Malaise and Loss of Appetite (23/139, 17%) [17, 21, 23, 26, 30, 39, 48, 53, 65, 74, 90, 92, 96, 99, 107, 109, 112, 116, 119, 124, 127] | -- | **INCLUDED**: May indicate systemic upset and more severe illness. If recorded, it is usually captured at the time of the ARI consultation, making it timely and specific. |
| Irritability (1/139, 1%) [107] | In a child | **EXCLUDED**: Likely marker of severity, but thought to be less likely recorded in the CMR. |
| Retroocular pain (1/139, 1%) [74] | -- | **EXCLUDED**: Not thought to be a strong enough discriminator of severity therefore excluded. |
| Confusion (3/139, 2%) [45, 53, 109] | -- | **INCLUDED**: May indicate systemic upset, especially in very young or older patients. Often linked with hypoxia, sepsis, or shock. If recorded, it is usually captured at the time of the ARI consultation, making it timely and specific. |
| Seizures (8/139, 6%) [22, 28, 33, 45, 48, 60] | Febrile convulsions | **EXCLUDED**: May be a complication of any febrile illness in child, but may not reflect severity. Although likely recorded less specific therefore excluded. |
| Diarrhoea (27/139, 19%) [19, 20, 32, 35, 39, 53, 56, 60, 65, 83, 84, 90, 92–94, 96, 99, 104, 109, 121, 124, 125, 127] | -- | **EXCLUDED**: Not thought to be a strong enough decimator of severity therefore excluded. |
| Nausea and Vomiting (27/139, 19%) [17, 19, 20, 22, 23, 28, 39, 48, 53, 60, 65, 74, 83, 90, 92–94, 99, 109, 116, 121, 127, 132] | -- | **EXCLUDED**: Not thought to be a strong enough decimator of severity therefore excluded. |
| Abdominal Pain (10/139, 7%) [17, 19, 20, 65, 74, 84, 96, 99, 109] | -- | **EXCLUDED**: Not thought to be a strong enough decimator of severity therefore excluded. |
| Duration of Illness (9/139, 6%) [46, 50, 56, 110, 119, 126, 127, 130] | -- | **EXCLUDED**: Not routinely recorded in the primary care CMR therefore excluded. |
| HEALTH SEEKING BEHAVIOUR | | |
| Health seeking behaviour (3/139, 2%) [44, 126, 128] | A patient seeks a consultation from a healthcare professional. | **INCLUDED**: Includes ambulance encounters or contact with NHS 111 (a free UK advice line). Seeking urgent or unscheduled care may indicate the patient or clinician perceived the illness as more severe. Likely recorded in primary care CMRs. Not ARI-specific, but included on balance. |
| ABSENCE | | |
| Work Absence (7/139, 5%) [44, 126, 127, 129–131] | Time taken off from work due to illness or medical reasons. | **INCLUDED**: Very likely recorded in primary care via electronic fit notes (Med3). Longer absences (e.g., >2 weeks) may reflect greater severity of illness. However, the reason for absence is not always identifiable, and this is only relevant for working-age adults. |
| School Absence (5/139, 4%) [44, 126, 130, 131] | Time taken off from school due to illness or medical reasons. | **EXCLUDED**: Very unlikely recorded as no statutory need to document therefore, excluded. |
| CLINICAL SIGNS | | |
| Body Temperature (17/139, 12%) [20, 32, 33, 43, 44, 51, 54, 62, 65, 79, 84, 94, 104, 113, 116, 132] | -- | **INCLUDED**: Fever or abnormal temperature is a recognised marker of systemic upset and can indicate systemic involvement. Likely to be recorded at the time of the ARI event in primary care, therefore timely. |
| Pulse Rate (6/139, 4%) [35, 45, 54, 62, 99] | -- | **INCLUDED**: Tachycardia or bradycardia is a recognised marker of physiological stress and systemic upset. Likely to be recorded at the time of the ARI event in primary care, therefore timely. |
| Respiratory Rate (14/139, 10%) [20, 22, 32, 35, 39, 45, 50, 51, 55, 62, 79, 84, 107, 132] | -- | **INCLUDED**: An abnormal respiratory rate is a recognised marker of systemic upset and respiratory compromise. Likely to be recorded at the time of the ARI event in primary care, therefore timely. |
| Oxygen Saturation (O2‚ Sat) (15/139, 11%) [19, 32, 35, 45, 50, 51, 55, 79, 99, 115, 116, 119, 132] | -- | **INCLUDED**: Oxygen saturation is an indirect measure of hypoxia and a key marker of respiratory compromise and systemic upset. Likely to be recorded at the time of the ARI event in primary care, therefore timely. |
| Blood Pressure (BP) (4/139, 3%) [24, 35, 45] | -- | **INCLUDED**: Blood pressure can indicate circulatory compromise in severe ARI. Likely recorded at the time of the ARI event in primary care, therefore timely, but not commonly recorded in children. |
| Work of Breathing (7/139, 5%) [48, 99, 116, 119, 132] | For example, use of accessory muscles, or grunting. Often referred to as respiratory distress in a child. | **INCLUDED**: Helps in the assessment of severity as increased effort (e.g., use of accessory muscles, grunting) indicates respiratory distress and possible hypoxia. Likely recorded at the time of the ARI event in primary care, therefore timely. |
| Chest Examination Findings (13/139, 9%) [20, 22, 28, 39, 45, 51, 54–56, 84, 97] | Presence of wheeze or crackles on clinical examination. | **INCLUDED**: Findings such as wheeze or crackles may indicate lower respiratory tract involvement and possible complications like pneumonia, indicating greater severity. Likely recorded at time of the ARI consultation in primary care, and therefore timely. |
| Cyanosis (4/139, 3%) [45, 48, 96, 107] | -- | **INCLUDED**: Indicates significant hypoxia and is therefore a marker of severe disease. Although relatively uncommon in primary care, if present it is likely to be recorded at the time of the ARI consultation, making it timely. |
| CLINICAL SCORES | | |
| Modified Early Warning Score (MEWS) (1/139, 1%) [133] | **Acute illness score:** A tool used in hospitals to detect early deterioration in patients by monitoring vital signs. Includes a range of related scores MEWS, NEWS, NEWS2, PEWS. | **INCLUDED:** Captures severity through changes in multiple vital signs and is widely used in hospitals. In the NHS, **National Early Warning Score 2 (NEWS2)** has replaced MEWS as the standard tool for detecting critical illness. Less commonly recorded in primary care, but included due to its relevance as a cross-sector severity measure. |
| Glasgow Coma Scale (GCS) (1/139, 1%) [101] | **Acute consciousness:** A neurological scale used to assess consciousness level in brain injury patients. | **INCLUDED:** A neurological scale assessing level of consciousness. Severe reductions in GCS reflect significant systemic or neurological compromise and therefore serve as a strong indicator of severity. Commonly used across the NHS, though less frequently recorded in primary care. Included due to its clear role as a severity marker. |
| Sequential Organ Failure Assessment score (SOFA) (3/139, 2%) [35, 121, 123] | **Critical illness score:** A scoring system that evaluates organ dysfunction in critically ill patients, predicting morbidity and mortality. | **EXCLUDED:** very unlikely used in primary care therefore excluded. |
| Simplified Acute Physiology Score II (SAPS II) (1/139, 1%) [121] | **Critical illness score:** A critical care scoring system [121] to assess illness severity and predict mortality in ICU patients. | **EXCLUDED:** very unlikely used in primary care therefore excluded. |
| Acute Physiology and Chronic Health Evaluation IV (APACHE IV) (1/139, 1%) [101] | **Critical illness score:** An ICU severity scoring system predicting patient outcomes based on physiological parameters and chronic health status. | **EXCLUDED:** very unlikely used in primary care therefore excluded. |
| Paediatric Risk of Mortality III Score (PRISM III) (1/139, 1%) [34] | **Critical illness score:** A scoring system used in paediatric intensive care units (PICUs) to predict mortality risk. | **EXCLUDED:** very unlikely used in primary care therefore excluded. |
| Barthel Index (1/139, 1%) [73] | **Activities of daily living:** A scale measuring performance in activities of daily living, used for rehabilitation and disability assessment. | **EXCLUDED:** very unlikely used in primary care therefore excluded. |
| EuroQol EQ-5D (1/139, 1%) [74] | **Chronic/acute quality of life:** A standardized instrument measuring health-related quality of life. | **EXCLUDED:** very unlikely used in primary care therefore excluded. |
| ViVI Disease Severity Score (1/139, 1%) [135] | **Acute influenza severity score:** A clinical severity assessment tool used in influenza infections to stratify patient risk. | **EXCLUDED:** very unlikely used in primary care therefore excluded. |
| Paediatric Chinese Medical Association Score (1/139, 1%) [19] | **COVID specific scoring:** A severity assessment tool used in medical settings in China. | **EXCLUDED:** very unlikely used in primary care therefore excluded. |
| Influenza Symptom Severity Scale (ISS) (1/139, 1%) [112] | **Acute quality of life (QoL):** Symptom score and patient impact assessment, an acute QoL assessment tool. A scoring system designed to quantify the severity of influenza-like symptoms. | **EXCLUDED:** very unlikely used in primary care therefore excluded. |
| World Health Organization (WHO) severity score (2/139, 1%) [35, 134] | **COVID specific scoring:** A classification system used to assess disease severity based on WHO guidelines. | **EXCLUDED:** very unlikely used in primary care therefore excluded. |
| American Academy Paediatrics Severity Score (1/139, 1%) [44] | **Acute influenza severity score:** A scoring system used to classify severity in paediatric conditions. | **EXCLUDED:** very unlikely used in primary care therefore excluded. |
| Project-Specific Composite Score (10/139, 7%) [27, 32, 36, 50, 86, 90, 124, 132, 142] | A composite of individual severity markers e.g. a collection symptoms (but not a formal score) or hospitalisation and death combined. | **EXCLUDED:** very unlikely used in primary care therefore excluded. However, could be theoretically constructed from a combination of other severity markers, therefore excluded in its own right. |
| INVESTIGATION | | |
| White blood cell count (11/139, 8%) [19, 20, 34, 45, 48, 50, 51, 53–55, 107] | Total circulating leukocytes (with differential). | **INCLUDED:** Elevated or abnormal WBC can indicate systemic infection or more severe inflammatory response. If undertaken in primary care, results are recorded in CMRs, but often become available some time after the ARI consultation, reducing timeliness and specificity. |
| Inflammatory markers (5/139, 4%) [19, 20, 45, 50, 53] | Acute phase reactants such as C-reactive protein (CRP) or erythrocyte sedimentation rate (ESR). Often elevated during in infections- typically in bacterial infections. | **INCLUDED:** Elevated inflammatory markers indicate systemic infection or more severe inflammatory response. If undertaken in primary care, results are recorded in CMRs, but often become available some time after the ARI consultation, reducing timeliness and specificity. |
| Renal function tests (3/139, 2%) [19, 25, 45] | Markers of acute kidney injury. | **EXCLUDED**: Less specific and commonly ordered for other reasons therefore excluded. |
| Liver function tests (2/139, 1%) [19, 45] | Markers of acute liver injury. | **EXCLUDED**: Less specific and commonly ordered for other reasons therefore excluded. |
| Fibrinogen (1/139, 1%) [19] | Acute-phase coagulation protein. Marker be elevated in infection or reduced in disseminated intravascular coagulation (DIC). | **EXCLUDED**: Very rarely recorded in primary care therefore excluded. |
| Procalcitonin (1/139, 1%) [19] | Pro-hormone released in bacterial (but not viral) infections. | **EXCLUDED**: Very rarely recorded in primary care therefore excluded. |
| Arterial blood gas (1/139, 1%) [45] | Can determine the severity gas exchange abnormalities during respiratory infections or acidosis in severe illness. | **EXCLUDED**: Very rarely recorded in primary care therefore excluded. |
| Chest X-ray (9/139, 6%) [28, 41, 43, 48, 51, 54, 55, 84] | May identify consolidation or lung parenchymal changes associated respiratory disease. | **INCLUDED:** Findings such as consolidation or parenchymal changes can indicate more severe respiratory disease (e.g., pneumonia). If requested in primary care, results are recorded in CMRs, though they are usually available after the ARI consultation, limiting timeliness and specificity. |
| TREATMENTS | | |
| Antibiotics (19/139, 14%) [20, 24, 32–34, 42, 44, 45, 49–51, 54, 55, 62, 71, 82, 121, 134] | Used to treat suspected bacterial infections including: Bacterial throat infections and pneumonia. | **INCLUDED**: Prescription of antibiotics often reflects increased clinical concern and may indicate more severe infection. Very well recorded in primary care CMRs and available in a timely manner. Common respiratory antibiotics include amoxicillin, penicillin V, macrolides, doxycycline, co-amoxiclav, and cephalosporins. |
| Antivirals (21/139, 15%) [17, 20, 27, 34, 44, 51, 52, 60, 61, 64, 76, 92, 93, 96, 104, 107, 122, 127, 135] | Used in the treatment of viral infections including influenza (e.g., oseltamivir) or SARS-CoV-2 infection (e.g., nirmatrelvir + ritonavir) | **INCLUDED**: Prescription of antivirals may indicate increased severity, depending on the specific drug. Generally well recorded in primary care CMRs and available in a timely manner. SARS-CoV-2 antivirals were excluded as they are rarely prescribed in primary care. |
| Steroids (1/139, 1%) [79] | Used in treatment of a number of acute respiratory conditions. For example, exacerbations of chronic lung disease, croup and severe SARS-CoV-2 infection. | **INCLUDED**: Oral steroids are commonly prescribed in primary care for certain ARIs (e.g., exacerbations of asthma or COPD, and croup). Their use may indicate greater severity. They are highly likely to be recorded in CMRs in a timely manner. |

## References

1. D’Ortenzio E, Renault P, Jaffar-Bandjee MC, et al (2010) S005: A review of the dynamics and severity of the pandemic A(H1N1) influenza virus on Réunion island, 2009. Clin Microbiol Infect 16:309–316

2. Luque-Paz D, Tattevin P, Loubet P, et al (2022) S015: Chronic use of inhaled corticosteroids in patients admitted for respiratory virus infections: a 6-year prospective multicenter study. Sci Rep 12:4199

3. Singh P, Attri K, Mahto D, et al (2021) S016: Clinical Profile of COVID-19 Illness in Children—Experience from a Tertiary Care Hospital. Indian J Pediatr 89:45

4. Huai Y, Guan X, Liu S, et al (2017) S020: Clinical characteristics and factors associated with severe acute respiratory infection and influenza among children in Jingzhou, China. Influenza Other Respir Viruses 11:148–156

5. Loubet P, Lenzi N, Valette M, et al (2017) S021: Clinical characteristics and outcome of respiratory syncytial virus infection among adults hospitalized with influenza-like illness in France. Clin Microbiol Infect 23:253–259

6. Hatem A, Mohamed S, Abu Elhassan UE, Ismael EAM, Rizk MS, El-Kholy A, El-Harras M (2019) S023: Clinical characteristics and outcomes of patients with severe acute respiratory infections (SARI): results from the Egyptian surveillance study 2010–2014. Multidiscip Respir Med 14:11

7. Cauchi JP, Borg ML, Džiugytė A, et al (2022) S033: Digitalizing and Upgrading Severe Acute Respiratory Infections Surveillance in Malta: System Development. JMIR Public Health Surveill. https://doi.org/10.2196/37669

8. Faramarzi H, Mousavi-Roknabadi RS, Hemmati A, Faramarzi A, Bakhtiari H (2021) S044: Epidemiology of Influenza in Fars Province, Southern Iran; a Population-Based Study (2015-2019). Arch Iran Med 24:199–208

9. Puig-Barberà J, Natividad-Sancho A, Trushakova S, et al (2016) S046: Epidemiology of Hospital Admissions with Influenza during the 2013/2014 Northern Hemisphere Influenza Season: Results from the Global Influenza Hospital Surveillance Network. PLoS One. https://doi.org/10.1371/JOURNAL.PONE.0154970

10. Bénézit F, Loubet P, Galtier F, et al (2020) S078: Non-influenza respiratory viruses in adult patients admitted with influenza-like illness: a 3-year prospective multicenter study. Infection 48:489–495

11. Lynfield R, Davey R, Dwyer DE, et al (2014) S082: Outcomes of influenza A(H1N1)pdm09 virus infection: results from two international cohort studies. PLoS One. https://doi.org/10.1371/JOURNAL.PONE.0101785

12. Abu Elhassan UE, Mohamed SAA, Rizk MS, Sherif M, El-Harras M (2020) S083: Outcomes of patients with Severe Acute Respiratory Infections (SARI) admitted to the intensive care unit: Results from the Egyptian Surveillance Study 2010-2014. Multidiscip Respir Med. https://doi.org/10.4081/MRM.2020.465

13. Fuentes Y V., Ibáñez-Prada ED, Serrano-Mayorga CC, et al (2022) S089: Prevalence, incidence, and severity associated with viral respiratory tract infections in Colombian adults before the COVID-19 pandemic. J Infect Public Health 15:1381–1387

14. S098: Sentiworld - Welcome ! https://sentiworld.sentiweb.fr/. Accessed 25 Feb 2025

15. Tramuto F, Restivo V, Costantino C, Colomba GME, Maida CM, Casuccio A, Vitale F (2019) S110: Surveillance Data for Eight Consecutive Influenza Seasons in Sicily, Italy. Am J Trop Med Hyg 101:1232–1239

16. Sakkou Z, Stripeli F, Papadopoulos NG, Critselis E, Georgiou V, Mavrikou M, Drossatou P, Constantopoulos A, Kafetzis D, Tsolia M (2011) S066: Impact of influenza infection on children’s hospital admissions during two seasons in Athens, Greece. Vaccine 29:1167–1172

17. Haddadin Z, Chappell J, McHenry R, et al (2021) S027: Coronavirus Surveillance in a Pediatric Population in Jordan From 2010 to 2013: A Prospective Viral Surveillance Study. Pediatr Infect Dis J 40:E12–E17

18. Shimada T, Sunagawa T, Taniguchi K, Yahata Y, Kamiya H, Yamamoto KU, Yasui Y, Okabe N (2015) S029: Description of hospitalized cases of influenza A(H1N1)pdm09 infection on the basis of the national hospitalized-case surveillance, 2009-2010, Japan. Jpn J Infect Dis 68:151–158

19. Rao S, Yanni E, Moss A, Lamb MM, Schuind A, Bekkat-Berkani R, Innis BL, Cotter J, Mistry RD, Asturias EJ (2019) S057: Evaluation of a New Clinical Endpoint for Moderate to Severe Influenza Disease in Children: A Prospective Cohort Study. J Pediatric Infect Dis Soc 9:460

20. Sakr Y, Ferrer R, Reinhart K, et al (2016) S117: The Intensive Care Global Study on Severe Acute Respiratory Infection (IC-GLOSSARI): a multicenter, multinational, 14-day inception cohort study. Intensive Care Med 42:817

21. Loevinsohn G, Hamahuwa M, Sinywimaanzi P, et al (2021) S058: Facility-based surveillance for influenza and respiratory syncytial virus in rural Zambia. BMC Infect Dis 21:1–15

22. Miroballi Y, Baird JS, Zackai S, et al (2010) S079: Novel influenza A(H1N1) in a pediatric health care facility in New York City during the first wave of the 2009 pandemic. Arch Pediatr Adolesc Med 164:24–30

23. Hazra D, Chandy GM, Thanjavurkar A, Gunasekaran K, Nekkanti AC, Pal R, Moorthy M, Abhilash KPP (2023) S003: A clinico-epidemiological profile, coinfections and outcome of patients with Influenza Like Illnesses (ILI) presenting to the emergency department during the COVID-19 pandemic. J Family Med Prim Care 12:672–678

24. Lees EA, Carrol ED, Gerrard C, Hardiman F, Howel G, Timmis A, Thorburn K, Guiver M, McNamara PS (2014) S013: Characterisation of acute respiratory infections at a United Kingdom paediatric teaching hospital: observational study assessing the impact of influenza A (2009 pdmH1N1) on predominant viral pathogens. BMC Infect Dis. https://doi.org/10.1186/1471-2334-14-343

25. Quéromès G, Frobert E, Burtseva E, et al (2022) S018: Clinical and phylogenetic influenza dynamics for the 2019-20 season in the global influenza hospital surveillance network (GIHSN) - Pilot study. J Clin Virol. https://doi.org/10.1016/J.JCV.2022.105184

26. Sharma A, Kothari N, Goel AD, et al (2021) S024: Clinical features and mortality in COVID-19 SARI versus non COVID-19 SARI cases from Western Rajasthan, India. J Family Med Prim Care 10:3240

27. Iwane MK, Chaves SS, Szilagyi PG, et al (2013) S034: Disparities Between Black and White Children in Hospitalizations Associated With Acute Respiratory Illness and Laboratory-confirmed Influenza and Respiratory Syncytial Virus in 3 US Counties—2002–2009. Am J Epidemiol 177:656–665

28. Cohen C, Walaza S, Moyes J, et al (2015) S048: Epidemiology of severe acute respiratory illness (SARI) among adults and children aged ≥5 years in a high HIV-prevalence setting, 2009-2012. PLoS One. https://doi.org/10.1371/JOURNAL.PONE.0117716

29. Jules A, Grijalva CG, Zhu Y, Talbot KH, Williams J V., Dupont WD, Edwards KM, Schaffner W, Shay DK, Griffin MR (2012) S053: Estimating age-specific influenza-related hospitalization rates during the pandemic (H1N1) 2009 in Davidson Co, TN. Influenza Other Respir Viruses. https://doi.org/10.1111/J.1750-2659.2012.00343.X

30. Chilean Task Force for study of Pandemic Influenza A (H1N1) C, Pedroni E, García M, et al (2010) S081: Outbreak of 2009 pandemic influenza A(H1N1), Los Lagos, Chile, April-June 2009. Eurosurveillance. https://doi.org/10.2807/ESE.15.01.19456-EN

31. Walaza S, Tempia S, von Gottberg A, et al (2022) S093: Risk Factors for Severe Coronavirus Disease 2019 Among Human Immunodeficiency Virus-Infected and -Uninfected Individuals in South Africa, April 2020-March 2022: Data From Sentinel Surveillance. Open Forum Infect Dis. https://doi.org/10.1093/OFID/OFAC578

32. Tempia S, Walaza S, Moyes J, et al (2017) S094: Risk Factors for Influenza-Associated Severe Acute Respiratory Illness Hospitalization in South Africa, 2012-2015. Open Forum Infect Dis. https://doi.org/10.1093/OFID/OFW262

33. El Kholy AA, Mostafa NA, Ali AA, El-Sherbini SA, Ismail RI, Magdy RI, Soliman MS, Said MM (2014) S095: Risk factors of prolonged hospital stay in children with viral severe acute respiratory infections. J Infect Dev Ctries 8:1285–1293

34. Cohen C, Moyes J, Tempia S, et al (2013) S105: Severe influenza-associated respiratory infection in high HIV prevalence setting, South Africa, 2009-2011. Emerg Infect Dis 19:1766–1774

35. Wishaupt JO, van der Ploeg T, de Groot R, Versteegh FGA, Hartwig NG (2017) S107: Single-and multiple viral respiratory infections in children: Disease and management cannot be related to a specific pathogen. BMC Infect Dis 17:1–11

36. Verani JR, McCracken J, Arvelo W, et al (2013) S111: Surveillance for Hospitalized Acute Respiratory Infection in Guatemala. PLoS One 8:e83600

37. Meerhoff TJ, Simaku A, Ulqinaku D, et al (2015) S112: Surveillance for severe acute respiratory infections (SARI) in hospitals in the WHO European region - an exploratory analysis of risk factors for a severe outcome in influenza-positive SARI cases. BMC Infect Dis. https://doi.org/10.1186/S12879-014-0722-X

38. Guerra-de-Blas P del C, Ortega-Villa AM, Ortiz-Hernández AA, et al (2023) S056: Etiology, clinical characteristics, and risk factors associated with severe influenza-like illnesses in Mexican adults. IJID regions 6:152–158

39. Le MN, Yoshida LM, Suzuki M, Nguyen HA, Le HT, Moriuchi H, Dang DA, Ariyoshi K (2014) S065: Impact of 2009 pandemic influenza among Vietnamese children based on a population-based prospective surveillance from 2007 to 2011. Influenza Other Respir Viruses 8:389–396

40. Yoshihara K, Le MN, Toizumi M, et al (2019) S070: Influenza B associated paediatric acute respiratory infection hospitalization in central vietnam. Influenza Other Respir Viruses 13:248–261

41. Kamara IF, Kumar AMV, Maruta A, et al (2022) S007: Antibiotic Use in Suspected and Confirmed COVID-19 Patients Admitted to Health Facilities in Sierra Leone in 2020–2021: Practice Does Not Follow Policy. Int J Environ Res Public Health 19:4005

42. Zakaria D, Aziz S, Bartholomew S, Park S Bin, Robitaille C, Weeks M (2023) S008: Associations between chronic conditions and death in hospital among adults (aged 20+ years) during first acute care hospitalizations with a confirmed or suspected COVID-19 diagnosis in Canada. PLoS One. https://doi.org/10.1371/JOURNAL.PONE.0280050

43. Ascencio-Montiel I de J, Ovalle-Luna OD, Rascón-Pacheco RA, Borja-Aburto VH, Chowell G (2022) S025: Comparative epidemiology of five waves of COVID-19 in Mexico, March 2020–August 2022. BMC Infect Dis 22:1–11

44. Chowell G, Echevarría-Zuno S, Viboud C, Simonsen L, Miller MA, Fernández-Gárate I, González-Bonilla C, Borja-Aburto VH (2012) S038: Epidemiological characteristics and underlying risk factors for mortality during the autumn 2009 pandemic wave in Mexico. PLoS One. https://doi.org/10.1371/JOURNAL.PONE.0041069

45. Cummings MJ, Bakamutumaho B, Kayiwa J, et al (2016) S036: Epidemiologic and Spatiotemporal Characterization of Influenza and Severe Acute Respiratory Infection in Uganda, 2010-2015. Ann Am Thorac Soc 13:2159–2168

46. Thielen BK, Friedlander H, Bistodeau S, et al (2017) S031: Detection of Influenza C Viruses Among Outpatients and Patients Hospitalized for Severe Acute Respiratory Infection, Minnesota, 2013–2016. Clin Infect Dis 66:1092

47. Fahim M, Roshdy WH, Deghedy O, et al (2022) S049: Epidemiology, Disease Severity and Outcome of Severe Acute Respiratory Syndrome Coronavirus 2 and Influenza Viruses Coinfection Seen at Egypt Integrated Acute Respiratory Infections Surveillance, 2020-2022. Can J Infect Dis Med Microbiol. https://doi.org/10.1155/2022/7497500

48. Buda S, Tolksdorf K, Schuler E, Kuhlen R, Haas W (2017) S051: Establishing an ICD-10 code based SARI-surveillance in Germany - description of the system and first results from five recent influenza seasons. BMC Public Health. https://doi.org/10.1186/S12889-017-4515-1

49. Puig-Barberà J, Tormos A, Sominina A, Burtseva E, Launay O, Ciblak MA, Natividad-Sancho A, Buigues-Vila A, Martínez-Úbeda S, Mahé C (2014) S061: First-year results of the Global Influenza Hospital Surveillance Network: 2012-2013 Northern hemisphere influenza season. BMC Public Health. https://doi.org/10.1186/1471-2458-14-564

50. Cohen C, Moyes J, Tempia S, et al (2015) S075: Mortality amongst Patients with Influenza-Associated Severe Acute Respiratory Illness, South Africa, 2009-2013. PLoS One 10:e0118884

51. Andrew MK, MacDonald S, Godin J, et al (2021) S087: Persistent Functional Decline Following Hospitalization with Influenza or Acute Respiratory Illness. J Am Geriatr Soc 69:696–703

52. Petrović V, Šeguljev Z, Ćosić G, Ristić M, Nedeljković J, Dragnić N, Ukropina S (2011) S084: Overview of the winter wave of 2009 pandemic influenza A(H1N1)v in Vojvodina, Serbia. Croat Med J 52:141–150

53. Chakhunashvili G, Wagner AL, Power LE, et al (2018) S099: Severe Acute Respiratory Infection (SARI) sentinel surveillance in the country of Georgia, 2015-2017. PLoS One. https://doi.org/10.1371/JOURNAL.PONE.0201497

54. Gachari MN, Ndegwa L, Emukule GO, Kirui L, Kalani R, Juma B, Mayieka L, Kinuthia P, Widdowson MA, Chaves SS (2022) S103: Severe acute respiratory illness surveillance for influenza in Kenya: Patient characteristics and lessons learnt. Influenza Other Respir Viruses 16:740–748

55. Al-Awaidy S, Hamid S, Al Obaidani I, et al (2015) S115: The Burden of Influenza-Associated Hospitalizations in Oman, January 2008-June 2013. PLoS One 10:e0144186

56. Emukule GO, Otiato F, Nyawanda BO, et al (2019) S116: The Epidemiology and Burden of Influenza B/Victoria and B/Yamagata Lineages in Kenya, 2012–2016. Open Forum Infect Dis 6:ofz421

57. Freitas ARR, Beckedorff OA, Cavalcanti LP de G, Siqueira AM, Castro DB de, Costa CF da, Lemos DRQ, Barros ENC (2021) S118: The emergence of novel SARS-CoV-2 variant P.1 in Amazonas (Brazil) was temporally associated with a change in the age and sex profile of COVID-19 mortality: A population based ecological study. The Lancet Regional Health - Americas 1:0

58. Yu H, Huang J, Huai Y, et al (2014) S122: The substantial hospitalization burden of influenza in central China: surveillance for severe, acute respiratory infection, and influenza viruses, 2010-2012. Influenza Other Respir Viruses 8:53–65

59. Dalton CB, Carlson SJ, Butler MT, Elvidge E, Durrheim DN (2013) S009: Building influenza surveillance pyramids in near real time, Australia. Emerg Infect Dis 19:1863–1865

60. Perez A, Lively JY, Curns A, et al (2022) S010: Respiratory Virus Surveillance Among Children with Acute Respiratory Illnesses — New Vaccine Surveillance Network, United States, 2016–2021. MMWR Morb Mortal Wkly Rep 71:1253–1259

61. Vette K, Bareja C, Clark R, Lal A (2018) S052: Establishing thresholds and parameters for pandemic influenza severity assessment, Australia. Bull World Health Organ 96:558

62. Pelat C, Lasserre A, Xavier A, Turbelin C, Blanchon T, Hanslik T (2013) S064: Hospitalization of influenza-like illness patients recommended by general practitioners in France between 1997 and 2010. Influenza Other Respir Viruses 7:74–84

63. Azziz-Baumgartner E, Cabrera AM, Chang L, et al (2012) S076: Mortality, Severe Acute Respiratory Infection, and Influenza-Like Illness Associated with Influenza A(H1N1)pdm09 in Argentina, 2009. PLoS One 7:e47540

64. Kang SH, Cheong HJ, Song JY, et al (2016) S006: Analysis of Risk Factors for Severe Acute Respiratory Infection and Pneumonia and among Adult Patients with Acute Respiratory Illness during 2011-2014 Influenza Seasons in Korea. Infect Chemother 48:294

65. Chowell G, Echevarría-Zuno S, Viboud C, Simonsen L, Tamerius J, Miller MA, Borja-AburtoVí VH (2011) S014: Characterizing the Epidemiology of the 2009 Influenza A/H1N1 Pandemic in Mexico. PLoS Med 8:e1000436

66. Yordanov Y, Dinh A, Bleibtreu A, Mensch A, Lescure FX, Debuc E, Jourdain P, Jaulmes L, Dechartres A (2021) S019: Clinical characteristics and factors associated with hospital admission or death in 43 103 adult outpatients with coronavirus disease 2019 managed with the Covidom telesurveillance solution: a prospective cohort study. Clinical Microbiology and Infection 27:1158

67. Galindo-Fraga A, Ortiz-Hernández AA, Ramírez-Venegas A, et al (2013) S022: Clinical characteristics and outcomes of influenza and other influenza-like illnesses in Mexico City. Int J Infect Dis. https://doi.org/10.1016/J.IJID.2013.01.006

68. P Efstathiou, M Tseroni, A Baka S028: Deaths and Hospitalizations Related to 2009 Pandemic Influenza A (H1N1) --- Greece, May 2009--February 2010. https://www.cdc.gov/mmwr/preview/mmwrhtml/mm5922a2.htm. Accessed 25 Feb 2025

69. Duarte MB, Gregianini TS, Martins LG, Veiga ABG (2021) S047: Epidemiology of influenza B infection in the state of Rio Grande do Sul, Brazil, from 2003 to 2019. J Med Virol 93:4756–4762

70. de Araujo KLR, de Aquino ÉC, da Silva LLS, Ternes YMF (2020) S059: Factors associated with Severe Acute Respiratory Syndrome in a Brazilian central region. Cien Saude Colet 25:4121–4130

71. Guo RN, Zheng HZ, Ou CQ, et al (2016) S067: Impact of Influenza on Outpatient Visits, Hospitalizations, and Deaths by Using a Time Series Poisson Generalized Additive Model. PLoS One 11:e0149468

72. Pung R, Lee VJM (2020) S068: Implementing the World Health Organization Pandemic Influenza Severity Assessment framework-Singapore’s experience. Influenza Other Respir Viruses 14:3–10

73. Obermeier PE, Seeber LD, Alchikh M, Schweiger B, Rath BA (2022) S069: Incidence, Disease Severity, and Follow-Up of Influenza A/A, A/B, and B/B Virus Dual Infections in Children: A Hospital-Based Digital Surveillance Program. Viruses. https://doi.org/10.3390/V14030603

74. Moretti ML, Sinkoc V, Cardoso LG de O, de Camargo GJ, Bachur LF, Hofling CC, Angerami R, Trabasso P, Garcia MT, Resende MR (2011) S072: Lessons from the epidemiological surveillance program, during the influenza A (H1N1) virus epidemic, in a reference university hospital of Southeastern Brazil. Rev Soc Bras Med Trop 44:405–411

75. Castillo-Palencia JP, Laflamme L, Monárrez-Espino J (2012) S080: Occurrence of AH1N1 viral infection and clinical features in symptomatic patients who received medical care during the 2009 influenza pandemic in Central Mexico. BMC Infect Dis. https://doi.org/10.1186/1471-2334-12-363/METRICS

76. Cazé AB, Cerqueira-Silva T, Bomfim AP, et al (2023) S088: Prevalence and risk factors for long COVID after mild disease: A cohort study with a symptomatic control group. J Glob Health 13:06015

77. Falsey AR, McElhaney JE, Beran J, et al (2014) S091: Respiratory syncytial virus and other respiratory viral infections in older adults with moderate to severe influenza-like illness. J Infect Dis 209:1873–1881

78. Perez-Padilla R, Garcia-Sancho C, Fernandez R, Franco-Marina F, Lopez-Gatell H, Bojorquez I (2013) S120: The impact of altitude on hospitalization and hospital mortality from pandemic 2009 influenza A (H1N1) virus pneumonia in Mexico. Salud Publica Mex 55:92–95

79. van Hoek AJ, Underwood A, Jit M, Miller E, Edmunds WJ (2011) S121: The impact of pandemic influenza H1N1 on health-related quality of life: a prospective population-based study. PLoS One. https://doi.org/10.1371/JOURNAL.PONE.0017030

80. Morgenstern-Kaplan D, Buitano-Tang B, Martínez-Gil M, Pavón AZP, Talavera JO (2020) S123: U-shaped-aggressiveness of SARS-CoV-2: Period between initial symptoms and clinical progression to COVID-19 suspicion. A population-based cohort study. PLoS One. https://doi.org/10.1371/JOURNAL.PONE.0243268

81. Boender TS, Cai W, Schranz M, et al (2022) S125: Using routine emergency department data for syndromic surveillance of acute respiratory illness, Germany, week 10 2017 until week 10 2021. Eurosurveillance 27:2100865

82. Lindblade KA, Arvelo W, Gray J, Estevez A, Frenkel G, Reyes L, Moscoso F, Moir JC, Fry AM, Olsen SJ (2010) S001: A Comparison of the Epidemiology and Clinical Presentation of Seasonal Influenza A and 2009 Pandemic Influenza A (H1N1) in Guatemala. PLoS One 5:e15826

83. De Carvalho FC, Da Silva ET, De Almeida WAF, Maroneze MA, Schwartz J de A, Jardim JPV, Peixoto HM (2023) S017: Clinical and epidemiological aspects of severe acute respiratory infection: before and during the first year of the COVID-19 pandemic in Brazil. Trans R Soc Trop Med Hyg 117:161–173

84. Rowlinson E, Peters L, Mansour A, et al (2021) S026: Comparison of common acute respiratory infection case definitions for identification of hospitalized influenza cases at a population-based surveillance site in Egypt. PLoS One. https://doi.org/10.1371/JOURNAL.PONE.0248563

85. Sansone NMS, Boschiero MN, Marson FAL (2022) S037: Epidemiologic Profile of Severe Acute Respiratory Infection in Brazil During the COVID-19 Pandemic: An Epidemiological Study. Front Microbiol. https://doi.org/10.3389/FMICB.2022.911036

86. Anders KL, Nguyen HL, Nguyen NM, et al (2015) S043: Epidemiology and virology of acute respiratory infections during the first year of life: a birth cohort study in Vietnam. Pediatr Infect Dis J 34:361–370

87. Das P, Akhtar Z, Mah-E-Muneer S, et al (2021) S050: Establishing a sentinel surveillance system for the novel COVID-19 in a resource-limited country: methods, system attributes and early findings. BMJ Open. https://doi.org/10.1136/BMJOPEN-2021-055169

88. Mohamed AM, Al Sayyad A, Matar E, Isa HM, Hasan WF, Hashim NSJY, Alajaimi BA, Aldolabi Q (2023) S060: Factors associated with poor outcomes in patients with severe acute respiratory infections in Bahrain. Influenza Other Respir Viruses. https://doi.org/10.1111/IRV.13133

89. Abdalla O, Mohammed M, Hakawi AM, Aljifri A, Abdalla M, Eltigani S, Mujib SA, Assiri A (2020) S063: Hospital-based surveillance of influenza A(H1N1)pdm09 virus in Saudi Arabia, 2010-2016. Ann Saudi Med 40:1–6

90. Kandeel A, Dawson P, Labib M, Said M, El-Refai S, El-Gohari A, Talaat M (2016) S074: Morbidity, Mortality, and Seasonality of Influenza Hospitalizations in Egypt, November 2007-November 2014. PLoS One 11:e0161301

91. Al-Abdallat M, Dawson P, Haddadin AJ, El-Shoubary W, Dueger E, Al-Sanouri T, Said MM, Talaat M (2016) S071: Influenza hospitalization epidemiology from a severe acute respiratory infection surveillance system in Jordan, January 2008-February 2014. Influenza Other Respir Viruses 10:91–97

92. S097: 2022 Acute respiratory illness surveillance report. https://www.esr.cri.nz/digital-library/2022-acute-respiratory-illness-surveillance-report/. Accessed 25 Feb 2025

93. Al Amad MA, Al Mahaqri AA, Al Serouri AA, Khader YS (2019) S100: Severe Acute Respiratory Infections With Influenza and Noninfluenza Respiratory Viruses: Yemen, 2011-2016. Inquiry. https://doi.org/10.1177/0046958019850731

94. Zhou JA, Schweinle JE, Lichenstein R, Walker RE, King JC (2020) S101: Severe Illnesses Associated With Outbreaks of Respiratory Syncytial Virus and Influenza in Adults. Clin Infect Dis 70:773–779

95. Padilla-Raygoza N, Sandoval-Salazar C, Ramírez-Gómez XS, Navarro-Olivos E, de Jesus Gallardo-Luna M, Magos-Vazquez FJ, Diaz-Martinez DA (2020) S108: Status of novel coronavirus disease and analysis of mortality in Mexico, until June 30th, 2020: An ecological study. Biomedical and Pharmacology Journal 13:1781–1790

96. Tramuto F, Maida CM, Bonura F, et al (2011) S113: Surveillance of hospitalised patients with influenza-like illness during pandemic influenza A(H1N1) season in Sicily, April 2009 - December 2010. Eurosurveillance 16:19957

97. Horton KC, Dueger EL, Kandeel A, et al (2017) S126: Viral etiology, seasonality and severity of hospitalized patients with severe acute respiratory infections in the Eastern Mediterranean Region, 2007–2014. PLoS One 12:e0180954

98. Murthy S (2019) S124: Using research to prepare for outbreaks of severe acute respiratory infection. BMJ Glob Health 4:1061

99. Leiner J, Hohenstein S, Pellissier V, König S, Winklmair C, Nachtigall I, Bollmann A, Kuhlen R (2023) S011: COVID-19 and Severe Acute Respiratory Infections: Monitoring Trends in 421 German Hospitals During the First Four Pandemic Waves. S011: Infection and drug resistance 16:2775–2781

100. Allam RR, Murhekar M V., Tadi GP, Udaragudi PR (2013) S030: Descriptive epidemiology of novel influenza A (H1N1), Andhra Pradesh 2009-2010. Indian J Public Health 57:161–165

101. Eybpoosh S, Afshari M, Haghdoost A-A, Afsar Kazerooni P, Gouya MM, Tayeri K (2021) S106: Severity and mortality of COVID-19 infection in HIV-infected individuals: Preliminary findings from Iran. Med J Islam Repub Iran. https://doi.org/10.47176/MJIRI.35.33

102. Seligová J, Čulmanová A, Krištufková Z, Čisláková L, Hudečková H (2011) S012: Changes in surveillance of acute respiratory infections including influenza in the slovak republic during 1993-2008. Cent Eur J Public Health 19:20–25

103. Borja-Aburto VH, Chowell G, Viboud C, Simonsen L, Miller MA, Grajales-Muñiz C, González-Bonilla CR, Diaz-Quiñonez JA, Echevarría-Zuno S (2012) S039: Epidemiological characterization of a fourth wave of pandemic A/H1N1 influenza in Mexico, winter 2011-2012: age shift and severity. Arch Med Res 43:563–570

104. Dwibedi B, Sabat J, Dixit S, et al (2019) S041: Epidemiological and clinical profile of Influenza A(H1N1) pdm09 in Odisha, eastern India. Heliyon 5:e02639

105. Liu Z, Gao L, Xue C, Zhao C, Liu T, Tia A, Wang L, Sun J, Li Z, Harding D (2022) S040: Epidemiological Trends of Coronavirus Disease 2019 in Sierra Leone From March 2020 to October 2021. Front Public Health. https://doi.org/10.3389/FPUBH.2022.949425

106. Fahim M, AbdElGawad B, Hassan H, Naguib A, Ahmed ES, Afifi S, Abu ElSood H, Mohsen A (2021) S042: Epidemiology and outcome of influenza-associated infections among hospitalized patients with acute respiratory infections, Egypt national surveillance system, 2016-2019. Influenza Other Respir Viruses 15:589–598

107. Van Asten L, Luna Pinzon A, De Lange DW, De Jonge E, Dijkstra F, Marbus S, Donker GA, Van Der Hoek W, De Keizer NF (2018) S054: Estimating severity of influenza epidemics from severe acute respiratory infections (SARI) in intensive care units. Crit Care. https://doi.org/10.1186/S13054-018-2274-8

108. Abdel-Hady DM, Al Balushi RM, Al Abri BA, Al Abri SS, Al Kindi HS, Al-Jardani AK, Al Yaqubi FM, Al Abaidani IS (2018) S055: Estimating the burden of influenza-associated hospitalization and deaths in Oman (2012-2015). Influenza Other Respir Viruses 12:146–152

109. Escobar AL, Rodriguez TDM, Monteiro JC (2020) S073: Lethality and characteristics of deaths due to COVID-19 in Rondônia: an observational study. Epidemiol Serv Saude. https://doi.org/10.1590/S1679-49742021000100019

110. Weng TC, Chiu HYR, Chen SY, Shih FY, King CC, Fang CC (2019) S077: National retrospective cohort study to identify age-specific fatality risks of comorbidities among hospitalised patients with influenza-like illness in Taiwan. BMJ Open. https://doi.org/10.1136/BMJOPEN-2018-025276

111. Oliveira W, Carmo E, Penna G, Kuchenbecker R, Santos H, Araujo W, Malaguti R, Duncan B, Schmidt M (2009) S085: Pandemic H1N1 influenza in Brazil: analysis of the first 34,506 notified cases of influenza-like illness with severe acute respiratory infection (SARI). Euro Surveill. https://doi.org/10.2807/ESE.14.42.19362-EN

112. Elimian KO, Ochu CL, Ebhodaghe B, et al (2020) S086: Patient characteristics associated with COVID-19 positivity and fatality in Nigeria: retrospective cohort study. BMJ Open. https://doi.org/10.1136/BMJOPEN-2020-044079

113. Chowell G, Echevarría-Zuno S, Viboud C, Simonsen L, Grajales Muñiz C, Rascón Pacheco RA, González León M, Borja Aburto VH (2012) S090: Recrudescent wave of pandemic A/H1N1 influenza in Mexico, winter 2011-2012: Age shift and severity. PLoS Curr 4:RRN1306

114. Akhtar Z, Islam MA, Aleem MA, et al (2021) S096: SARS-CoV-2 and influenza virus coinfection among patients with severe acute respiratory infection during the first wave of COVID-19 pandemic in Bangladesh: a hospital-based descriptive study. BMJ Open. https://doi.org/10.1136/BMJOPEN-2021-053768

115. McMorrow ML, Wemakoy EO, Tshilobo JK, et al (2015) S102: Severe Acute Respiratory Illness Deaths in Sub-Saharan Africa and the Role of Influenza: A Case Series From 8 Countries. J Infect Dis 212:853–860

116. Breiman RF, Cosmas L, Njenga MK, et al (2015) S104: Severe acute respiratory infection in children in a densely populated urban slum in Kenya, 2007-2011. BMC Infect Dis 15:1–11

117. Dávila J, Chowell G, Borja-Aburto VH, Viboud C, Grajales Muñiz C, Miller M (2014) S109: Substantial Morbidity and Mortality Associated with Pandemic A/H1N1 Influenza in Mexico, Winter 2013-2014: Gradual Age Shift and Severity. PLoS Curr. https://doi.org/10.1371/CURRENTS.OUTBREAKS.A855A92F19DB1D90CA955F5E908D6631

118. Sigmundsdottir G, Gudnason T, Ólafsson Ö, Baldvinsdóttir GE, Atladottir A, Löve A, Danon L, Briem H (2010) S114: Surveillance of influenza in Iceland during the 2009 pandemic. Euro Surveill 15:3

119. Caini S, de Mora D, Olmedo M, et al (2019) S119: The epidemiology and severity of respiratory viral infections in a tropical country: Ecuador, 2009-2016. J Infect Public Health 12:357–363

120. Viera-Segura O, Vega-Magaña N, García-Chagollán M, et al (2021) S002: A Comprehensive Descriptive Epidemiological and Clinical Analysis of SARS-CoV-2 in West-Mexico during COVID-19 Pandemic 2020. Int J Environ Res Public Health. https://doi.org/10.3390/IJERPH182010644

121. Wei L, Liu W, Zhang XA, Liu EM, Wo Y, Cowling BJ, Cao WC (2015) S032: Detection of Viral and Bacterial Pathogens in Hospitalized Children With Acute Respiratory Illnesses, Chongqing, 2009–2013. Medicine 94:e742

122. Stockwell MS, Reed C, Vargas CY, Wang L, Alba LR, Jia H, Larussa P, Larson EL, Saiman L (2022) S062: Five-Year Community Surveillance Study for Acute Respiratory Infections Using Text Messaging: Findings From the MoSAIC Study. Clin Infect Dis 75:987–995

123. Peterson I, Bar-Zeev N, Kennedy N, et al (2016) S092: Respiratory Virus–Associated Severe Acute Respiratory Illness and Viral Clustering in Malawian Children in a Setting With a High Prevalence of HIV Infection, Malaria, and Malnutrition. J Infect Dis 214:1700

124. Fragaszy EB, Warren-Gash C, White PJ, Zambon M, Edmunds WJ, Nguyen-Van-Tam JS, Hayward AC (2018) S035: Effects of seasonal and pandemic influenza on health-related quality of life, work and school absence in England: Results from the Flu Watch cohort study. Influenza Other Respir Viruses 12:171–182

125. Ho A, Mallewa J, Peterson I, et al (2018) S045: Epidemiology of Severe Acute Respiratory Illness and Risk Factors for Influenza Infection and Clinical Severity among Adults in Malawi, 2011–2013. Am J Trop Med Hyg 99:772

126. VanWormer JJ, Sundaram ME, Meece JK, Belongia EA (2014) S004: A cross-sectional analysis of symptom severity in adults with influenza and other acute respiratory illness in the outpatient setting. BMC Infect Dis 14:1–10
